# Supplementary material for: Synthesis of Hydantoin Androgen Receptor Antagonists and Study on Their Antagonistic Activity
Source: Molecules. 2022 Sep 10;27(18):5867. doi: 10.3390/molecules27185867 (PMC9505067; doi:10.3390/molecules27185867)
Supplement: Supplementary file 1 [file molecules-27-05867-s001.zip › molecules-1882537-supplementary.pdf]

Supporting Information

Synthesis of Hydantoin androgen Receptor Antagonists and Study on Their Antagonistic Activity

Longjun Ma [a,b], Yan Zhou [c], Dehua Yang [c], Ming-Wei Wang [c,*], Wei Lu [b,*] and Jiyu Jin [b,*]

[a] *Department of Applied Chemistry, School of Chemistry and Chemical Engineering, Lanzhou Jiaotong University, 88 Anning West Road, Lanzhou 730070, China*

[b] *Shanghai Engineering Research Center of Molecular Therapeutics and New Drug Development, School of Chemistry and Molecular Engineering, East China Normal University, 3663 North Zhongshan Road, Shanghai 200062, China*

[c] *The National Center for Drug Screening and the CAS Key Laboratory of Receptor Research, Shanghai Institute of Materia Medica, Chinese Academy of Sciences (CAS), 189 Guo Shou Jing Road, Shanghai 200031, China*

*Corresponding authors:

E-mail: mwwang@simm.ac.cn (M.-W.W.); wlu@chem.ecnu.edu.cn (W.L.); jyjin@chem.ecnu.edu.cn (J.J.)

Table of contents··················S 1

^{1}H and ^{13}C NMR spectra of *compound* **3a-l**··················S 2

^{1}H and ^{13}C NMR spectra of *compound* **5a-l**··················S 12

^{1}H and ^{13}C NMR spectra of *compound* **13-17**··················S 23

^{1}H and ^{13}C NMR spectra of *compound* **18a-d**··················S 28

^{1}H ^{13}C, NMR and HRMS spectra of *compound* **6a-l**··················S 32

^{1}H, ^{13}C, NMR and HRMS spectra of *compound* **7a-j**··················S 56

^{1}H, ^{13}C, NMR and HRMS spectra of *compound* **9**··················S 72

^{1}H, ^{13}C, NMR and HRMS spectra of *compound* **10**··················S 74

^{1}H, ^{13}C, NMR and HRMS spectra of *compound* **11a-b**··················S 76

^{1}H ^{13}C, NMR and HRMS spectra spectra of *compound* **19a-d**··················S 80

^{1}H and ^{13}C NMR spectra of *compound* **3a-l**

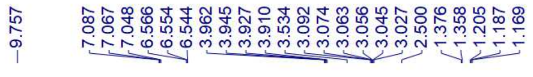

^{1}H NMR (400 MHz, DMSO) δ 7.07 (t, J = 7.8 Hz, 2H), 6.61 – 6.50 (m, 3H), 3.94 (q, J = 7.0 Hz, 0H), 1.37 (d, J = 7.0 Hz, 3H).

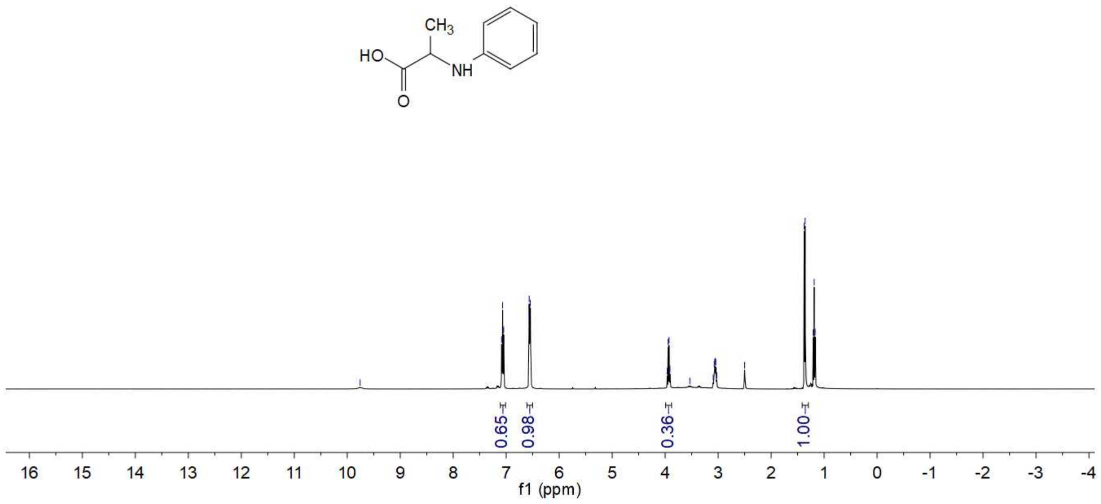

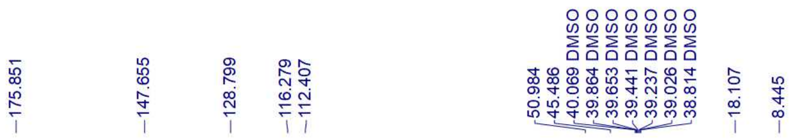

^{13}C NMR (101 MHz, DMSO) δ 175.85, 147.65, 128.80, 116.28, 112.41, 50.98, 45.49, 18.11, 8.45.

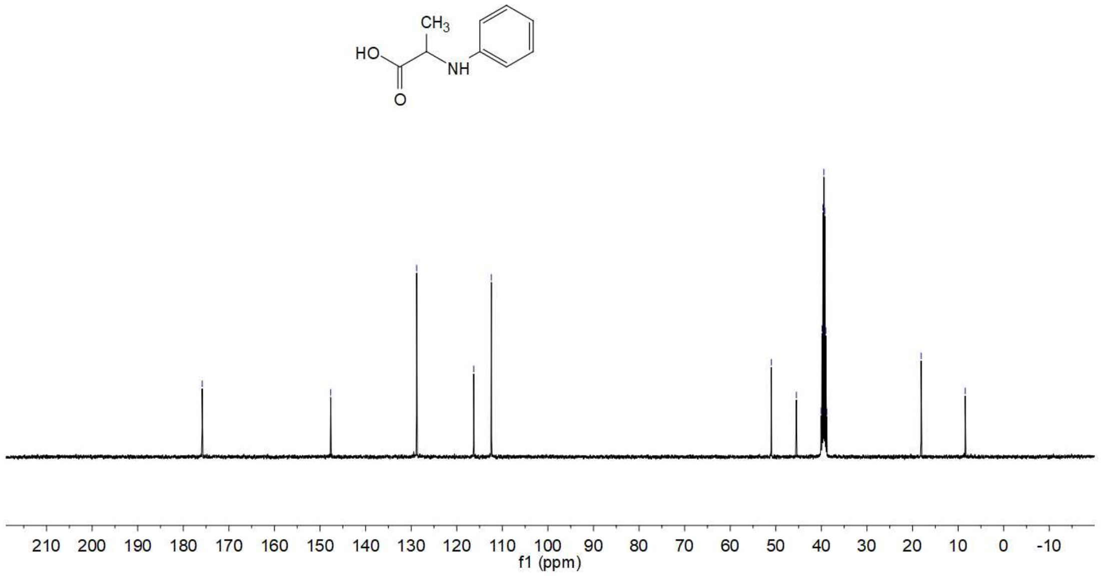

^{1}H NMR (400 MHz, DMSO) δ 6.88 (d, J = 8.2 Hz, 2H), 6.47 (d, J = 8.3 Hz, 2H), 3.90 (q, J = 7.0 Hz, 1H), 2.14 (s, 3H), 1.35 (d, J = 7.0 Hz, 3H).

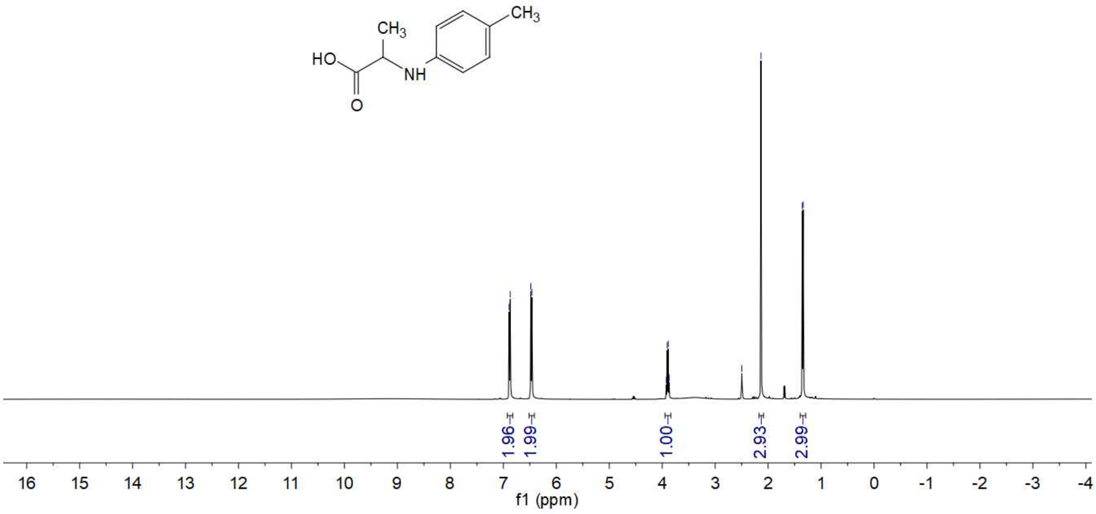

^{13}C NMR (101 MHz, DMSO) δ 175.97, 145.37, 129.24, 124.73, 112.62, 51.27, 20.01, 18.15.

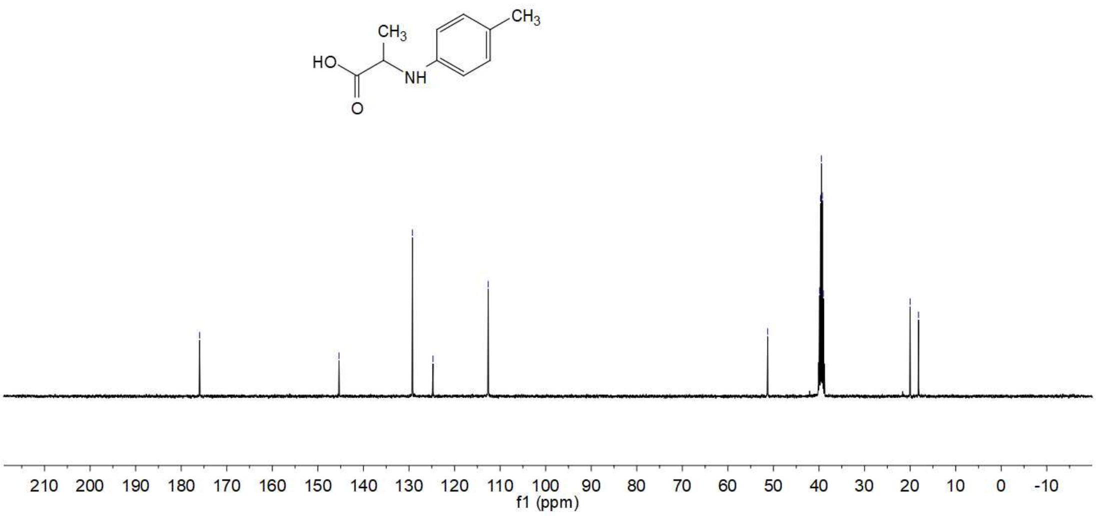

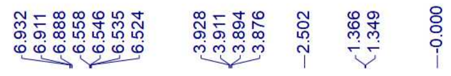

^{1}H NMR (400 MHz, DMSO) δ 6.91 (t, J = 8.8 Hz, 2H), 6.54 (dd, J = 8.9, 4.5 Hz, 2H), 3.90 (q, J = 7.0 Hz, 1H), 1.36 (d, J = 6.9 Hz, 3H).

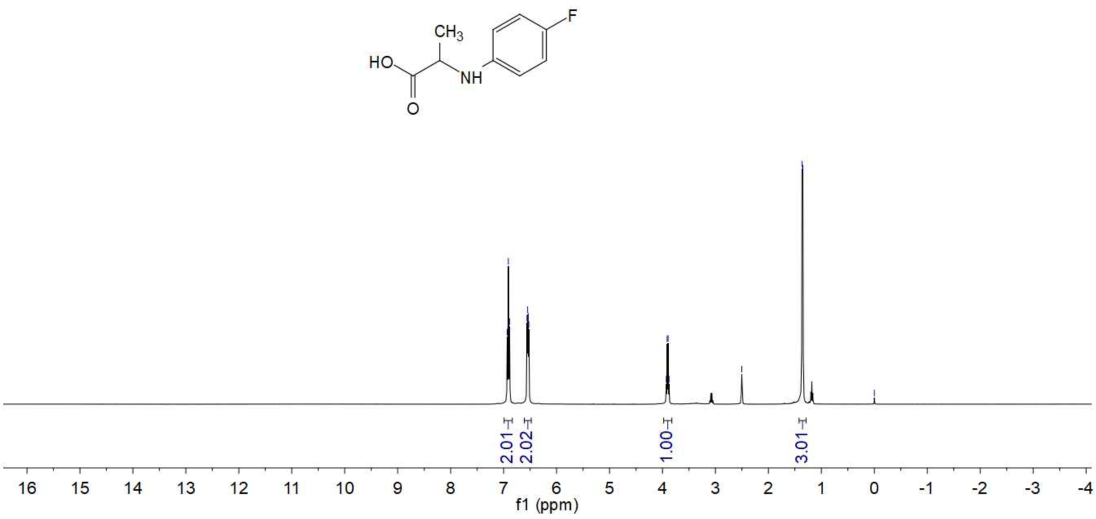

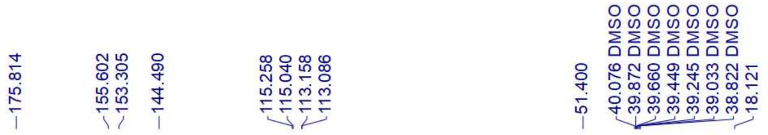

^{13}C NMR (101 MHz, DMSO) δ 175.81, 155.60, 153.31, 144.49, 115.26, 115.04, 113.16, 113.09, 51.40, 18.12.

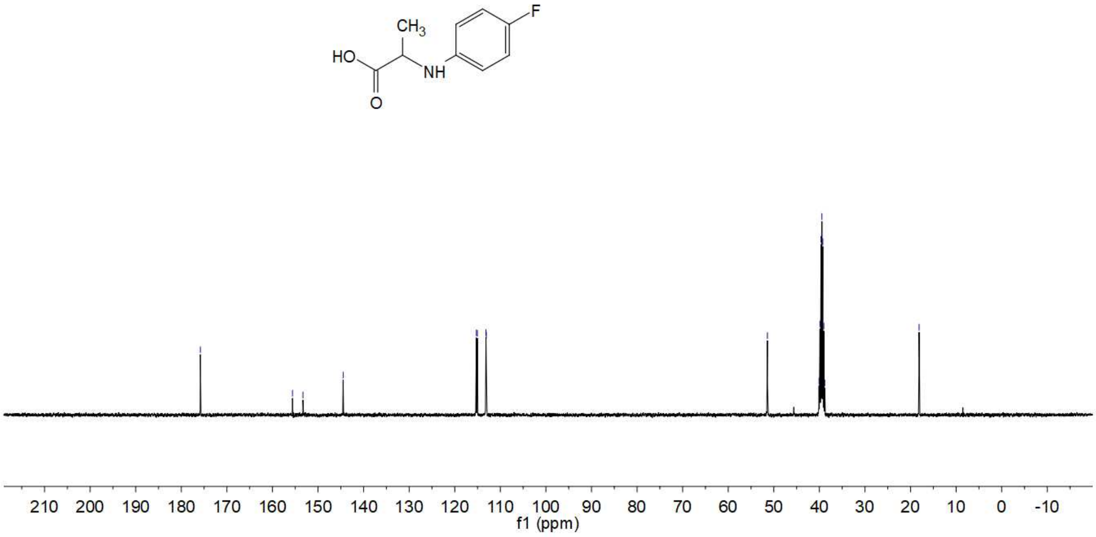

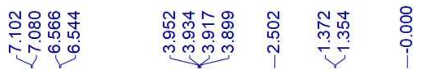

^1H NMR (400 MHz, DMSO) δ 7.09 (d, J = 8.8 Hz, 2H), 6.55 (d, J = 8.8 Hz, 2H), 3.93 (q, J = 7.0 Hz, 1H), 1.36 (d, J = 7.0 Hz, 3H).

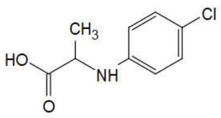

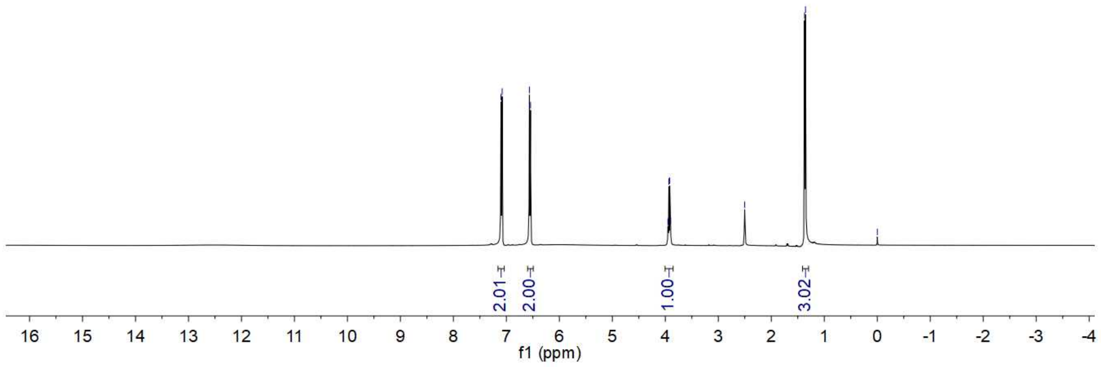

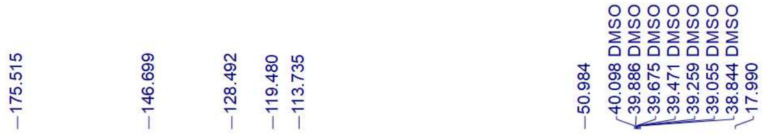

^{13}C NMR (101 MHz, DMSO) δ 175.52, 146.70, 128.49, 119.48, 113.73, 50.98, 17.99.

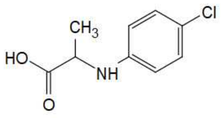

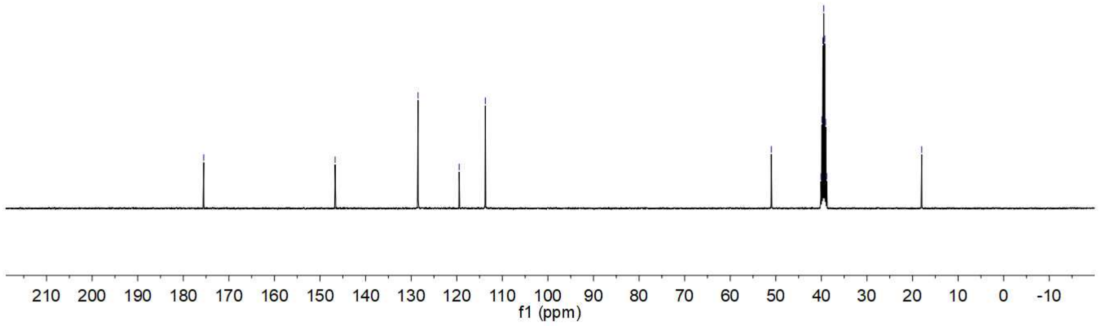

^{1}H NMR (400 MHz, DMSO) δ 12.53 (s, 1H), 7.29 – 7.11 (m, 2H), 6.58 – 6.45 (m, 2H), 3.92 (q, J = 7.0 Hz, 1H), 1.36 (d, J = 7.0 Hz, 3H).

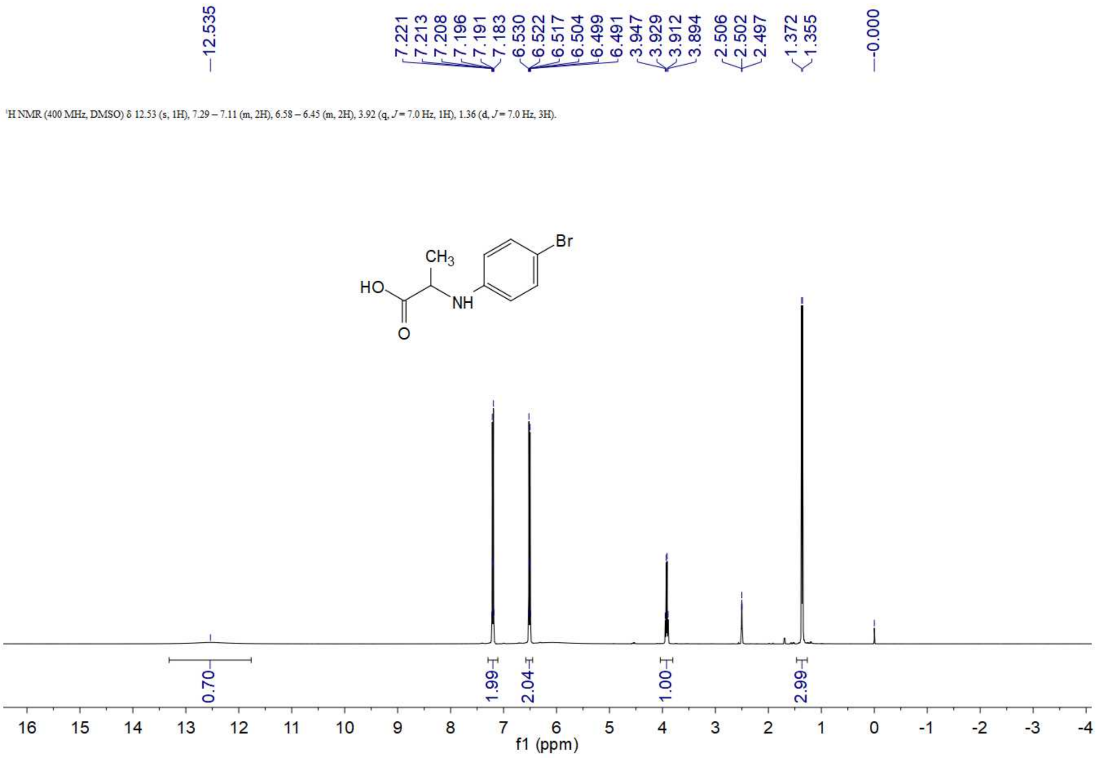

^{13}C NMR (101 MHz, DMSO) δ 175.49, 147.10, 131.31, 114.28, 106.83, 50.89, 17.98.

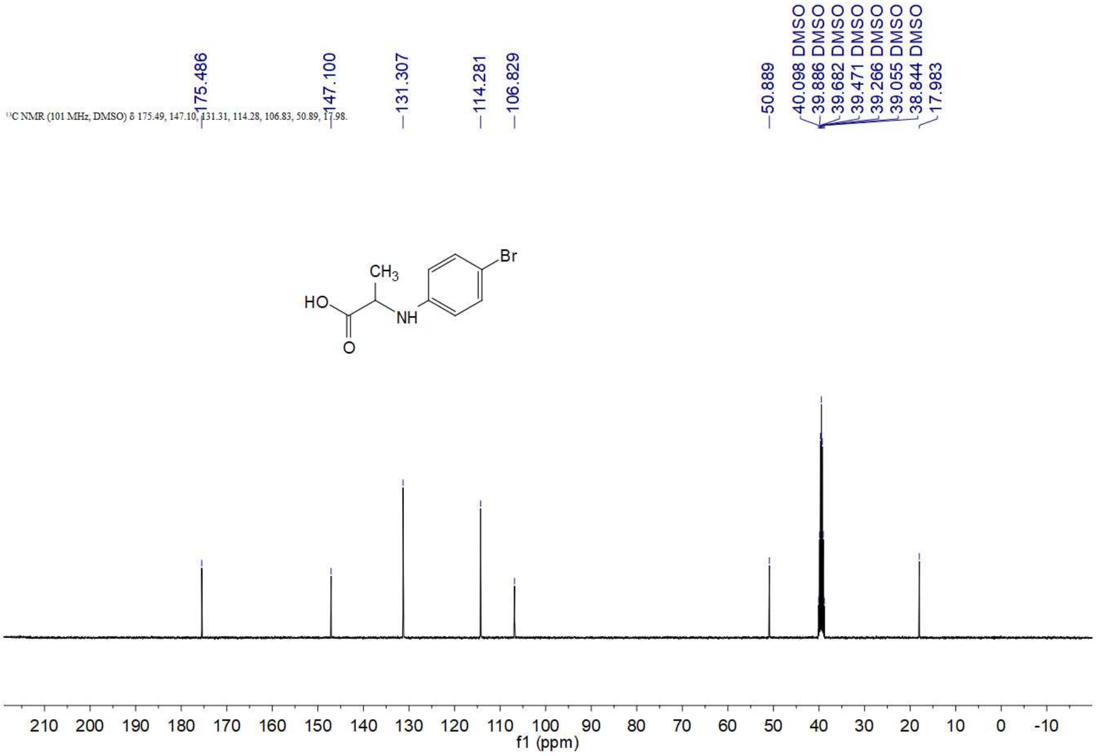

^{1}H NMR (400 MHz, DMSO) δ 8.89 (s, 1H), 6.71 (d, J = 8.8 Hz, 2H), 6.52 (d, J = 8.8 Hz, 2H), 3.87 (q, J = 6.9 Hz, 1H), 3.62 (s, 3H), 1.34 (d, J = 6.9 Hz, 3H).

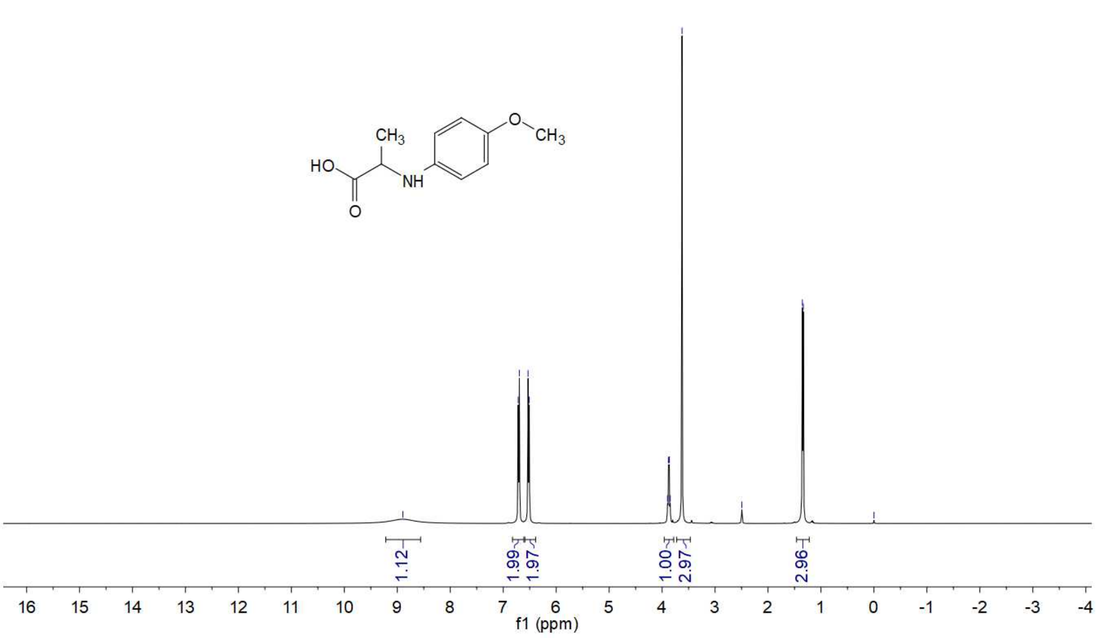

^{13}C NMR (101 MHz, DMSO) δ 176.15, 151.02, 141.92, 114.49, 113.53, 55.21, 51.71, 18.26.

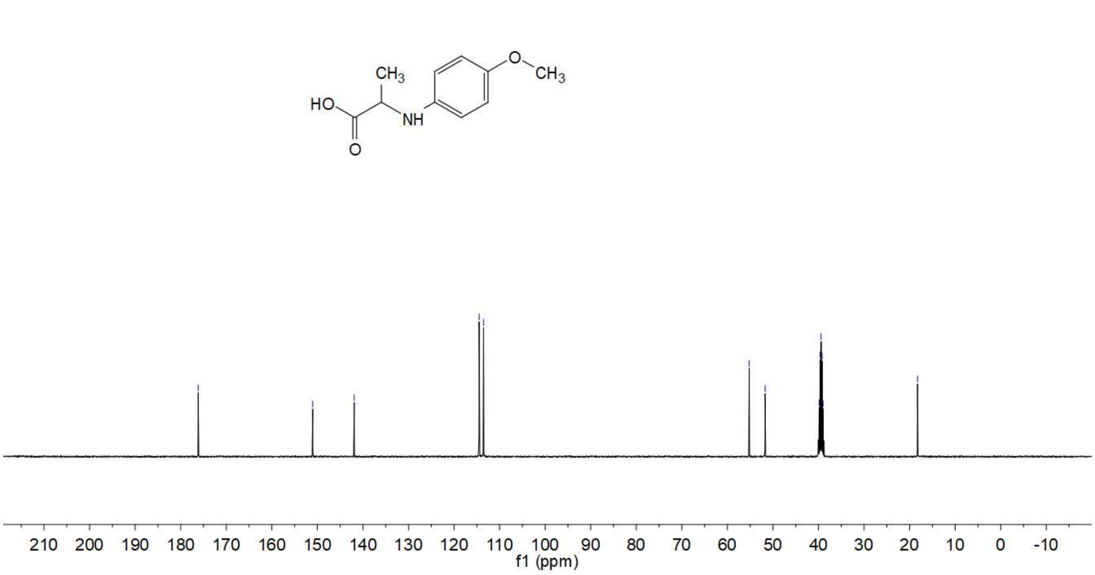

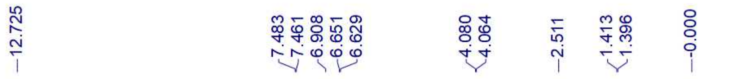

^{1}H NMR (400 MHz, DMSO) δ 12.73 (s, 1H), 7.47 (d, *J* = 8.9 Hz, 2H), 6.91 (s, 1H), 6.64 (d, *J* = 8.8 Hz, 2H), 4.07 (d, *J* = 6.7 Hz, 1H), 1.40 (d, *J* = 7.0 Hz, 3H).

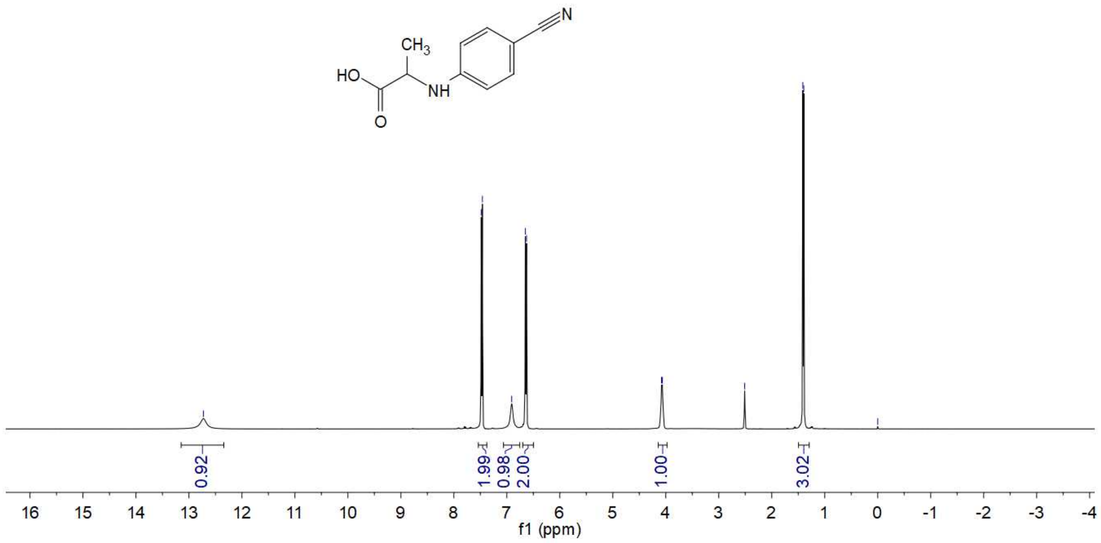

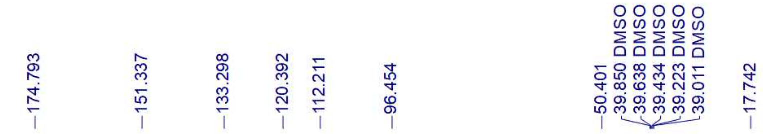

^{13}C NMR (101 MHz, DMSO) δ 174.79, 151.34, 133.30, 120.39, 112.21, 96.45, 50.40, 17.74.

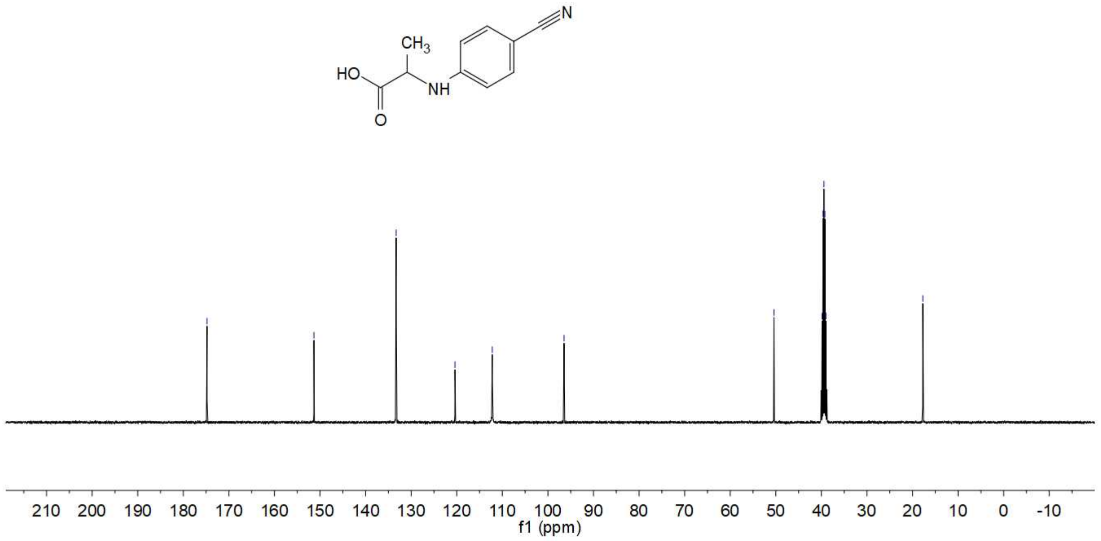

^{1}H NMR (400 MHz, DMSO) δ 12.83 (s, 1H), 8.01 (d, J = 9.0 Hz, 2H), 7.45 (d, J = 7.5 Hz, 1H), 6.65 (d, J = 9.0 Hz, 2H), 4.17 (p, J = 7.0 Hz, 1H), 1.43 (d, J = 7.0 Hz, 3H).

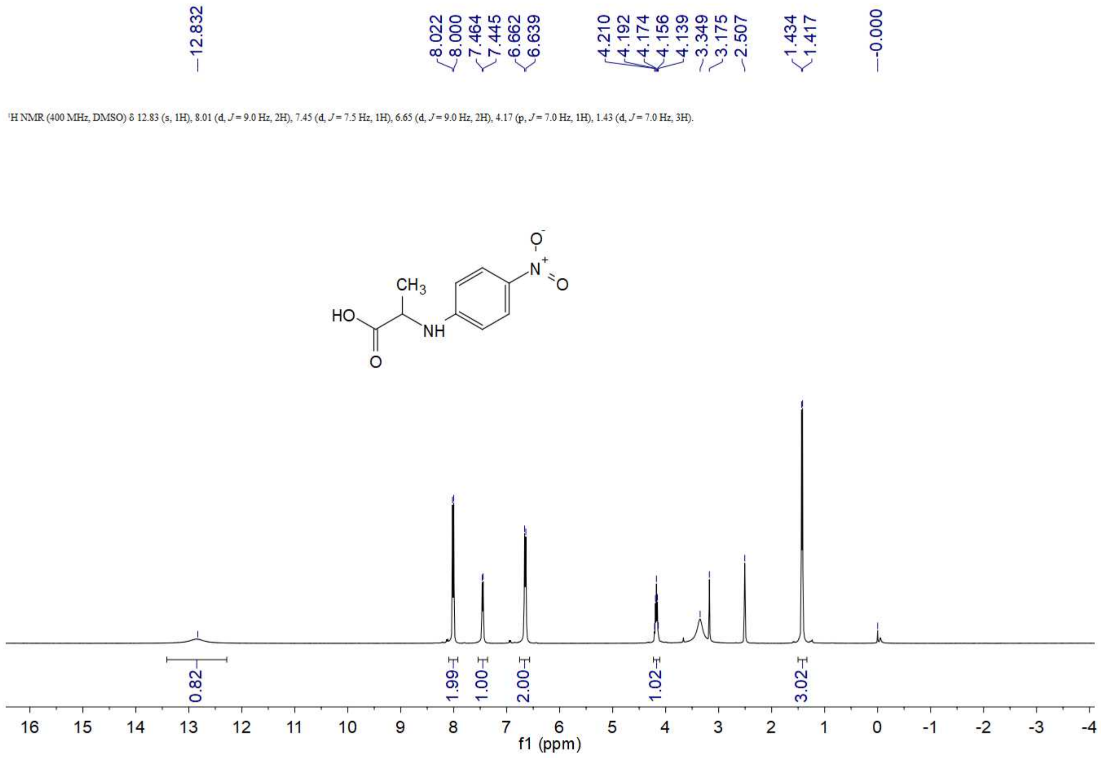

^{13}C NMR (101 MHz, DMSO) δ 174.35, 153.68, 136.25, 126.04, 111.24, 50.61, 17.68.

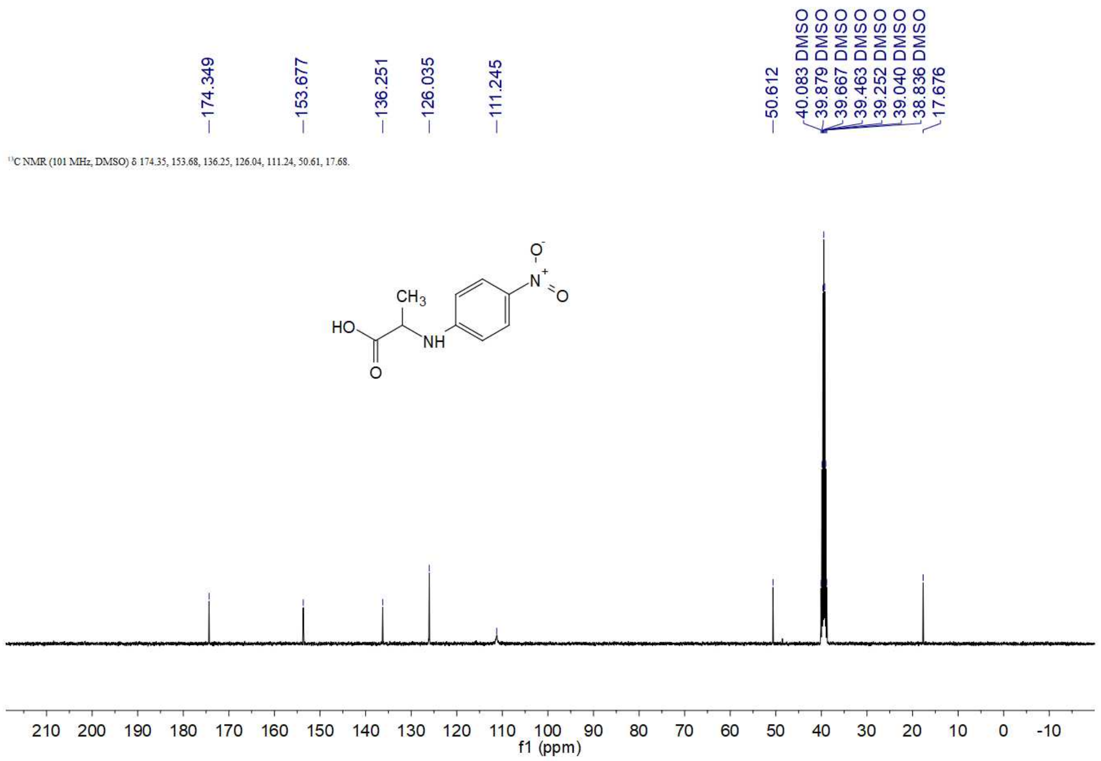

^{1}H NMR (400 MHz, DMSO) δ 12.69 (s, 1H), 7.39 (d, *J* = 8.5 Hz, 2H), 6.66 (d, *J* = 8.5 Hz, 2H), 6.62 (d, *J* = 5.0 Hz, 1H), 4.04 (s, 1H), 1.40 (d, *J* = 7.0 Hz, 3H).

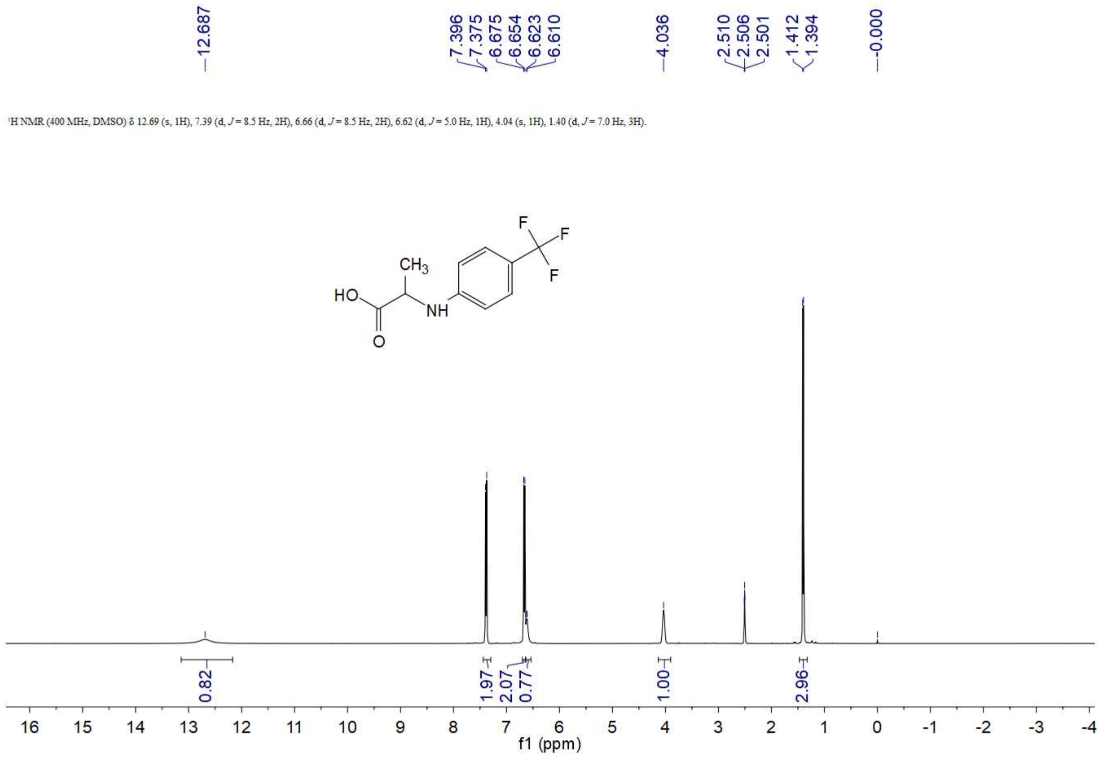

^{13}C NMR (101 MHz, DMSO) δ 175.15, 150.84, 129.26, 126.58, 126.18, 126.14, 126.11, 126.07, 123.90, 121.22, 116.30, 115.99, 115.67, 115.35, 111.69, 50.60, 17.85.

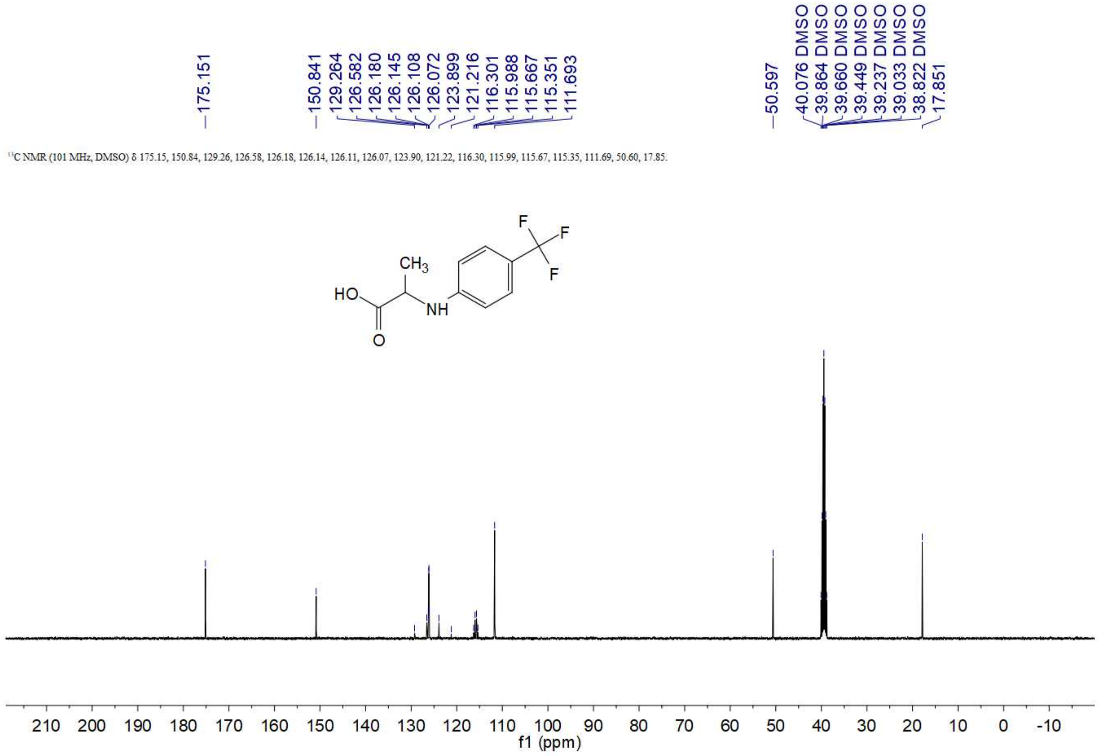

^{1}H NMR (400 MHz, DMSO) δ 12.69 (s, 1H), 7.64 (s, 1H), 7.48 (t, J = 8.7 Hz, 1H), 6.70 (s, 1H), 6.44 (d, J = 8.5 Hz, 1H), 6.31 (d, J = 14.6 Hz, 1H), 4.04 (s, 1H), 2.74 (d, J = 4.3 Hz, 3H), 1.38 (d, J = 6.9 Hz, 3H).

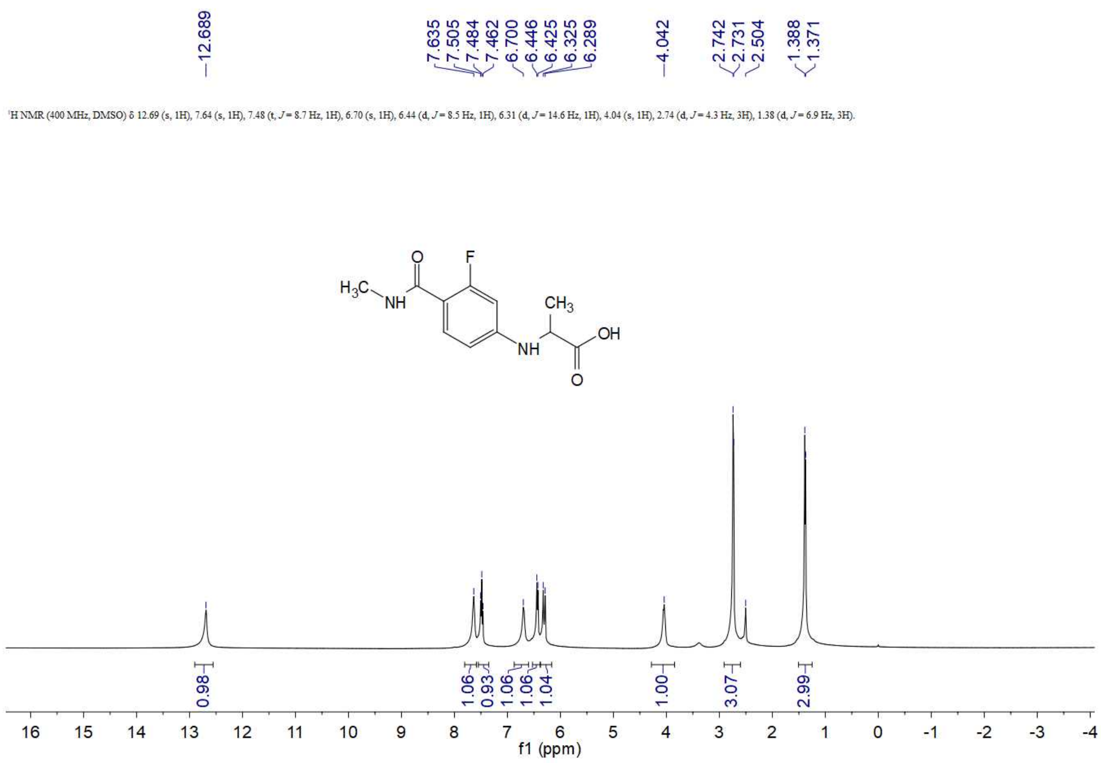

^{13}C NMR (101 MHz, DMSO) δ 174.97, 163.84, 162.37, 159.93, 151.86, 151.74, 131.40, 109.76, 109.62, 108.34, 98.11, 97.83, 50.65, 26.21, 17.79.

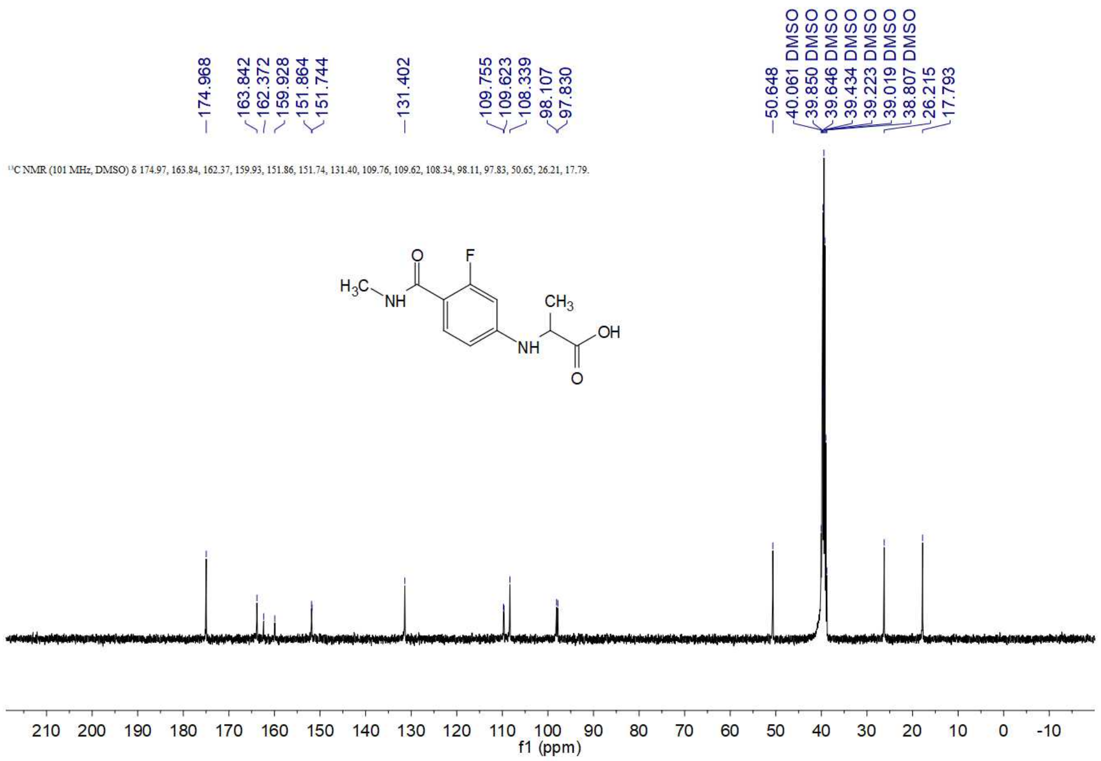

^{1}H and ^{13}C NMR spectra of *compound* **5a-l**

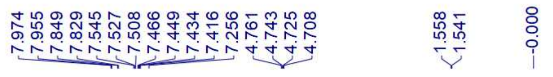

^{1}H NMR (400 MHz, $CDCl_3$) δ 7.96 (d, *J* = 7.8 Hz, 2H), 7.84 (d, *J* = 8.3 Hz, 1H), 7.53 (t, *J* = 7.5 Hz, 2H), 7.44 (dd, *J* = 12.9, 7.3 Hz, 3H), 4.73 (q, *J* = 7.0 Hz, 1H), 1.55 (d, *J* = 7.1 Hz, 3H).

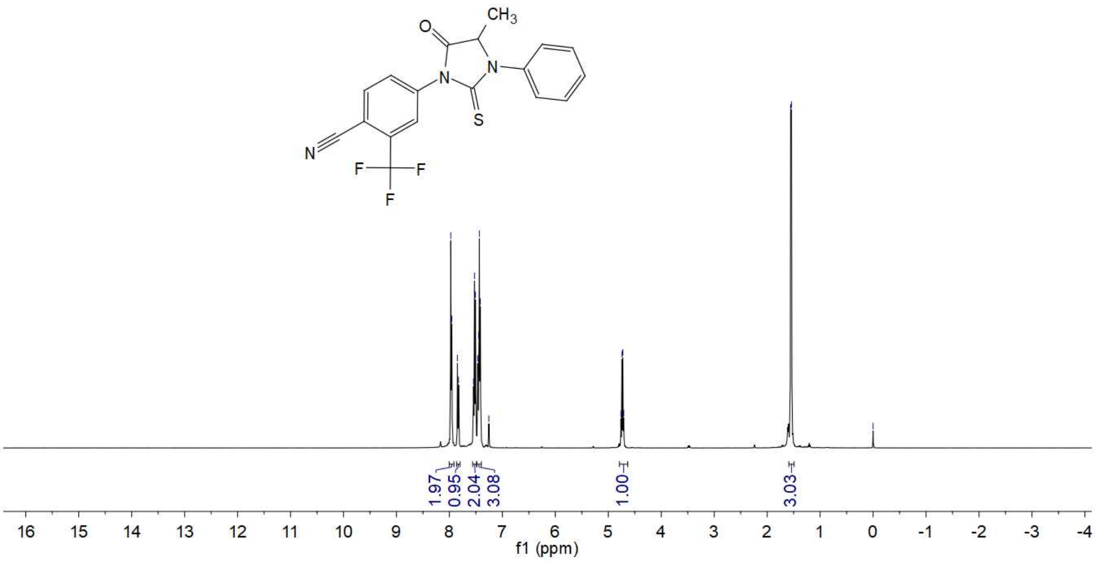

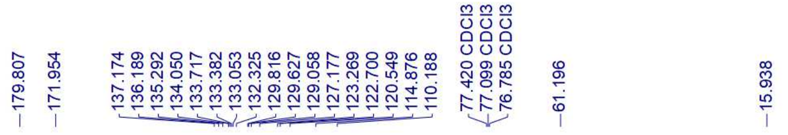

^{13}C NMR (101 MHz, $CDCl_3$) δ 179.81, 171.95, 137.17, 136.19, 135.29, 134.05, 133.72, 133.38, 133.05, 132.32, 129.82, 129.63, 129.06, 127.18, 123.27, 122.70, 120.55, 114.88, 110.19, 61.20, 15.94.

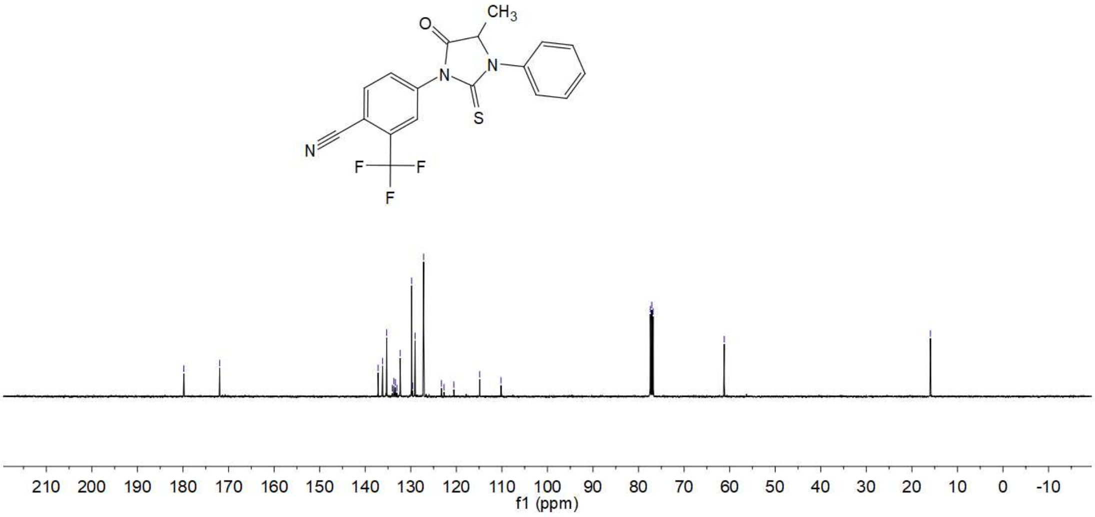

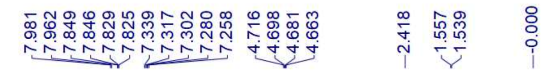

^{1}H NMR (400 MHz, $CDCl_3$) δ 7.97 (d, J = 7.5 Hz, 2H), 7.84 (dd, J = 8.3, 1.6 Hz, 1H), 7.33 (d, J = 8.5 Hz, 2H), 7.29 (d, J = 8.5 Hz, 2H), 4.69 (q, J = 7.0 Hz, 1H), 2.42 (s, 3H), 1.55 (d, J = 7.2 Hz, 3H).

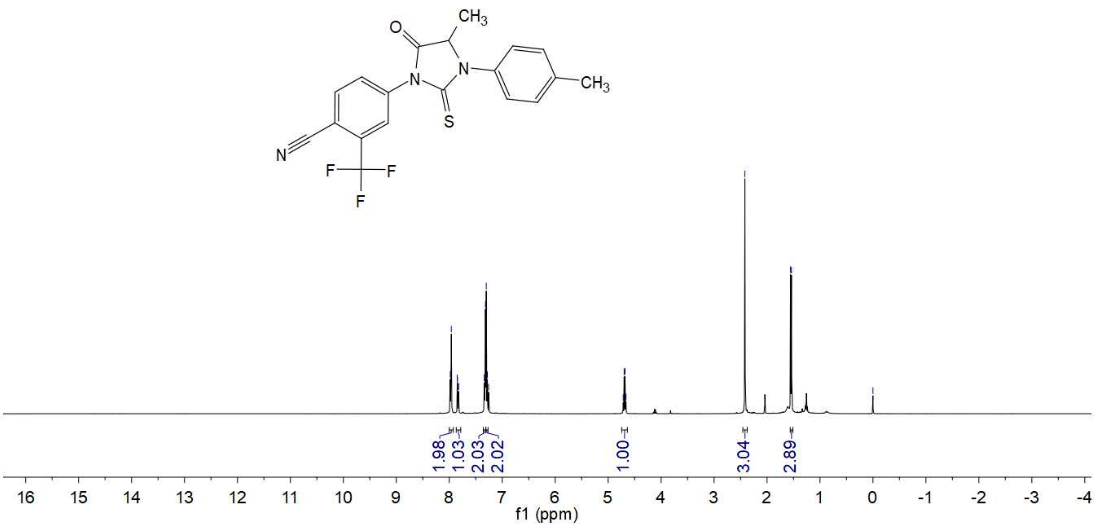

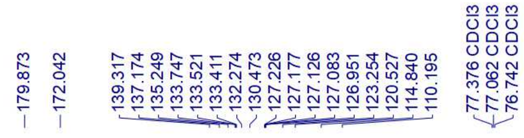

^{13}C NMR (101 MHz, $CDCl_3$) δ 179.87, 172.04, 139.32, 137.17, 135.25, 133.75, 133.52, 133.41, 132.27, 130.47, 127.23, 127.18, 127.13, 127.08, 126.95, 123.25, 120.53, 114.84, 110.20, 61.25, 21.25, 15.93.

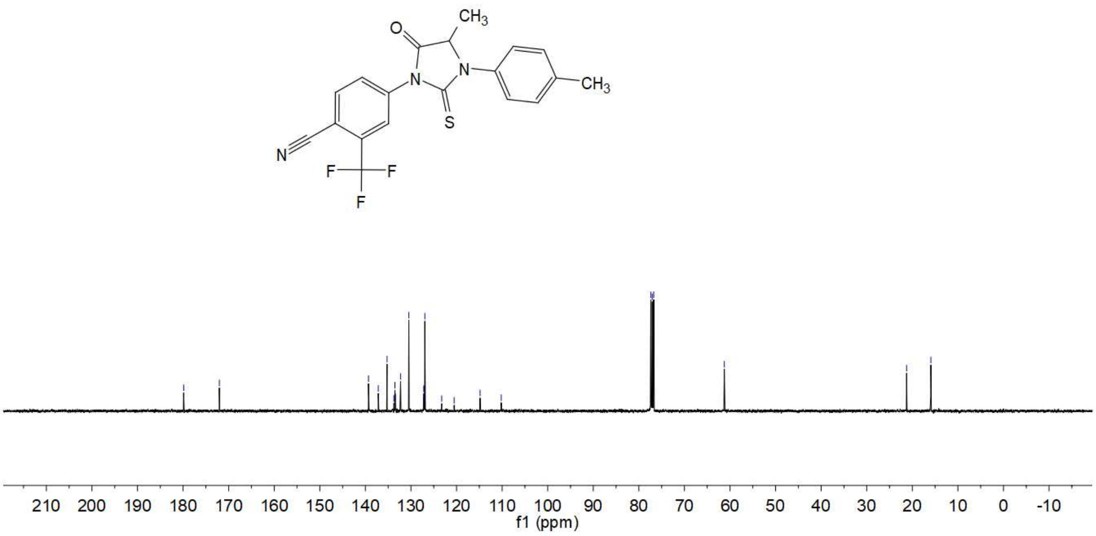

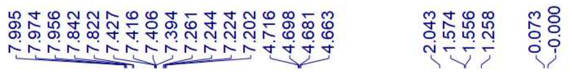

^{1}H NMR (400 MHz, $CDCl_3$) δ 8.04 – 7.92 (m, 2H), 7.83 (d, J = 8.0 Hz, 1H), 7.41 (dd, J = 8.7, 4.7 Hz, 2H), 7.22 (t, J = 8.4 Hz, 2H), 4.69 (q, J = 7.0 Hz, 1H), 1.57 (d, J = 7.2 Hz, 4H).

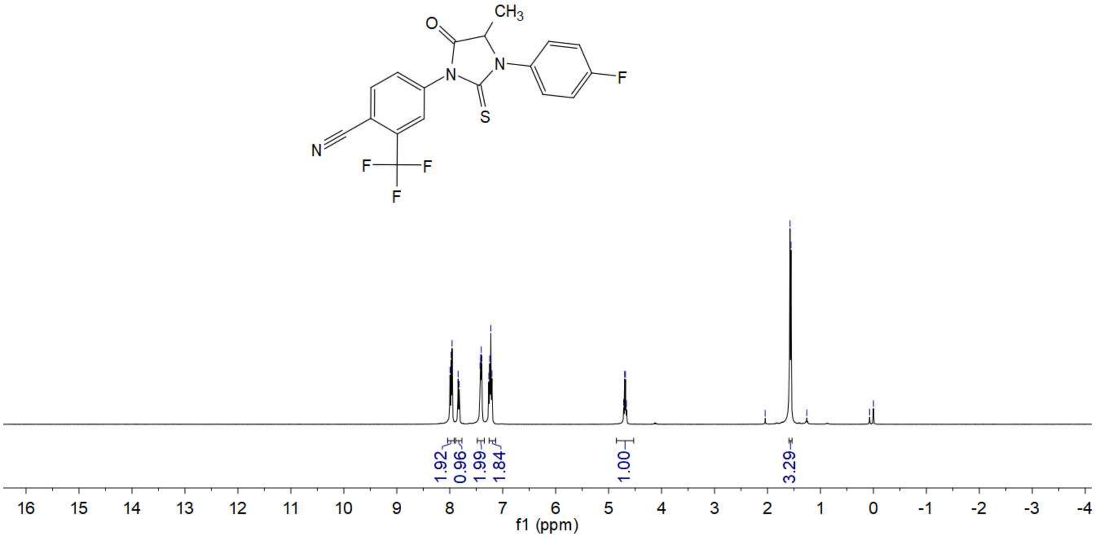

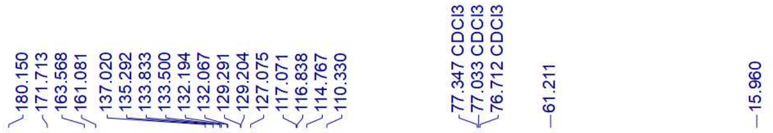

^{13}C NMR (101 MHz, $CDCl_3$) δ 180.15, 171.71, 163.57, 161.08, 137.02, 135.29, 133.83, 133.50, 132.19, 132.07, 129.29, 129.20, 127.07, 117.07, 116.84, 114.77, 110.33, 61.21, 15.96.

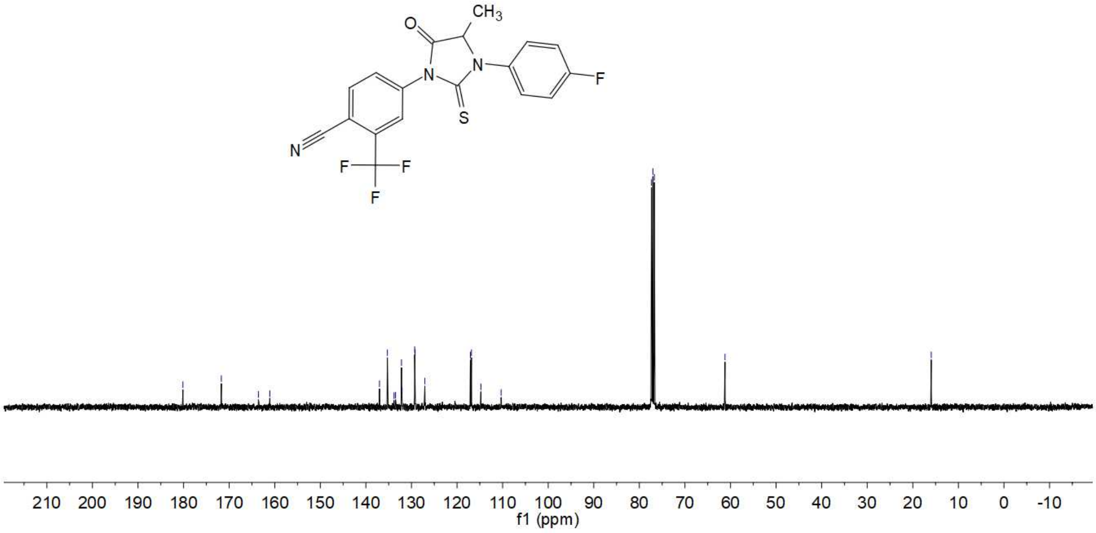

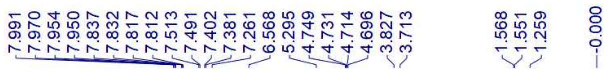

^{1}H NMR (400 MHz, $CDCl_3$) δ 7.98 (d, *J* = 8.5 Hz, 1H), 7.95 (d, *J* = 1.6 Hz, 1H), 7.82 (dd, *J* = 8.3, 2.0 Hz, 1H), 7.50 (d, *J* = 8.7 Hz, 2H), 7.39 (d, *J* = 8.7 Hz, 2H), 4.72 (q, *J* = 7.1 Hz, 1H), 1.56 (d, *J* = 7.0 Hz, 3H).

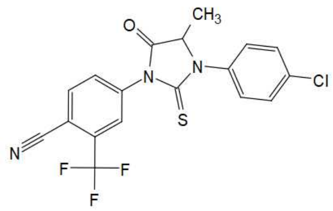

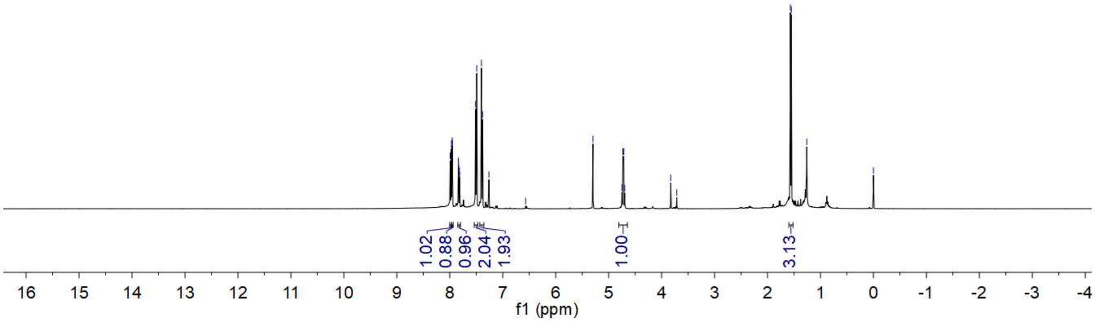

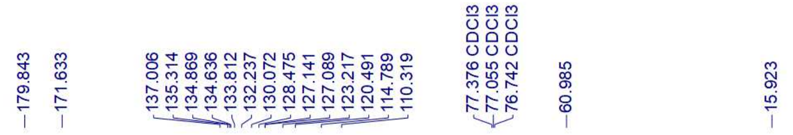

^{13}C NMR (101 MHz, $CDCl_3$) δ 179.84, 171.63, 137.01, 135.31, 134.87, 134.64, 133.81, 132.24, 130.07, 128.47, 127.14, 127.09, 123.22, 120.49, 114.79, 110.32, 60.98, 15.92.

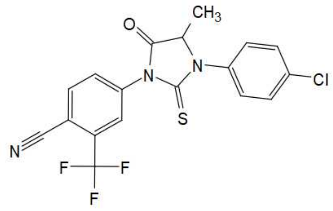

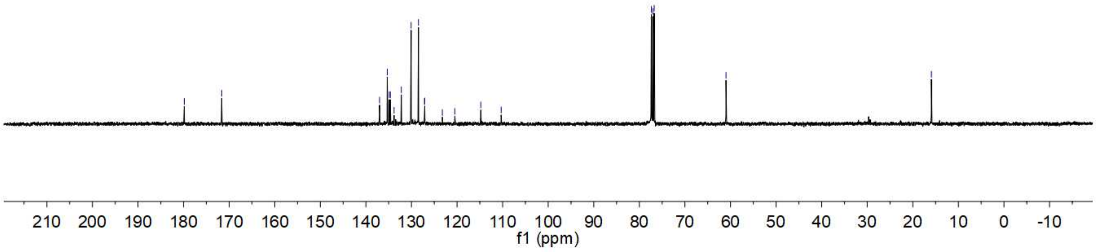

^{1}H NMR (400 MHz, $CDCl_3$) δ 7.98 (d, J = 8.3 Hz, 1H), 7.95 (d, J = 1.3 Hz, 1H), 7.82 (dd, J = 8.3, 1.9 Hz, 1H), 7.66 (d, J = 8.3 Hz, 2H), 7.33 (d, J = 8.2 Hz, 2H), 4.82 – 4.65 (m, 1H), 1.56 (d, J = 7.0 Hz, 3H).

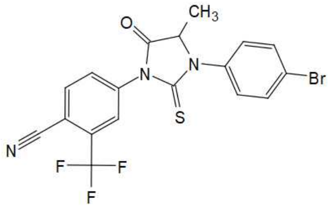

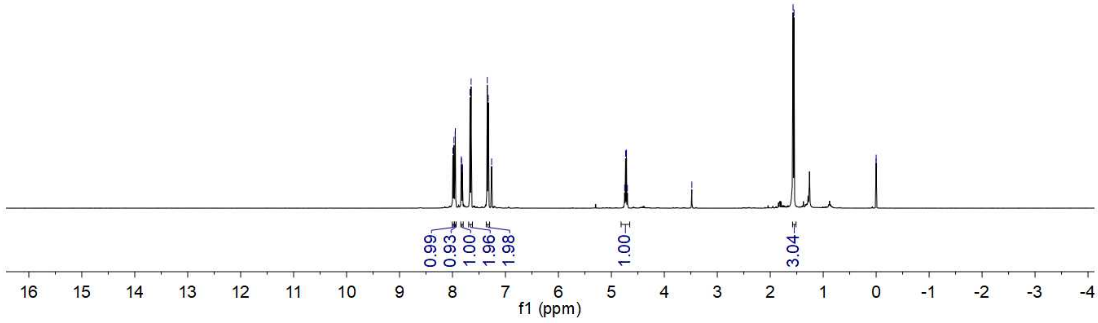

^{13}C NMR (101 MHz, $CDCl_3$) δ 179.74, 171.60, 136.97, 135.31, 135.16, 133.84, 133.51, 133.05, 132.22, 128.71, 127.18, 127.13, 127.09, 127.04, 123.21, 122.87, 120.48, 114.77, 110.36, 60.90, 15.94.

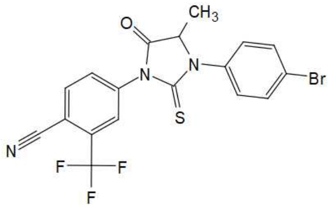

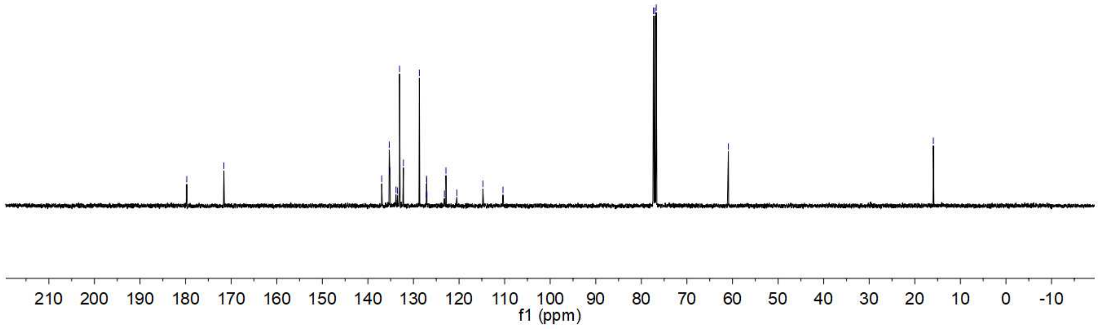

^{1}H NMR (400 MHz, $CDCl_3$) δ 7.97 (d, J = 8.2 Hz, 2H), 7.84 (d, J = 9.4 Hz, 1H), 7.32 (d, J = 8.9 Hz, 2H), 7.02 (d, J = 8.9 Hz, 2H), 4.65 (q, J = 7.0 Hz, 1H), 3.86 (s, 3H), 1.55 (d, J = 7.0 Hz, 3H).

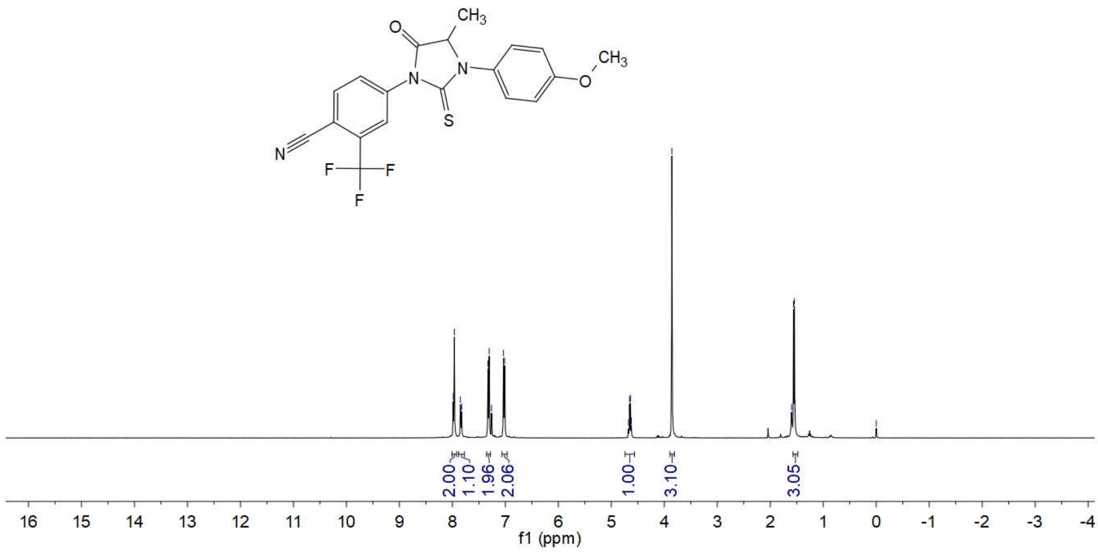

^{13}C NMR (101 MHz, $CDCl_3$) δ 180.14, 172.04, 159.81, 137.18, 135.24, 133.75, 133.42, 132.24, 128.66, 128.53, 127.24, 127.16, 127.10, 127.06, 123.25, 120.52, 115.05, 114.83, 110.19, 61.40, 55.56, 15.95.

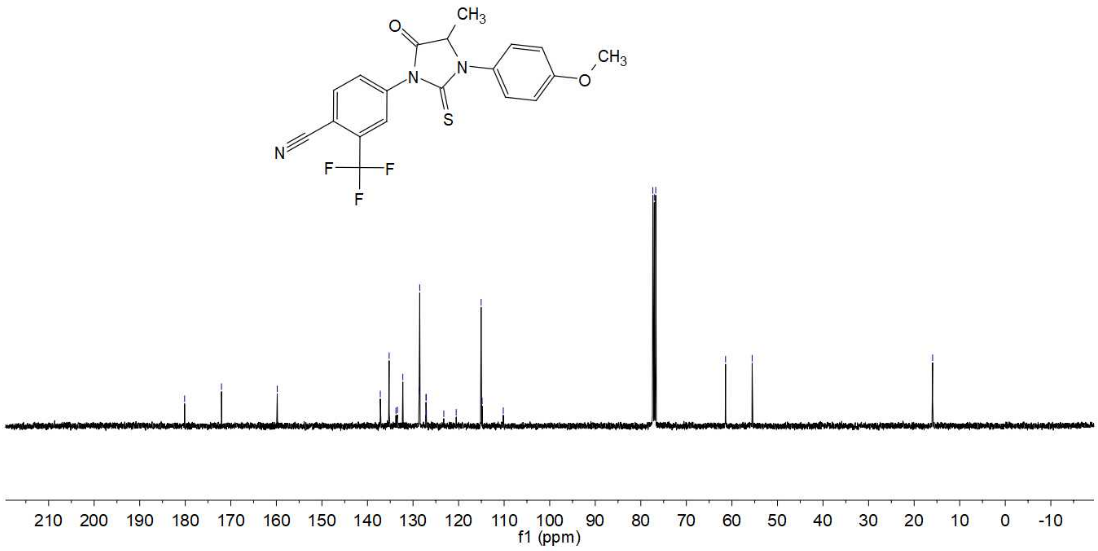

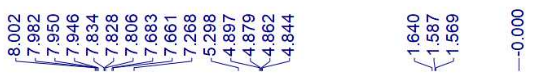

^{1}H NMR (400 MHz, $CDCl_3$) δ 7.99 (d, *J* = 8.3 Hz, 1H), 7.95 (d, *J* = 1.6 Hz, 1H), 7.86 – 7.77 (m, 3H), 7.67 (d, *J* = 8.7 Hz, 2H), 4.87 (q, *J* = 7.0 Hz, 1H), 1.58 (d, *J* = 7.0 Hz, 3H).

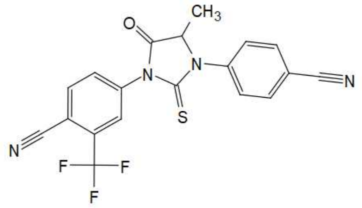

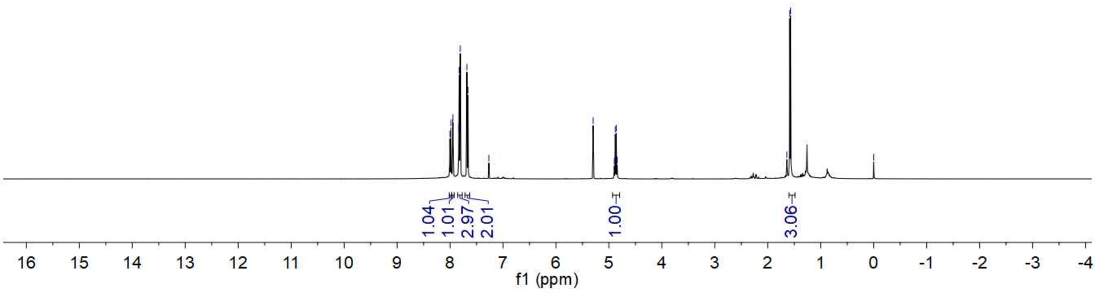

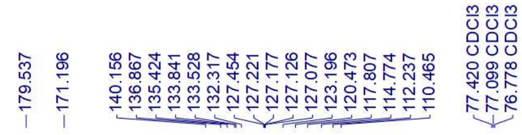

^{13}C NMR (101 MHz, $CDCl_3$) δ 179.54, 171.20, 140.16, 136.87, 135.42, 133.84, 133.53, 132.32, 127.45, 127.22, 127.18, 127.13, 127.08, 123.20, 120.47, 117.81, 114.77, 112.24, 110.46, 60.42, 15.87.

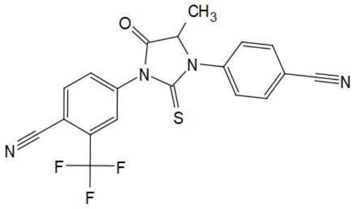

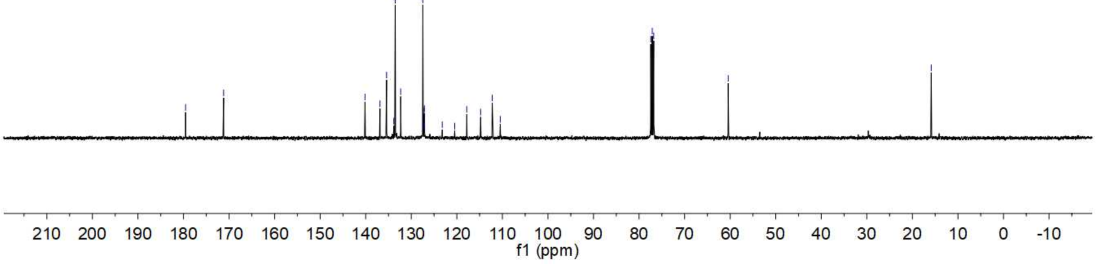

^{1}H NMR (400 MHz, $CDCl_3$) δ 8.39 (d, J = 9.0 Hz, 2H), 8.01 (d, J = 8.3 Hz, 1H), 7.95 (d, J = 1.4 Hz, 1H), 7.83 (dd, J = 8.3, 1.9 Hz, 1H), 7.75 (d, J = 9.0 Hz, 2H), 4.92 (q, J = 7.0 Hz, 1H), 1.60 (d, J = 7.0 Hz, 3H).

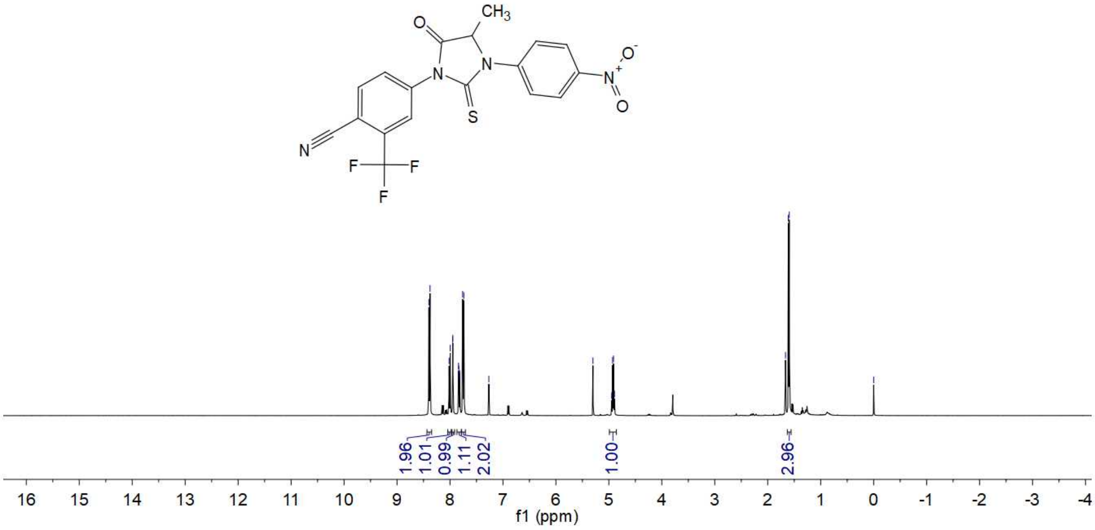

^{13}C NMR (101 MHz, $CDCl_3$) δ 179.52, 171.09, 146.84, 141.72, 136.79, 135.45, 133.94, 133.60, 132.29, 127.38, 127.22, 127.17, 127.12, 127.08, 126.19, 125.01, 123.17, 120.45, 115.65, 114.72, 110.57, 60.43, 15.92.

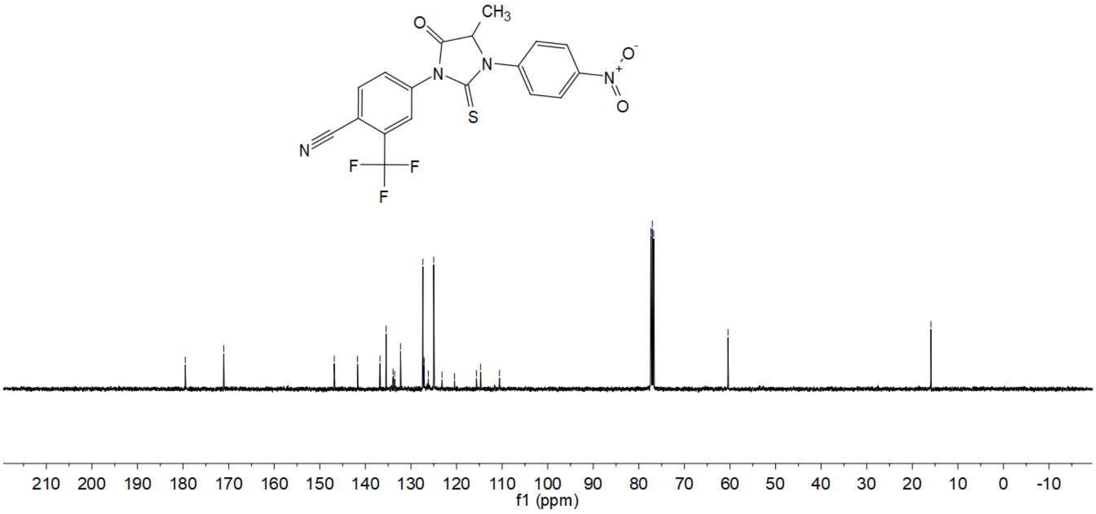

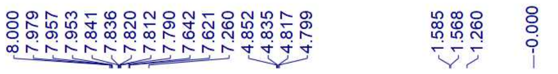

^{1}H NMR (400 MHz, $CDCl_3$) δ 7.99 (d, *J* = 8.3 Hz, 1H), 7.96 (d, *J* = 1.6 Hz, 1H), 7.86 – 7.76 (m, 3H), 7.63 (d, *J* = 8.3 Hz, 2H), 4.83 (q, *J* = 7.1 Hz, 1H), 1.58 (d, *J* = 7.0 Hz, 3H).

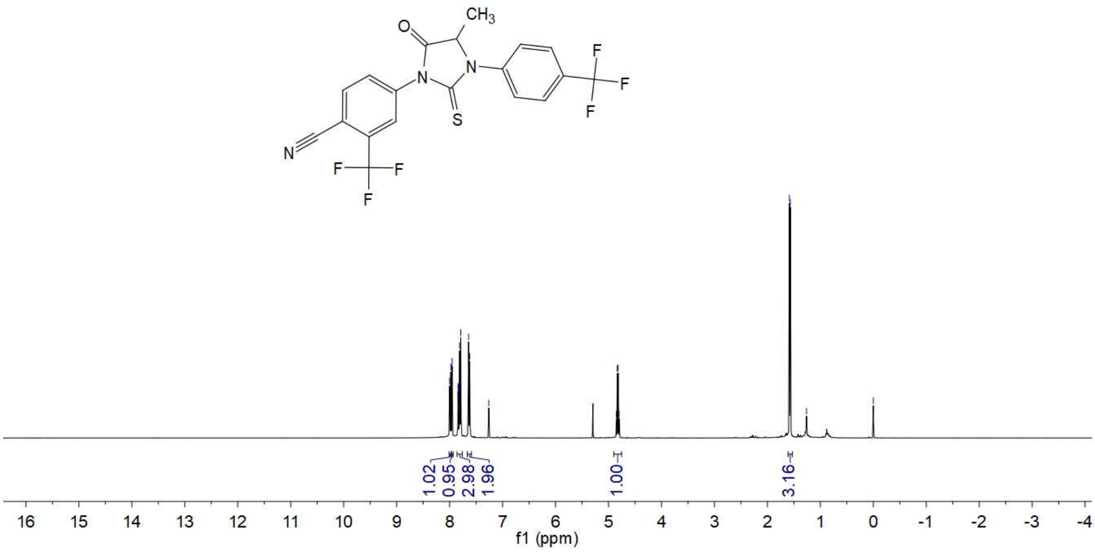

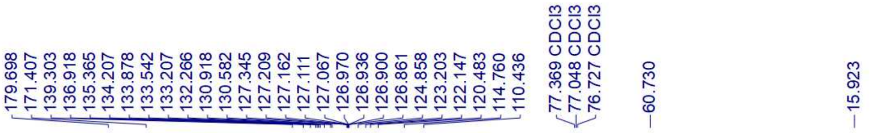

^{13}C NMR (101 MHz, $CDCl_3$) δ 179.70, 171.41, 139.30, 136.92, 135.37, 134.21, 133.88, 133.54, 133.21, 132.27, 130.92, 130.58, 127.34, 127.21, 127.16, 127.11, 127.07, 126.97, 126.94, 126.90, 126.86, 124.86, 123.20, 122.15, 120.48, 114.76, 110.44, 60.73, 15.92

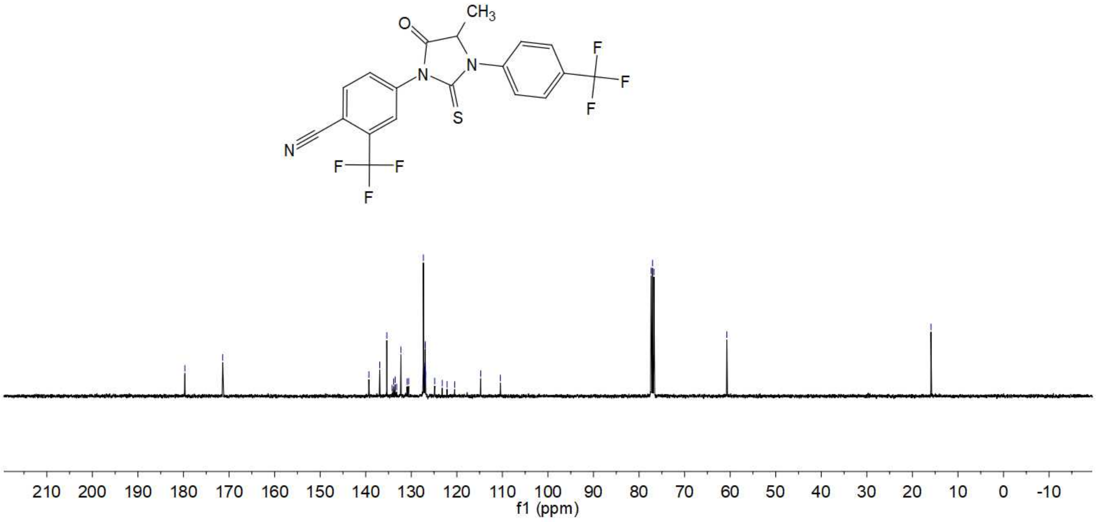

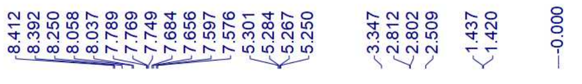

^{1}H NMR (400 MHz, DMSO) δ 8.41 (s, 1H), 8.39 (s, 1H), 8.25 (s, 1H), 8.05 (d, *J* = 8.2 Hz, 1H), 7.77 (t, *J* = 8.0 Hz, 1H), 7.67 (d, *J* = 11.4 Hz, 1H), 7.59 (d, *J* = 8.3 Hz, 1H), 5.28 (q, *J* = 6.7 Hz, 1H), 2.81 (d, *J* = 3.8 Hz, 3H), 1.43 (d, *J* = 6.8 Hz, 3H).

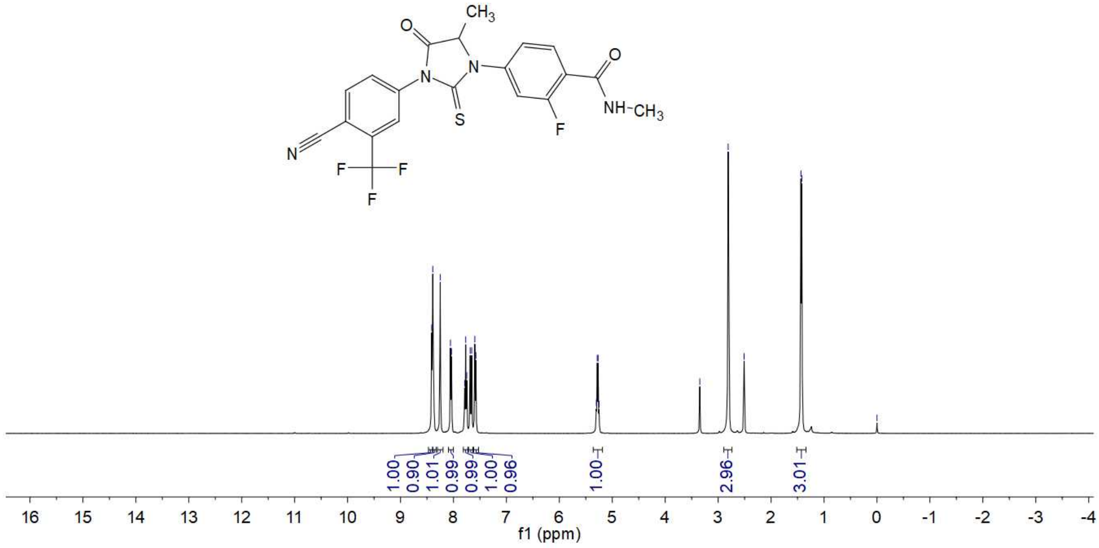

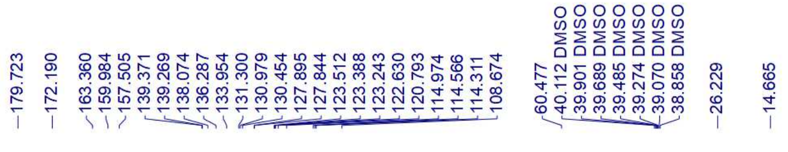

^{13}C NMR (101 MHz, DMSO) δ 179.72, 172.19, 163.36, 159.98, 157.51, 139.37, 139.27, 138.07, 136.29, 133.95, 131.30, 130.98, 130.45, 127.89, 127.84, 123.51, 123.39, 123.24, 122.63, 120.79, 114.97, 114.57, 114.31, 108.67, 60.48, 26.23, 14.66.

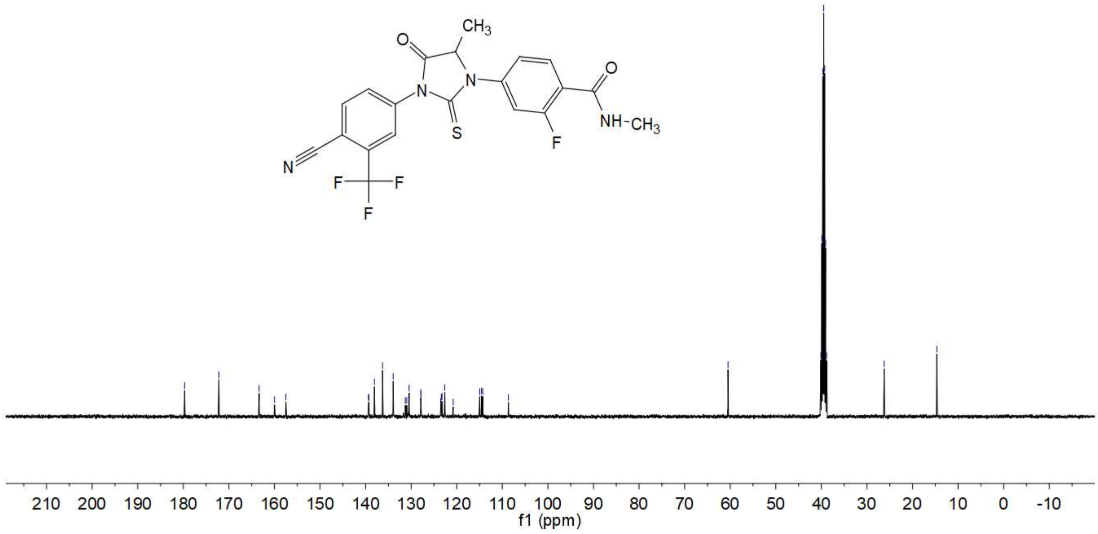

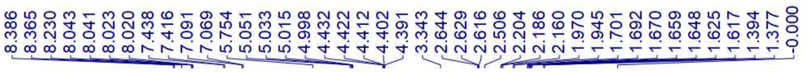

^{1}H NMR (400 MHz, DMSO) δ 8.38 (d, J = 8.3 Hz, 1H), 8.23 (s, 1H), 8.03 (dd, J = 8.2, 1.0 Hz, 1H), 7.43 (d, J = 8.8 Hz, 2H), 7.08 (d, J = 8.9 Hz, 2H), 5.02 (q, J = 7.0 Hz, 1H), 4.49 – 4.33 (m, 1H), 2.67 – 2.58 (m, 2H), 2.22 – 2.14 (m, 5H), 1.96 (d, J = 10.2 Hz, 2H), 1.71 – 1.60 (m, 2H), 1.39 (d, J = 7.0 Hz, 3H).

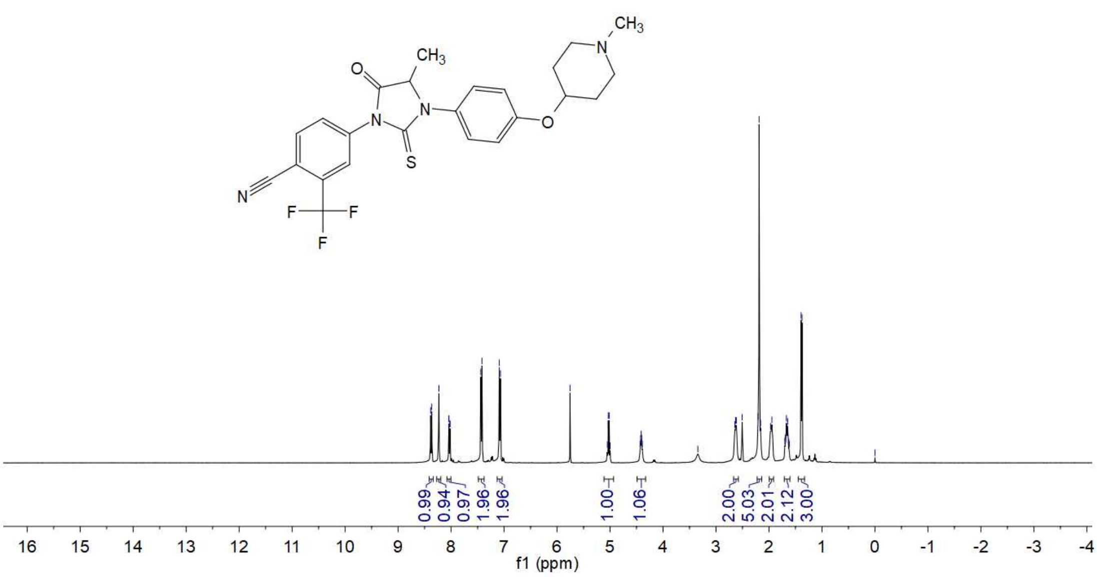

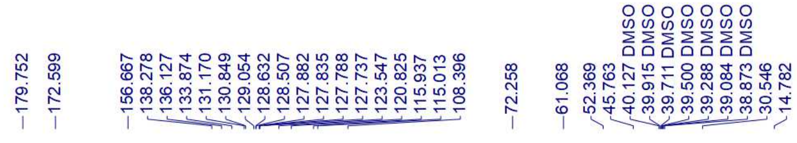

^{13}C NMR (101 MHz, DMSO) δ 179.75, 172.60, 156.67, 138.28, 136.13, 133.87, 131.17, 130.85, 129.05, 128.63, 128.51, 127.88, 127.84, 127.79, 127.74, 123.55, 120.82, 115.94, 115.01, 108.40, 72.26, 61.07, 52.37, 45.76, 30.55, 14.78.

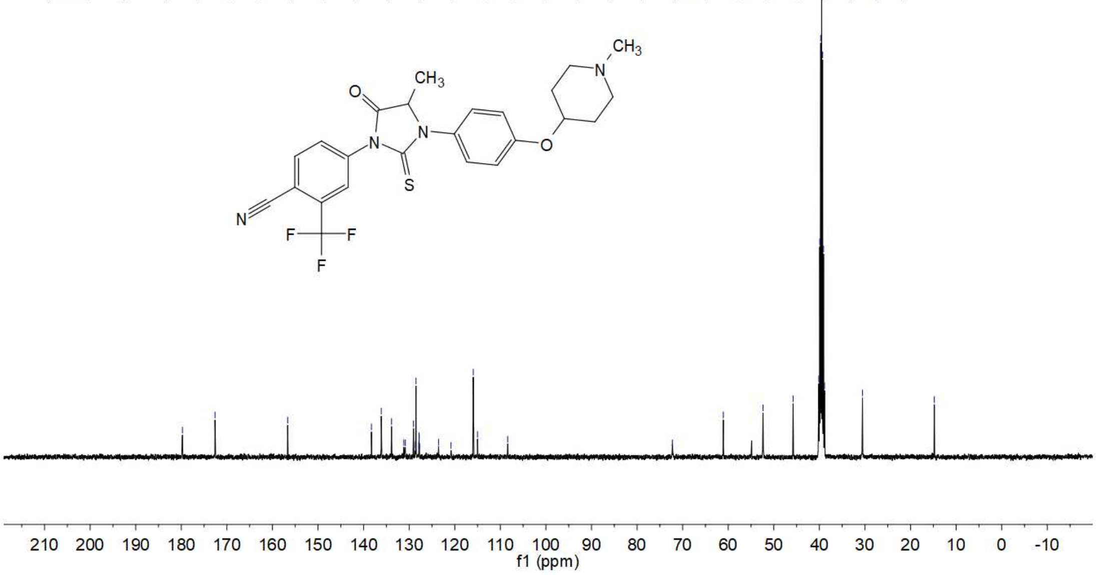

^{1}H and ^{13}C NMR spectra of *compound* **13-17**

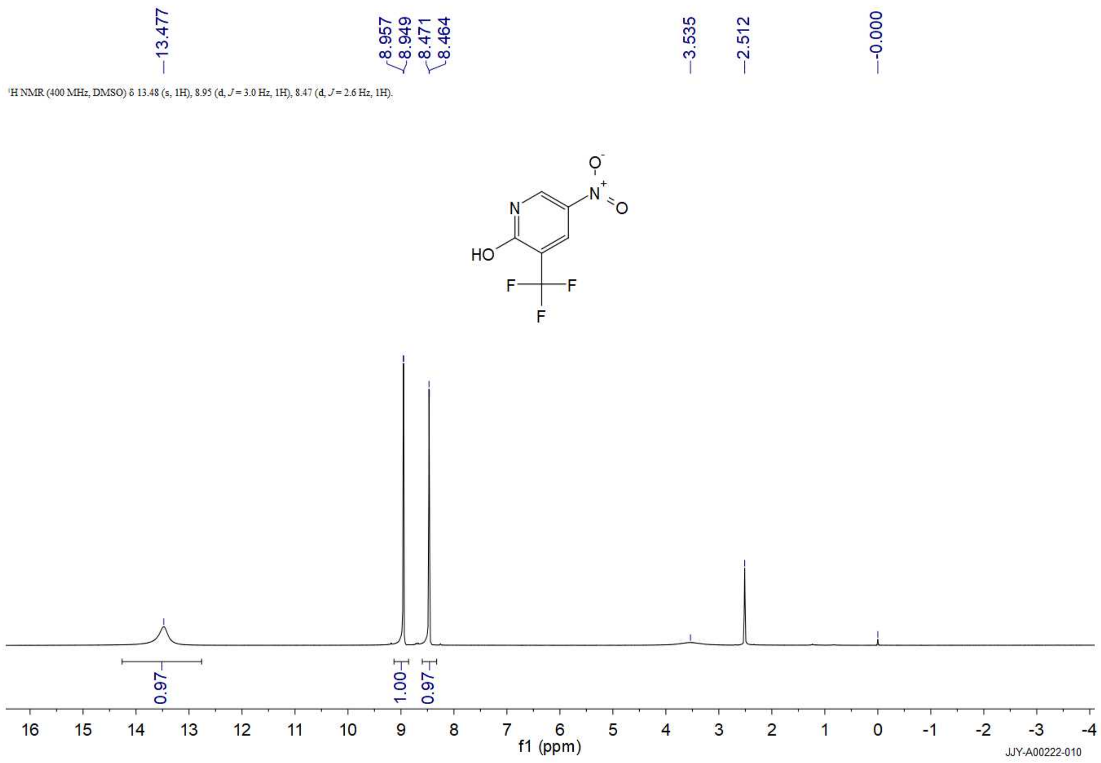

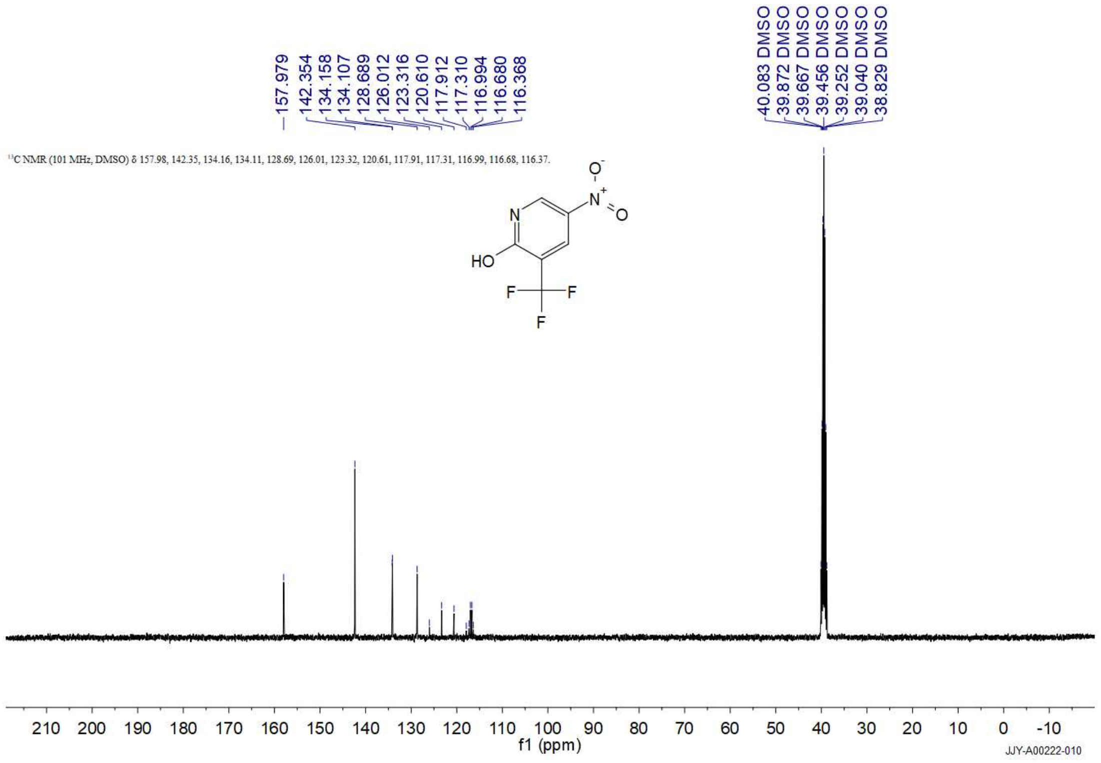

^{1}H NMR (400 MHz, $CDCl_3$) δ 9.36 (d, *J* = 1.2 Hz, 1H), 8.76 (d, *J* = 1.6 Hz, 1H).

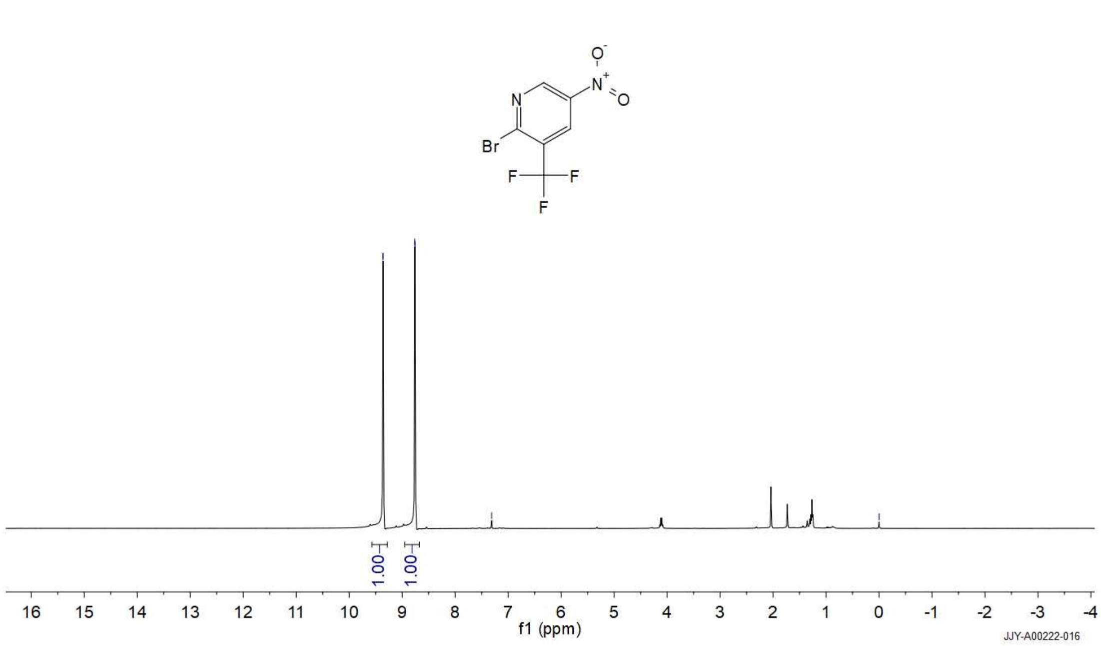

^{13}C NMR (101 MHz, $CDCl_3$) δ 147.29, 145.58, 142.82, 131.60, 131.54, 131.49, 131.44, 129.28, 128.93, 128.58, 128.24, 125.24, 122.52, 119.80, 117.08.

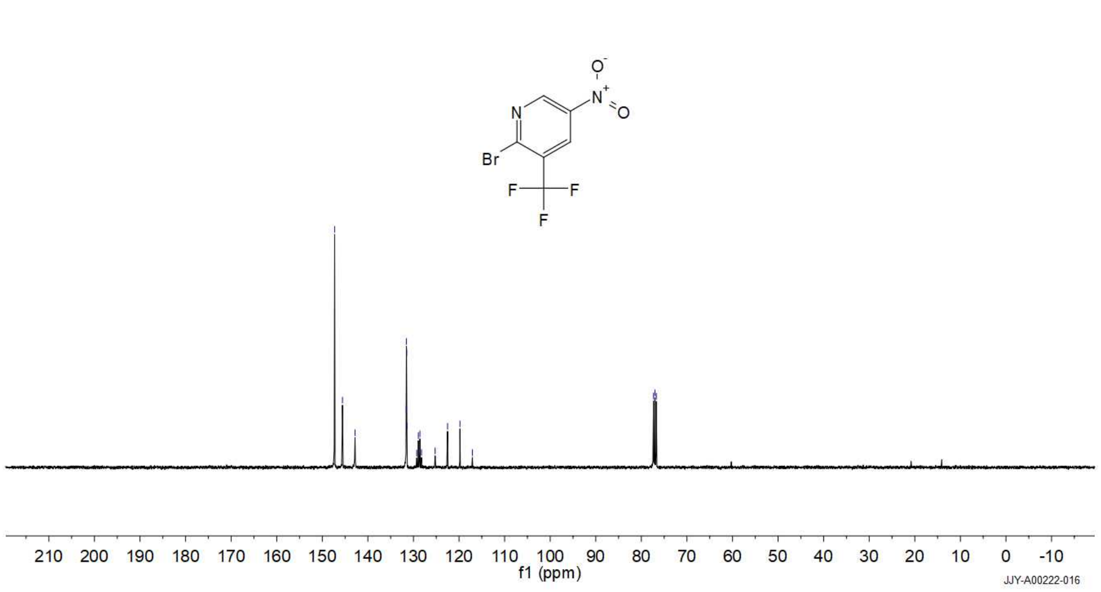

^{1}H NMR (400 MHz, $CDCl_3$) δ 7.97 (s, 1H), 7.26 (s, 1H), 3.81 (s, 2H).

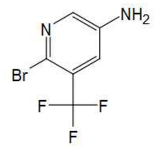

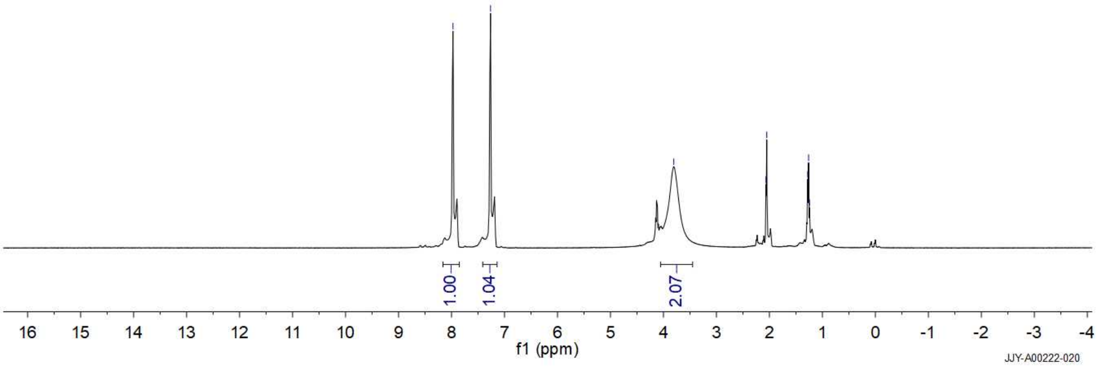

^{13}C NMR (101 MHz, $CDCl_3$) δ 141.94, 138.79, 127.51, 127.19, 125.38, 123.56, 121.82, 121.77, 121.72, 120.85.

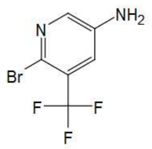

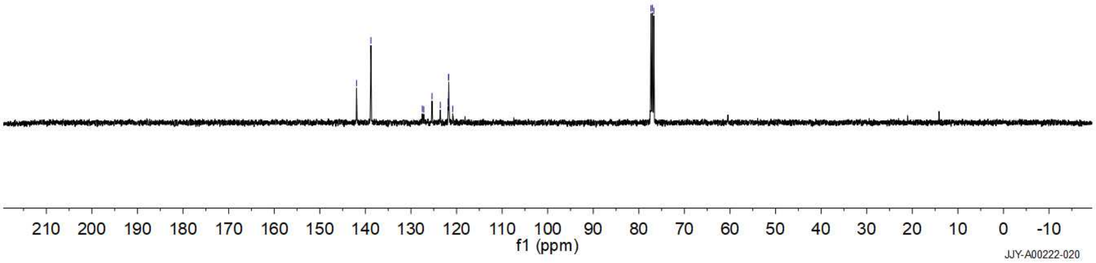

^{1}H NMR (400 MHz, DMSO) δ 8.17 (d, J = 1.7 Hz, 1H), 7.27 (d, J = 1.9 Hz, 1H), 6.97 (s, 2H).

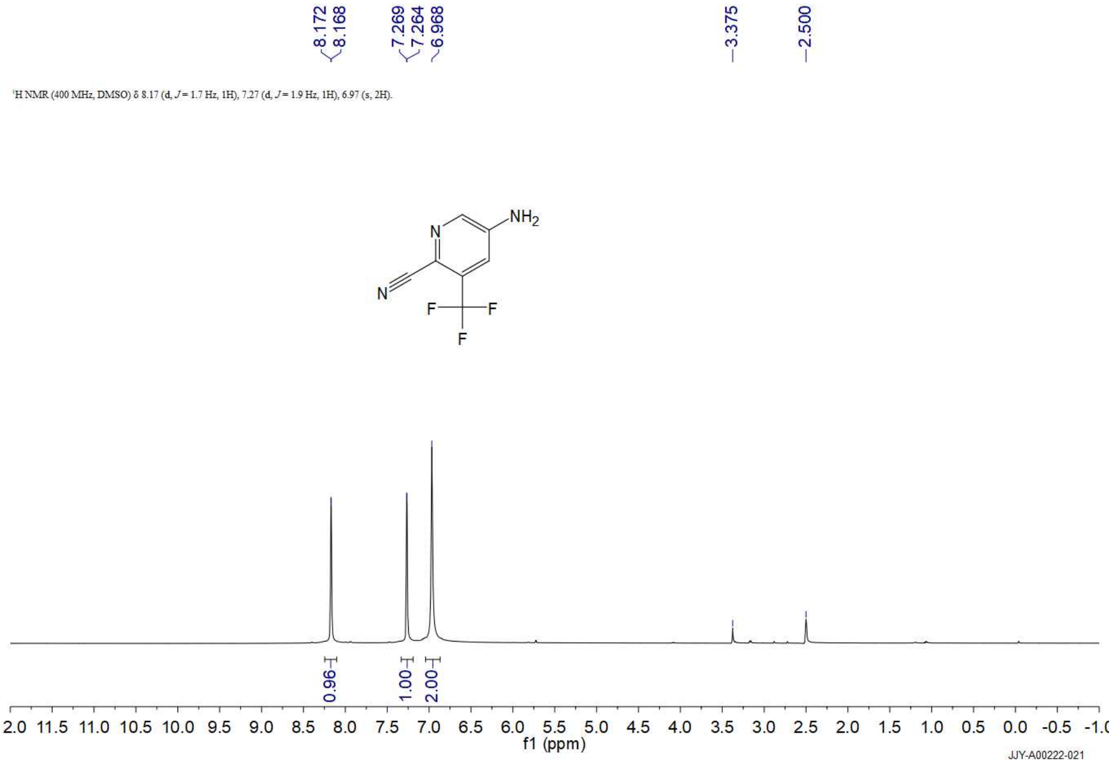

^{13}C NMR (101 MHz, DMSO) δ 147.96, 139.37, 130.34, 130.02, 129.70, 129.38, 126.28, 123.56, 120.84, 118.12, 116.01, 115.07, 115.02, 114.97, 114.93, 112.92.

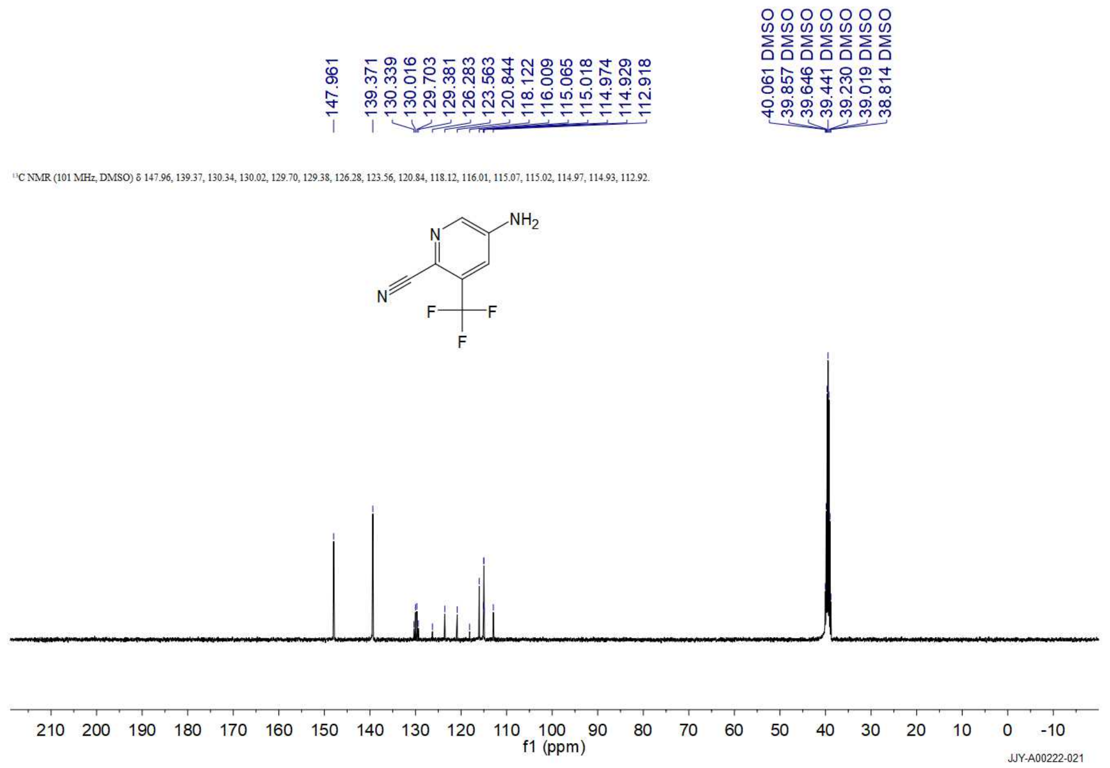

^{1}H NMR (400 MHz, $CDCl_3$) δ 8.70 (s, 1H), 7.86 (s, 1H).

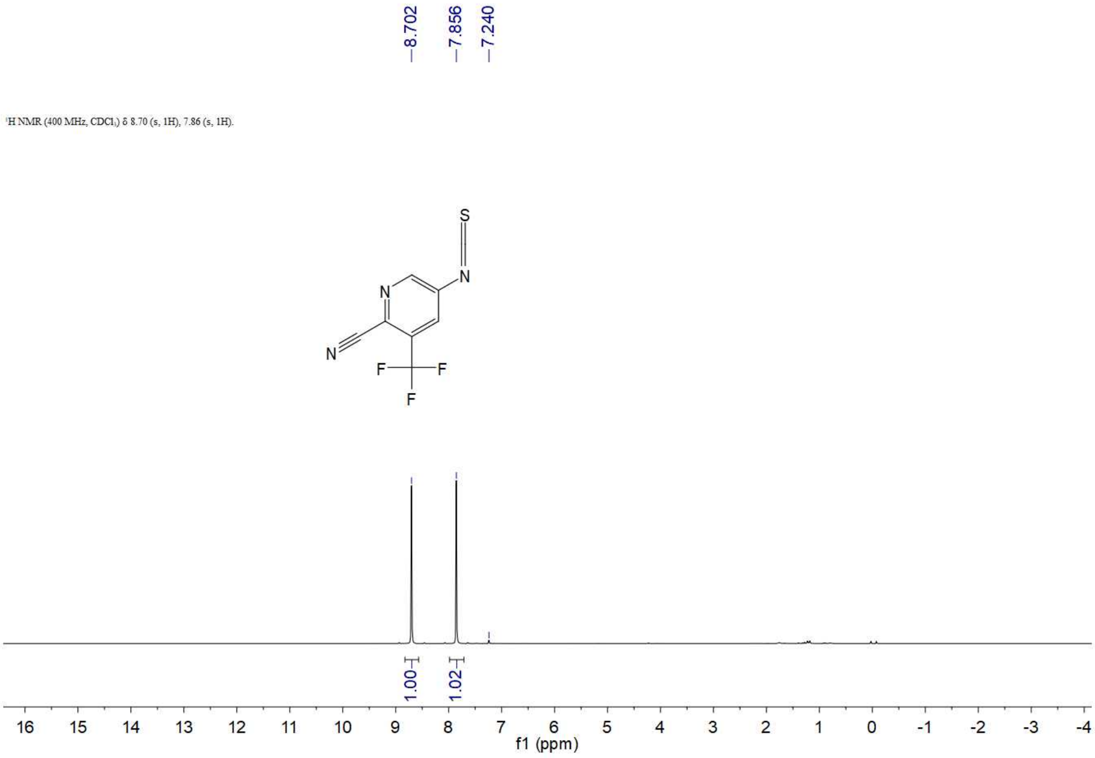

^{13}C NMR (101 MHz, $CDCl_3$) δ 150.39, 144.46, 133.53, 131.83, 131.49, 131.14, 130.80, 130.60, 130.56, 130.52, 130.47, 127.27, 127.25, 125.15, 122.42, 119.69, 116.95, 113.67.

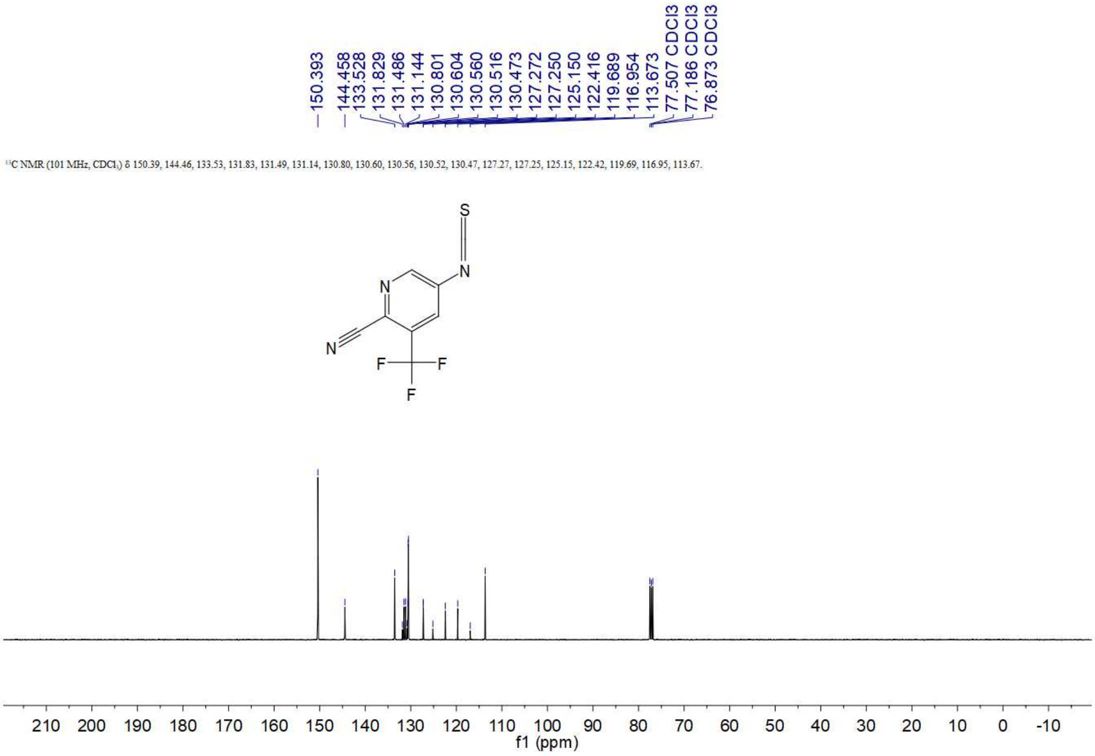

^{1}H and ^{13}C NMR spectra of *compound* **18a-d**

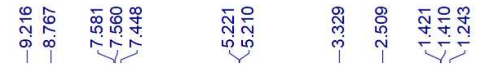

^{1}H NMR (400 MHz, DMSO) δ 9.22 (s, 1H), 8.77 (s, 1H), 7.57 (d, *J* = 8.4 Hz, 4H), 7.45 (s, 1H), 5.22 (d, *J* = 4.3 Hz, 1H), 1.42 (d, *J* = 4.1 Hz, 3H).

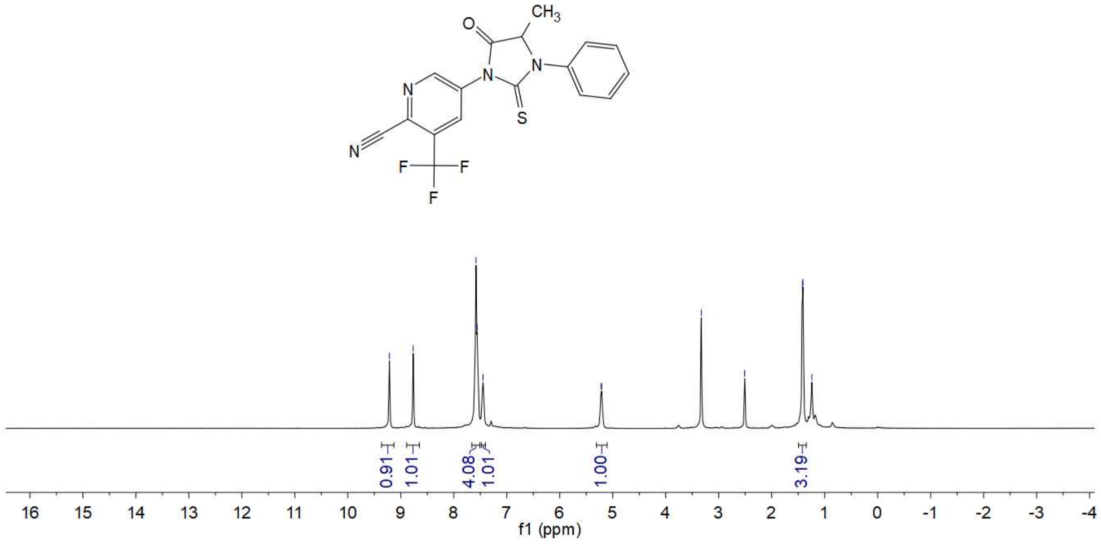

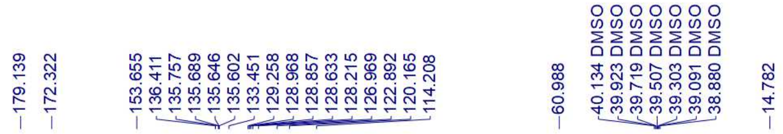

^{13}C NMR (101 MHz, DMSO) δ 179.14, 172.32, 153.66, 136.41, 135.76, 135.69, 135.65, 135.60, 133.45, 129.26, 128.97, 128.86, 128.63, 128.22, 126.97, 122.89, 120.17, 114.21, 60.99, 14.78.

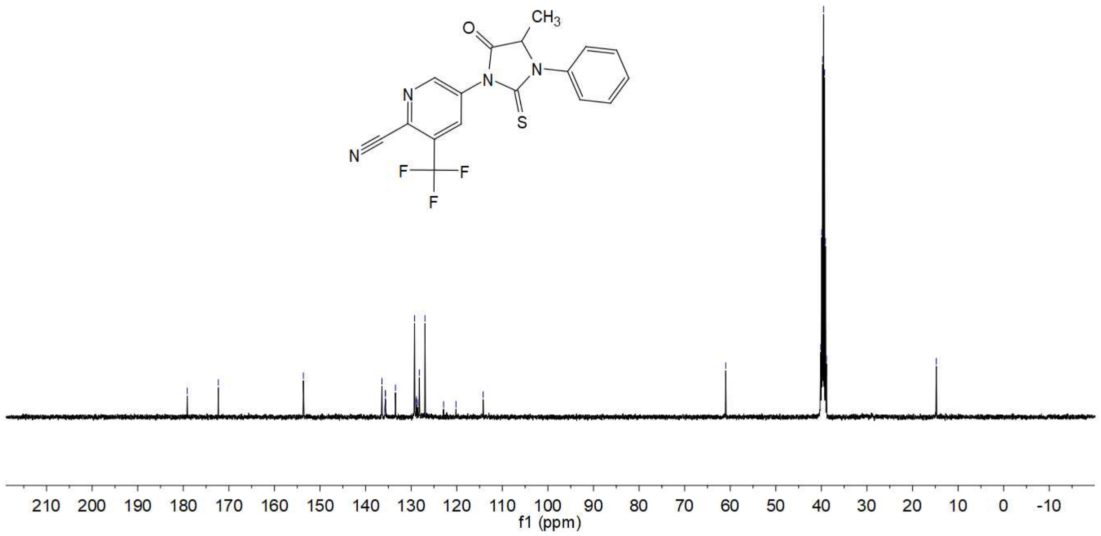

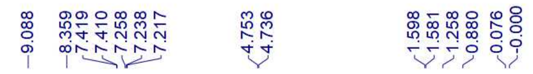

^{1}H NMR (400 MHz, $CDCl_3$) δ 9.09 (s, 1H), 8.36 (s, 1H), 7.41 (d, J = 3.4 Hz, 2H), 7.24 (t, J = 8.2 Hz, 2H), 4.74 (d, J = 6.9 Hz, 1H), 1.59 (d, J = 6.7 Hz, 3H).

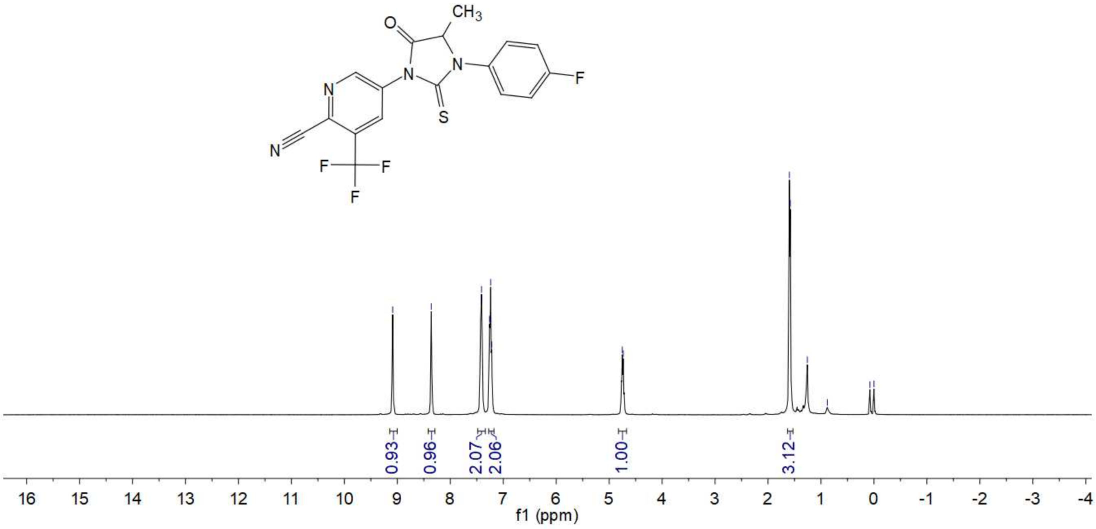

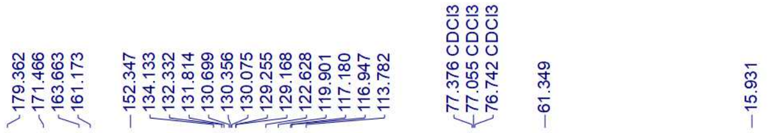

^{13}C NMR (101 MHz, $CDCl_3$) δ 179.36, 171.47, 163.66, 161.17, 152.35, 134.13, 132.33, 131.81, 130.70, 130.36, 130.08, 129.26, 129.17, 122.63, 119.90, 117.18, 116.95, 113.78, 61.35, 15.93.

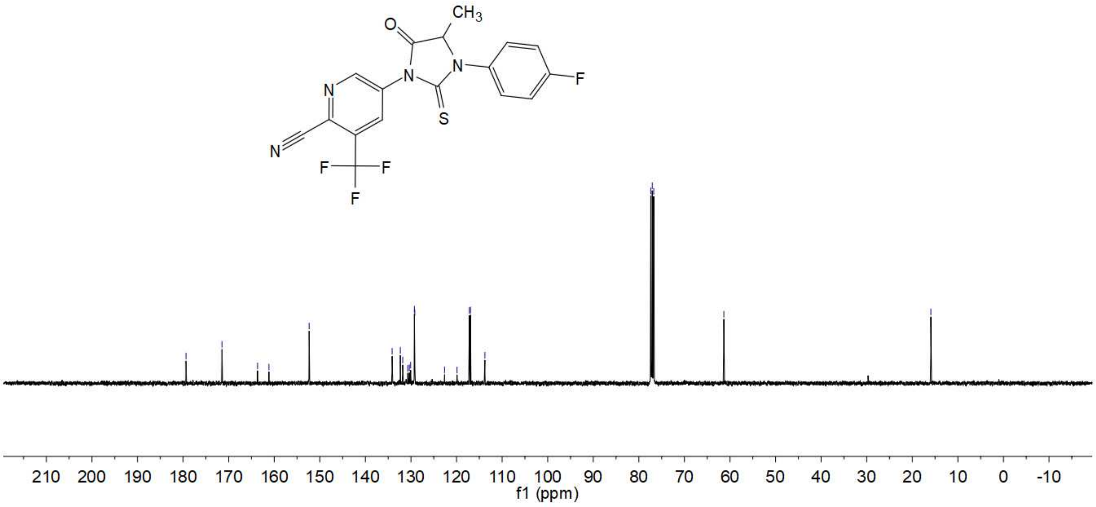

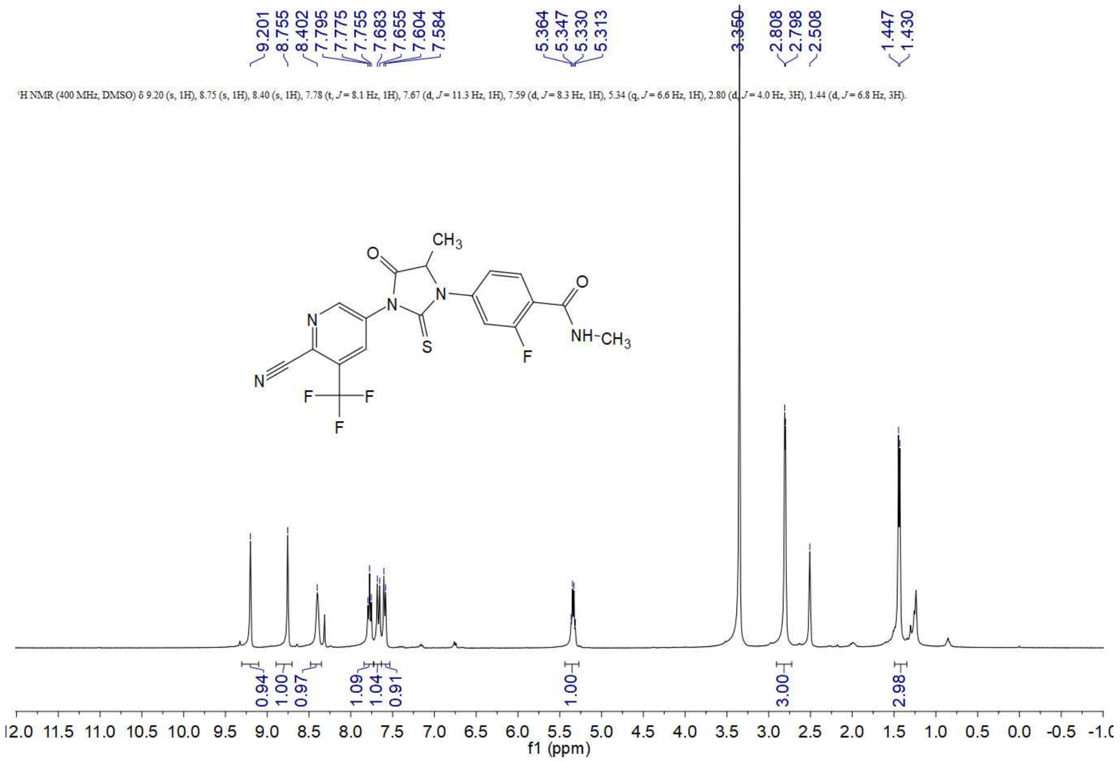

^{1}H NMR (400 MHz, DMSO) δ 9.20 (s, 1H), 8.75 (s, 1H), 8.40 (s, 1H), 7.78 (t, J = 8.1 Hz, 1H), 7.67 (d, J = 11.3 Hz, 1H), 7.59 (d, J = 8.3 Hz, 1H), 5.34 (q, J = 6.6 Hz, 1H), 2.80 (d, J = 4.0 Hz, 3H), 1.44 (d, J = 6.8 Hz, 3H).

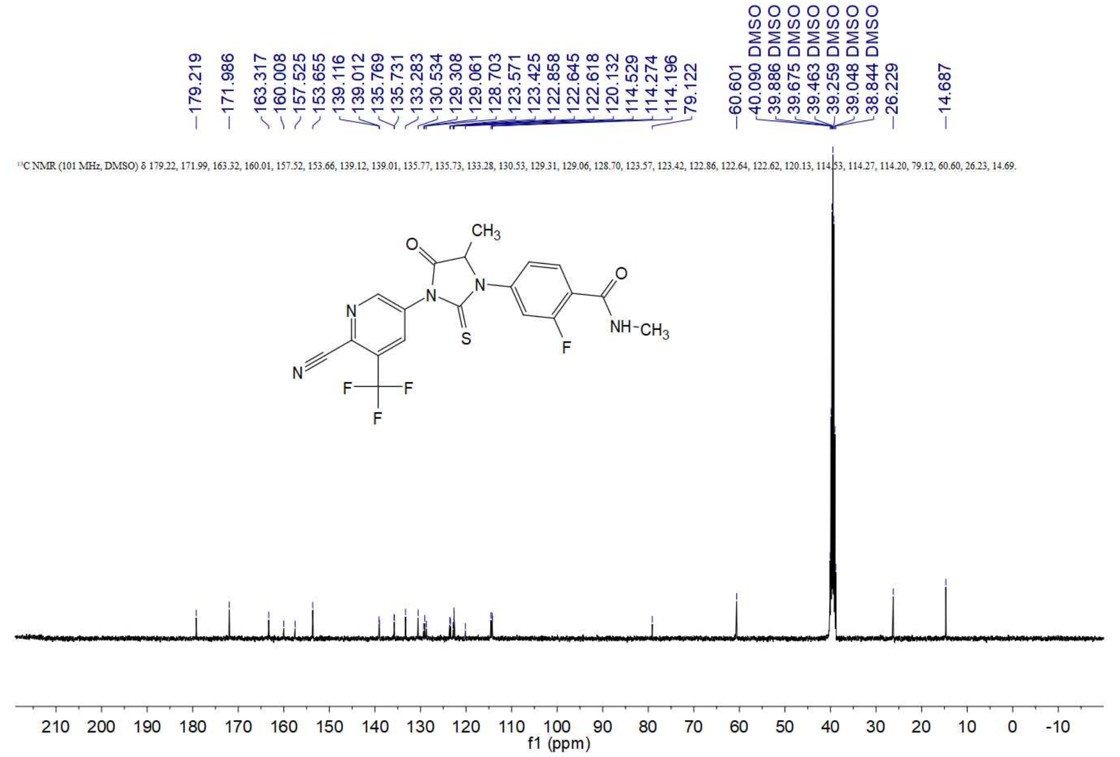

^{13}C NMR (101 MHz, DMSO) δ 179.22, 171.99, 163.32, 160.01, 157.52, 153.66, 139.12, 139.01, 135.77, 135.73, 133.28, 130.53, 129.31, 129.06, 128.70, 123.57, 123.42, 122.86, 122.64, 122.62, 120.13, 114.53, 114.27, 114.20, 79.12, 60.60, 26.23, 14.69.

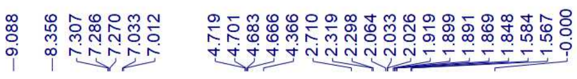

^{1}H NMR (400 MHz, $CDCl_3$) δ 9.09 (s, 1H), 8.36 (s, 1H), 7.30 (d, J = 8.4 Hz, 2H), 7.02 (d, J = 8.5 Hz, 2H), 4.69 (q, J = 7.0 Hz, 1H), 4.37 (s, 1H), 2.71 (s, 2H), 2.32 (s, 3H), 2.30 (s, 2H), 2.08 – 2.00 (m, 2H), 1.88 (dd, J = 14.6, 5.8 Hz, 2H), 1.58 (d, J = 7.0 Hz, 3H).

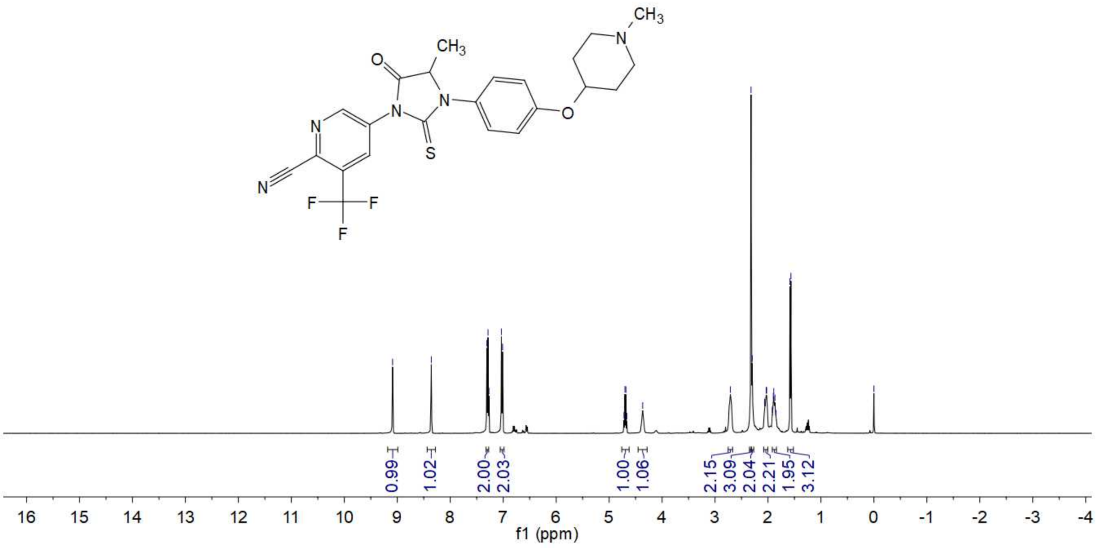

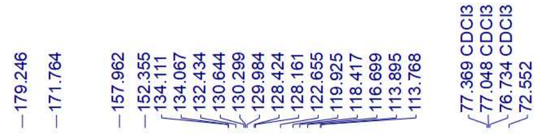

^{13}C NMR (101 MHz, $CDCl_3$) δ 179.25, 171.76, 157.96, 152.35, 134.11, 134.07, 132.43, 130.64, 130.30, 129.98, 128.42, 128.16, 122.66, 119.93, 118.42, 116.70, 113.89, 113.77, 72.55, 61.49, 52.61, 46.20, 30.80, 15.95.

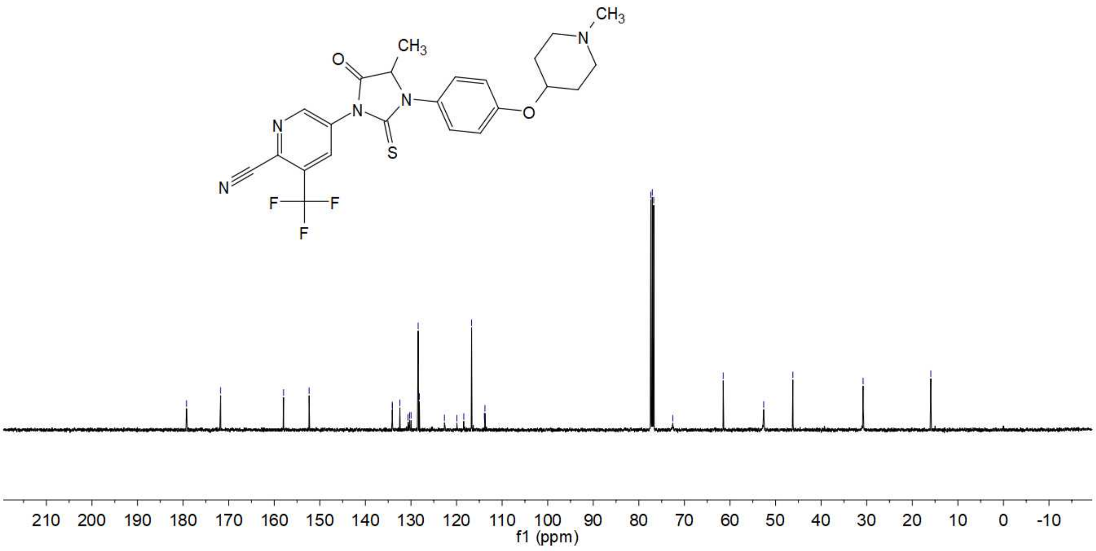

^{1}H ^{13}C, NMR and HRMS spectra of *compound* **6a-l**

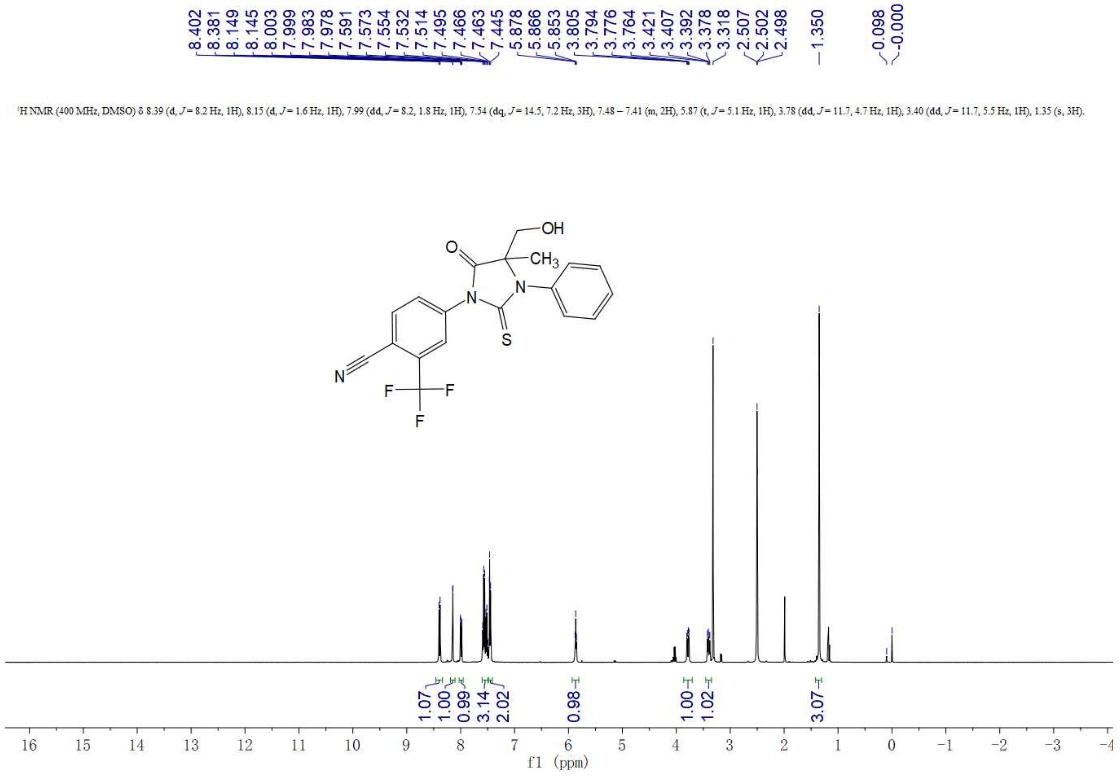

^{1}H NMR (400 MHz, DMSO) δ 8.39 (d, *J* = 8.2 Hz, 1H), 8.15 (d, *J* = 1.6 Hz, 1H), 7.99 (dd, *J* = 8.2, 1.8 Hz, 1H), 7.54 (dq, *J* = 14.5, 7.2 Hz, 3H), 7.48 – 7.41 (m, 2H), 5.87 (t, *J* = 5.1 Hz, 1H), 3.78 (dd, *J* = 11.7, 4.7 Hz, 1H), 3.40 (dd, *J* = 11.7, 5.5 Hz, 1H), 1.35 (s, 3H).

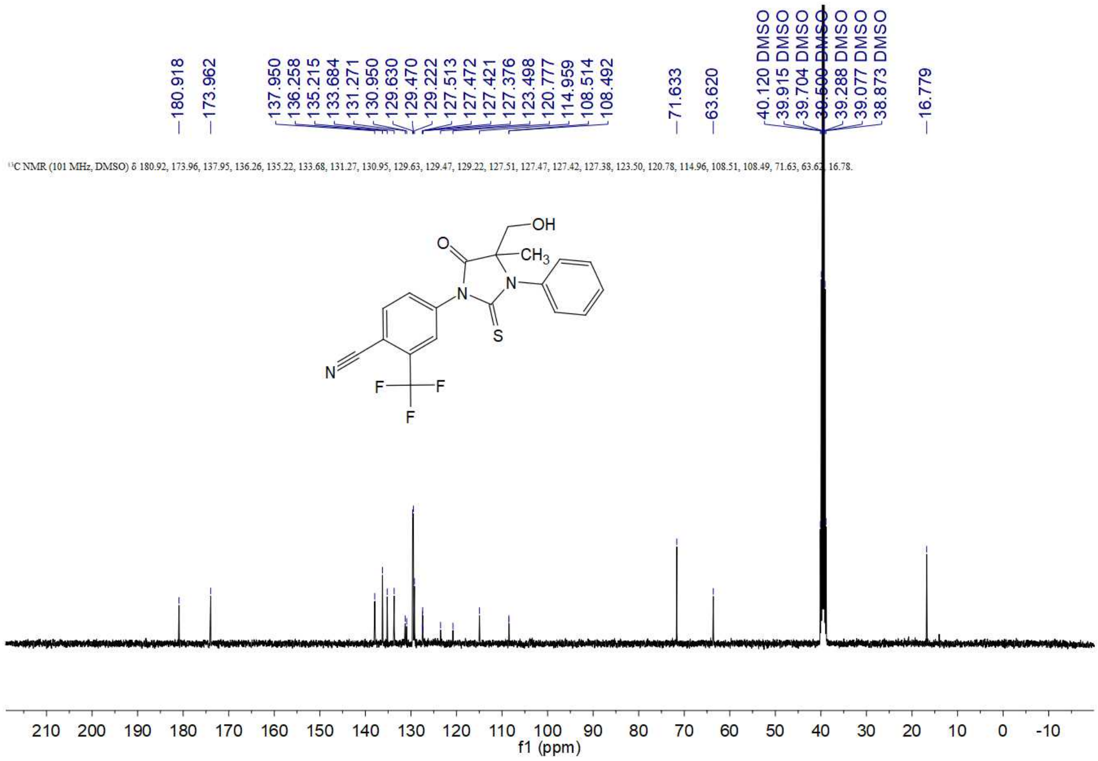

^{13}C NMR (101 MHz, DMSO) δ 180.92, 173.96, 137.95, 136.26, 135.22, 133.68, 131.27, 130.95, 129.63, 129.47, 129.22, 127.51, 127.47, 127.42, 127.38, 123.50, 120.78, 114.96, 108.51, 108.49, 71.63, 63.62, 16.78.

Elemental Composition Report

Single Mass Analysis

Tolerance = 500.0 PPM / DBE: min = -1.5, max = 50.0
Element prediction: Off

Monoisotopic Mass, Even Electron Ions
2 formula(e) evaluated with 1 results within limits (up to 50 closest results for each mass)
Elements Used:
C: 19-19 H: 14-15 N: 3-3 O: 2-2 F: 3-3 Na: 0-1 S: 1-1
JJY-A00193-062 28 (0.571)
1: TOF MS ES+

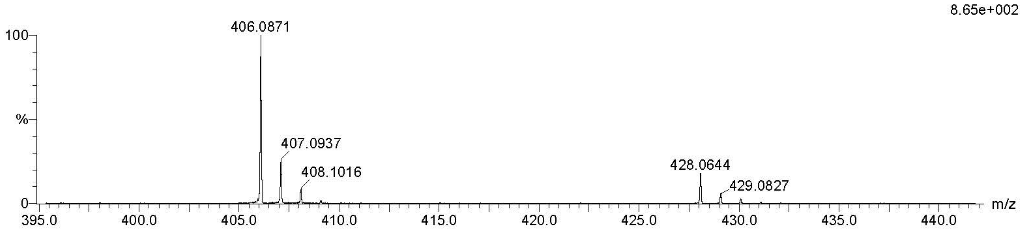

Minimum:				-1.5	
Maximum:		5.0	500.0	50.0	
Mass	Calc. Mass	mDa	PPM	DBE	Formula
406.0871	406.0837	3.4	8.4	12.5	C19 H15 N3 O2 F3 S

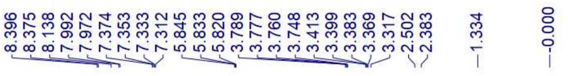

^{1}H NMR (400 MHz, DMSO) δ 8.39 (d, *J* = 8.2 Hz, 1H), 8.14 (s, 1H), 7.98 (d, *J* = 8.2 Hz, 1H), 7.36 (d, *J* = 8.2 Hz, 2H), 7.32 (d, *J* = 8.3 Hz, 2H), 5.83 (t, *J* = 5.1 Hz, 1H), 3.77 (dd, *J* = 11.6, 4.8 Hz, 1H), 3.39 (dd, *J* = 11.6, 5.6 Hz, 1H), 2.38 (s, 3H), 1.33 (s, 3H).

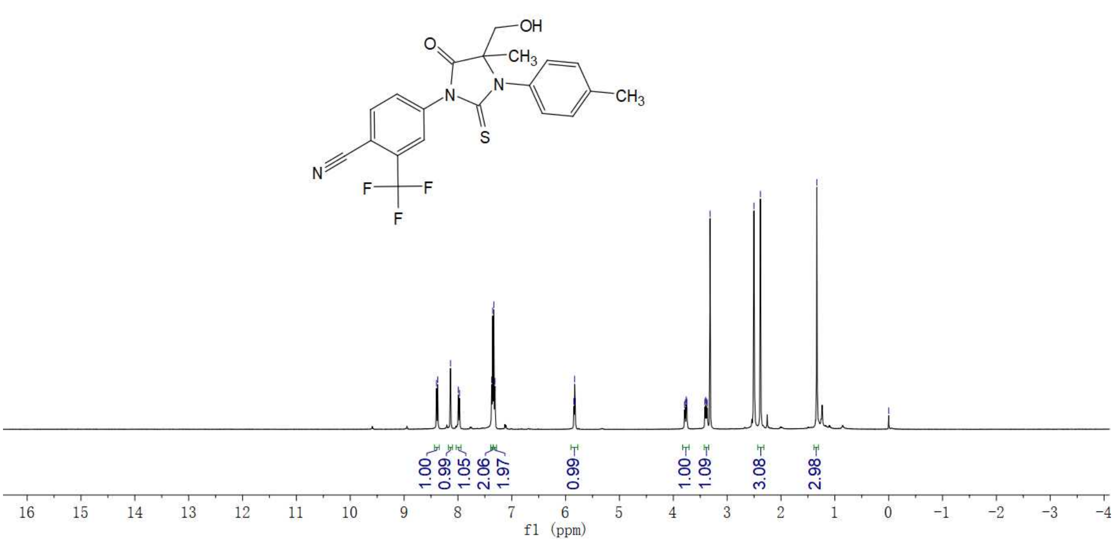

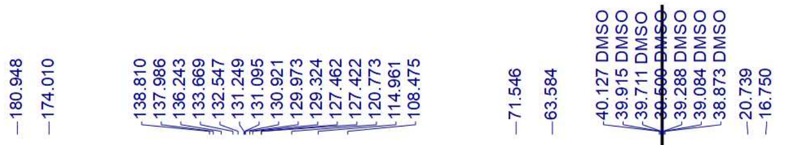

^{13}C NMR (101 MHz, DMSO) δ 180.95, 174.01, 138.81, 137.99, 136.24, 133.67, 132.55, 131.25, 131.09, 130.92, 129.97, 129.32, 127.46, 127.42, 120.77, 114.96, 108.47, 71.55, 63.58, 20.74, 16.75.

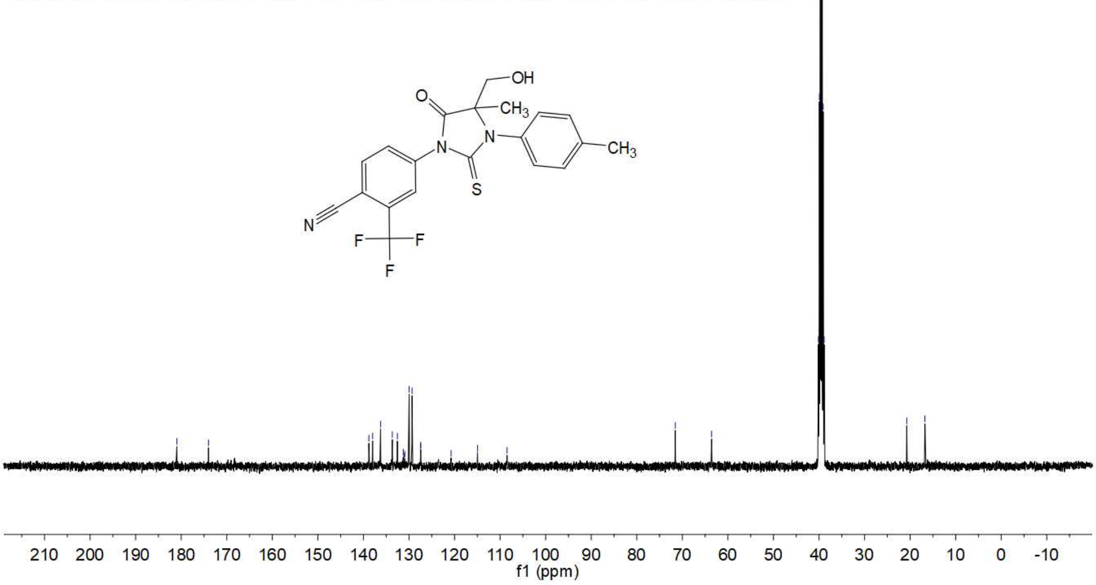

Single Mass Analysis
Tolerance = 500.0 PPM / DBE: min = -1.5, max = 50.0
Element prediction: Off

Monoisotopic Mass, Even Electron Ions
1 formula(e) evaluated with 1 results within limits (up to 50 closest results for each mass)
Elements Used:
C: 20-20 H: 17-17 N: 3-3 O: 2-2 F: 3-3 S: 1-1
JJY-A00193-014 27 (0.554)
1: TOF MS ES+

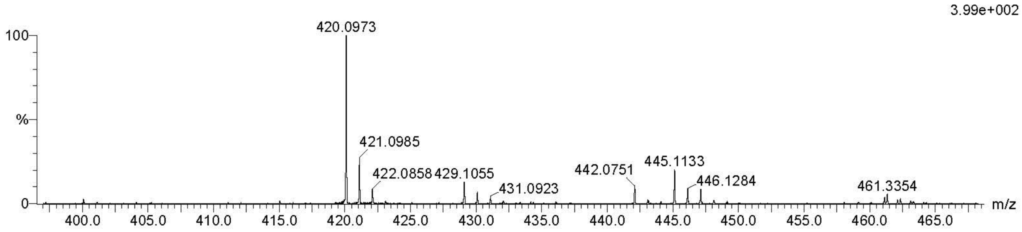

Minimum:				-1.5	
Maximum:		5.0	500.0	50.0	
Mass	Calc. Mass	mDa	PPM	DBE	Formula
420.0973	420.0994	-2.1	-5.0	12.5	C20 H17 N3 O2 F3 S

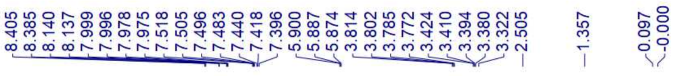

^{1}H NMR (400 MHz, DMSO) δ 8.39 (d, J = 8.2 Hz, 1H), 8.14 (d, J = 0.9 Hz, 1H), 7.99 (dd, J = 8.2, 1.3 Hz, 1H), 7.50 (dd, J = 8.8, 5.1 Hz, 2H), 7.42 (t, J = 8.8 Hz, 2H), 5.89 (t, J = 5.2 Hz, 1H), 3.79 (dd, J = 11.7, 4.9 Hz, 1H), 3.40 (dd, J = 11.7, 5.7 Hz, 1H), 1.36 (s, 3H).

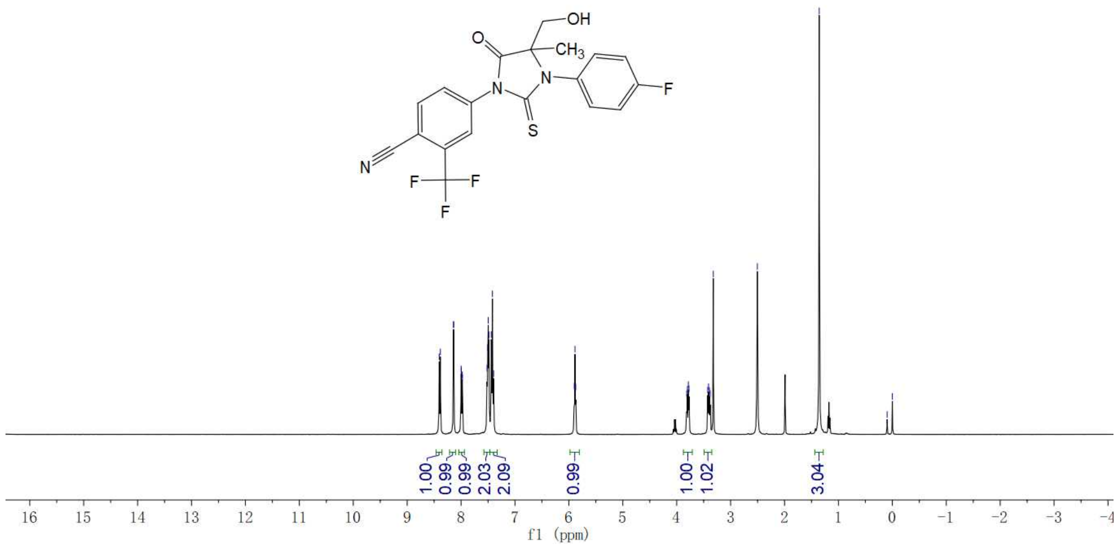

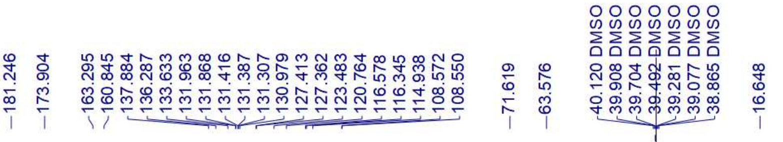

^{13}C NMR (101 MHz, DMSO) δ 181.25, 173.90, 163.29, 160.84, 137.88, 136.29, 133.63, 131.96, 131.87, 131.42, 131.39, 131.31, 130.98, 127.41, 127.36, 123.48, 120.76, 116.58, 116.34, 114.94, 108.57, 108.55, 71.62, 63.58, 16.65.

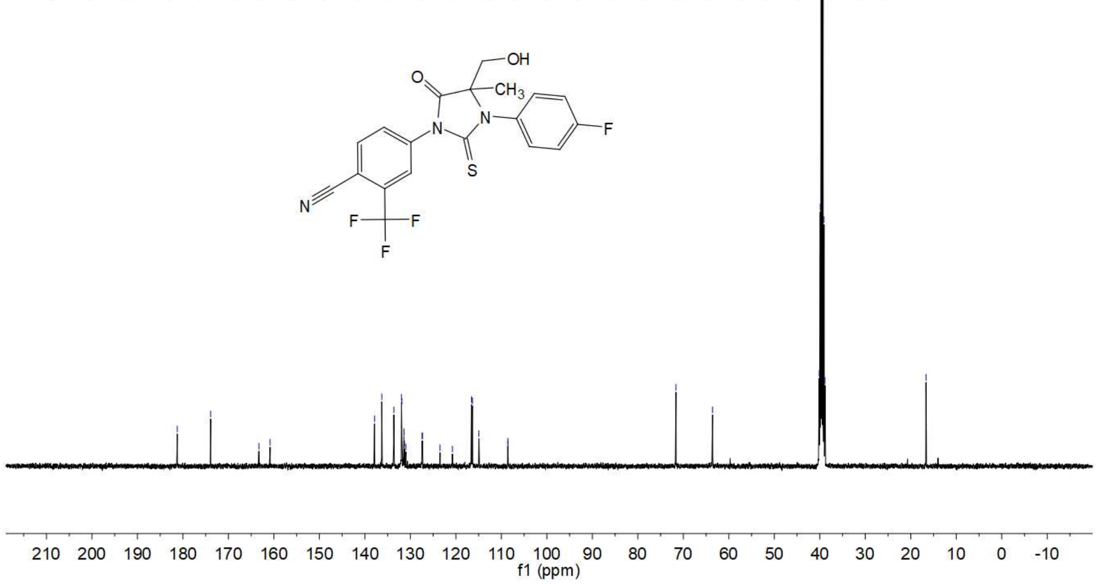

Single Mass Analysis
Tolerance = 500.0 PPM / DBE: min = -1.5, max = 50.0
Element prediction: Off

Monoisotopic Mass, Even Electron Ions
1 formula(e) evaluated with 1 results within limits (up to 50 closest results for each mass)
Elements Used:
C: 19-19 H: 14-14 N: 3-3 O: 2-2 F: 4-4 S: 1-1
JJY-A00193-039 25 (0.520) Cm (24:25)
1: TOF MS ES+

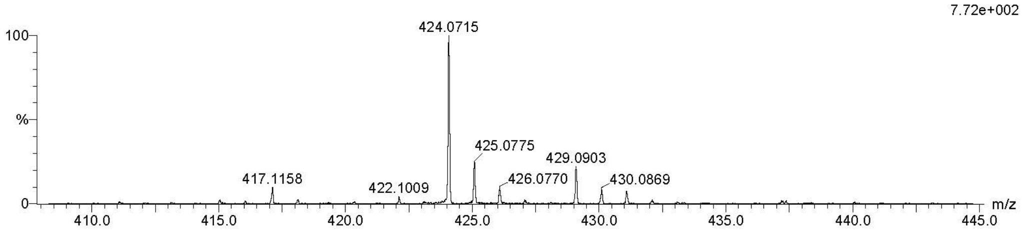

Minimum:				-1.5	
Maximum:		5.0	500.0	50.0	
Mass	Calc. Mass	mDa	PPM	DBE	Formula
424.0715	424.0743	-2.8	-6.6	12.5	C19 H14 N3 O2 F4 S

^{1}H NMR (400 MHz, DMSO) δ 8.40 (d, J = 8.2 Hz, 1H), 8.15 (s, 1H), 7.99 (d, J = 8.2 Hz, 1H), 7.66 (d, J = 8.5 Hz, 2H), 7.48 (d, J = 8.5 Hz, 2H), 5.90 (t, J = 4.9 Hz, 1H), 3.80 (dd, J = 11.7, 4.6 Hz, 1H), 3.42 (dd, J = 11.7, 5.6 Hz, 1H), 1.37 (s, 3H).

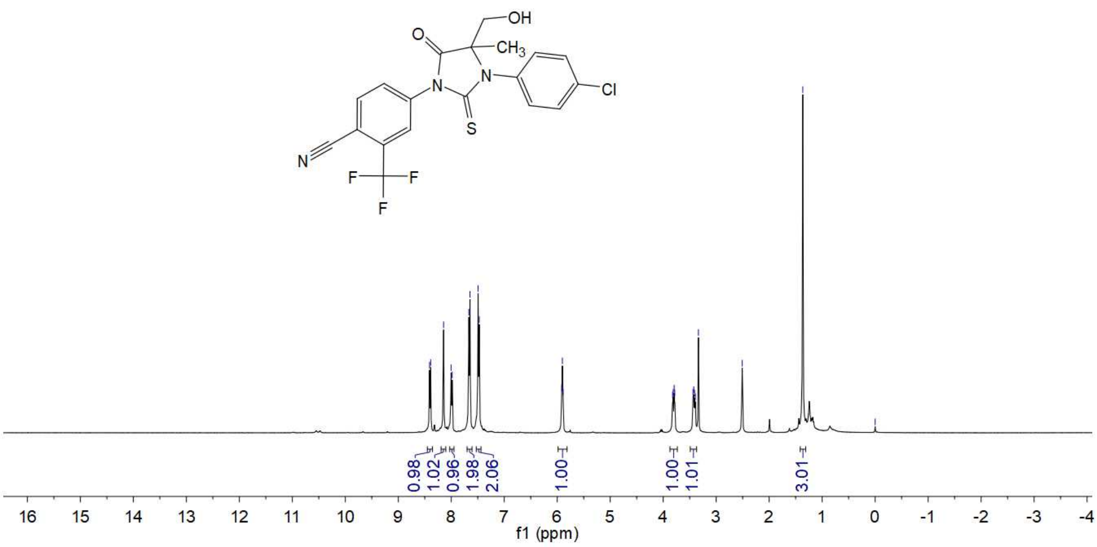

^{13}C NMR (101 MHz, DMSO) δ 181.10, 173.86, 137.83, 136.31, 134.13, 133.95, 133.63, 131.59, 131.31, 130.99, 129.65, 127.41, 127.36, 123.48, 120.76, 114.93, 108.60, 71.71, 63.60, 16.66.

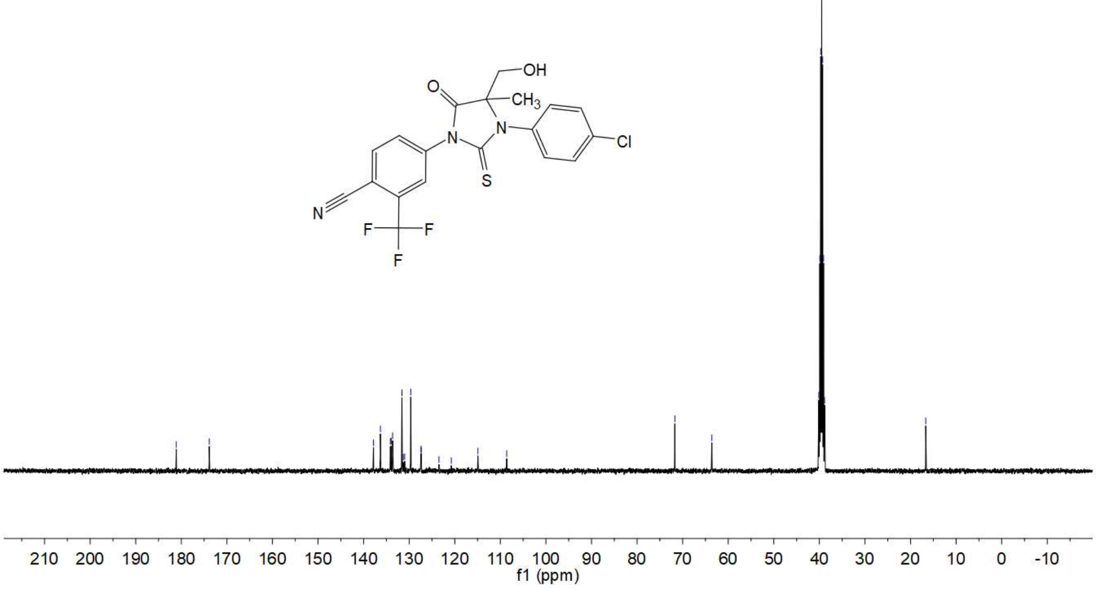

Elemental Composition Report

Single Mass Analysis

Tolerance = 500.0 PPM / DBE: min = -1.5, max = 50.0
Element prediction: Off

Monoisotopic Mass, Even Electron Ions
1 formula(e) evaluated with 1 results within limits (up to 50 closest results for each mass)
Elements Used:
C: 19-19 H: 14-14 N: 3-3 O: 2-2 F: 3-3 S: 1-1 Cl: 1-1
JJY-A00193-056 30 (0.605)
1: TOF MS ES+

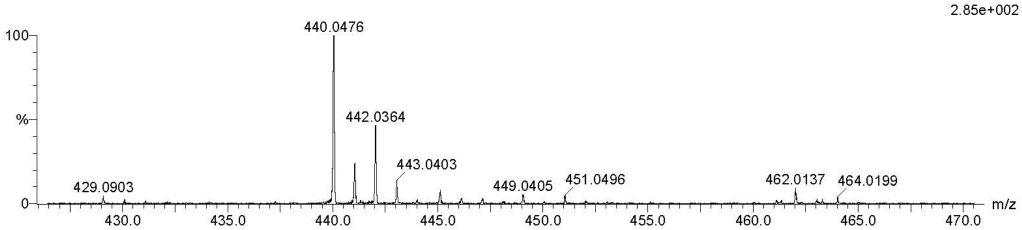

Minimum:				-1.5	
Maximum:		5.0	500.0	50.0	
Mass	Calc. Mass	mDa	PPM	DBE	Formula
440.0476	440.0447	2.9	6.6	12.5	C19 H14 N3 O2 F3 S Cl

^{1}H NMR (400 MHz, DMSO) δ 8.40 (d, J = 8.3 Hz, 1H), 8.14 (d, J = 1.5 Hz, 1H), 7.98 (dd, J = 8.2, 1.7 Hz, 1H), 7.79 (d, J = 8.6 Hz, 2H), 7.41 (d, J = 8.6 Hz, 2H), 5.89 (t, J = 5.2 Hz, 1H), 3.79 (dd, J = 11.8, 4.7 Hz, 1H), 3.41 (dd, J = 11.8, 5.6 Hz, 1H), 1.36 (s, 3H).

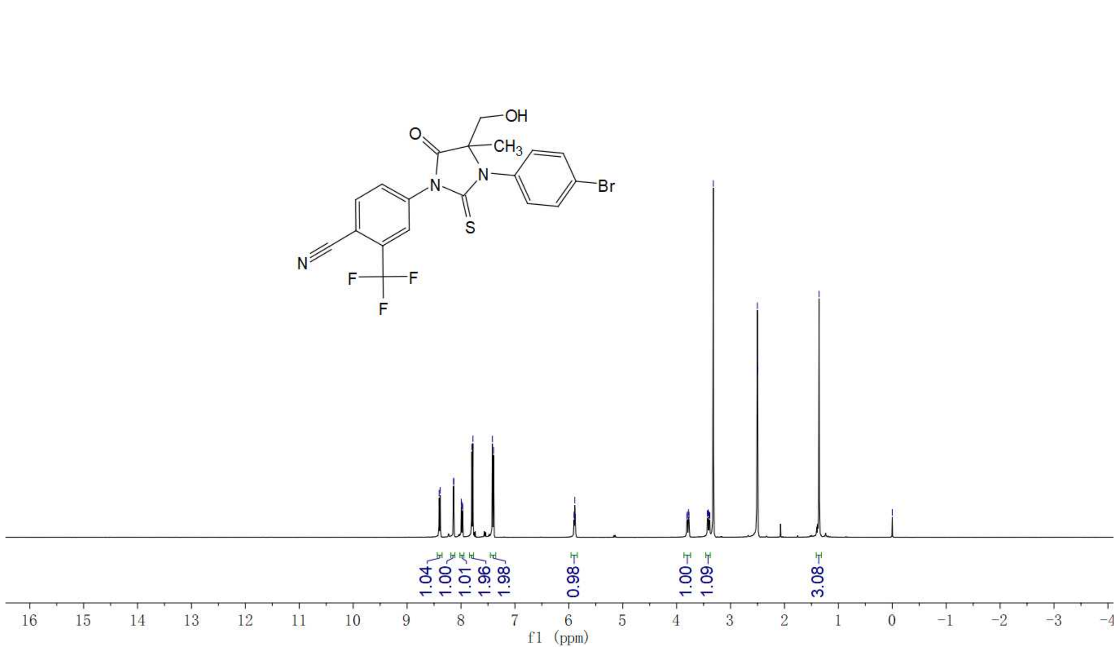

^{13}C NMR (101 MHz, DMSO) δ 181.00, 173.89, 137.79, 136.29, 134.51, 133.64, 132.62, 131.87, 131.33, 131.01, 127.43, 127.38, 123.45, 122.61, 120.72, 114.94, 108.59, 71.69, 63.54, 16.64.

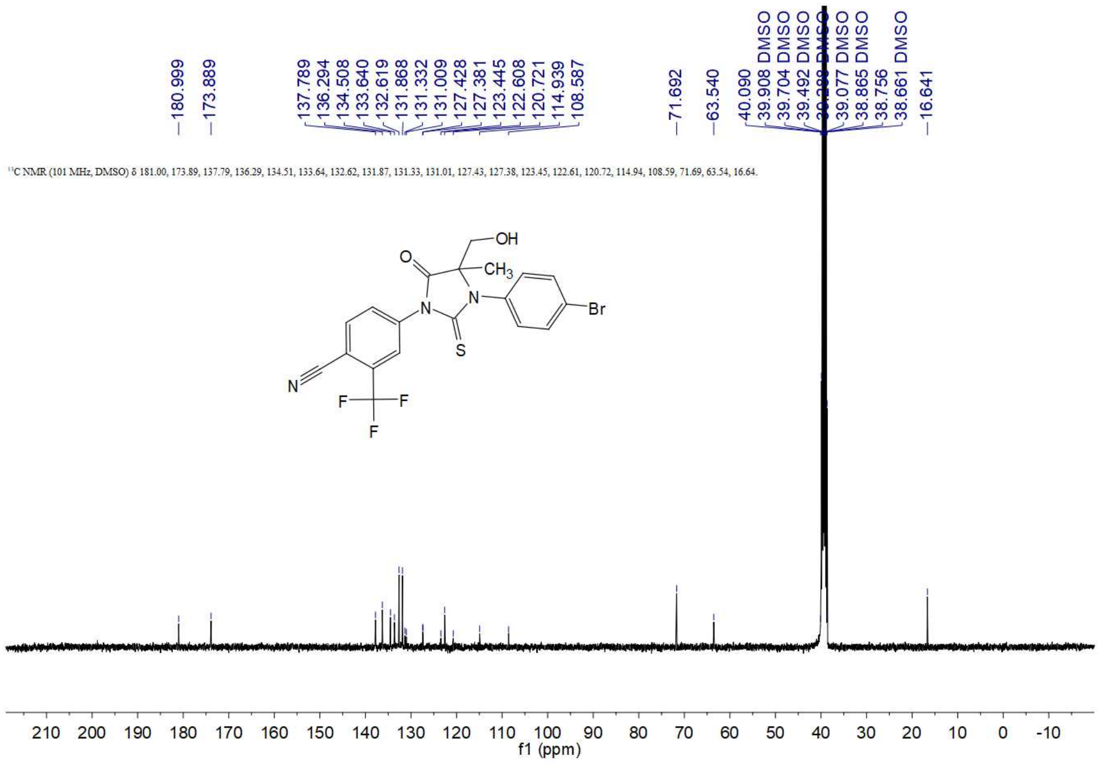

Elemental Composition Report

Single Mass Analysis

Tolerance = 500.0 PPM / DBE: min = -1.5, max = 50.0
Element prediction: Off

Monoisotopic Mass, Even Electron Ions
1 formula(e) evaluated with 1 results within limits (up to 50 closest results for each mass)
Elements Used:
C: 19-19 H: 14-14 N: 3-3 O: 2-2 F: 3-3 S: 1-1 Br: 1-1

JJY-A00193-060 23 (0.467)
1: TOF MS ES+

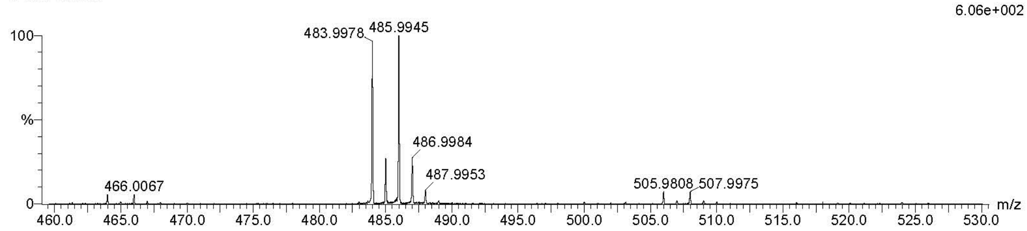

Minimum:				-1.5	
Maximum:		5.0	500.0	50.0	
Mass	Calc. Mass	mDa	PPM	DBE	Formula
483.9978	483.9942	3.6	7.4	12.5	C19 H14 N3 O2 F3 S Br

^{1}H NMR (400 MHz, DMSO) δ 8.39 (d, J = 8.3 Hz, 1H), 8.14 (d, J = 1.6 Hz, 1H), 7.99 (dd, J = 8.2, 1.7 Hz, 1H), 7.37 (d, J = 8.9 Hz, 2H), 7.10 (d, J = 8.9 Hz, 2H), 5.84 (t, J = 5.2 Hz, 1H), 3.83 (s, 3H), 3.78 (dd, J = 11.7, 4.8 Hz, 1H), 3.40 (dd, J = 11.7, 5.7 Hz, 1H), 1.34 (s, 3H).

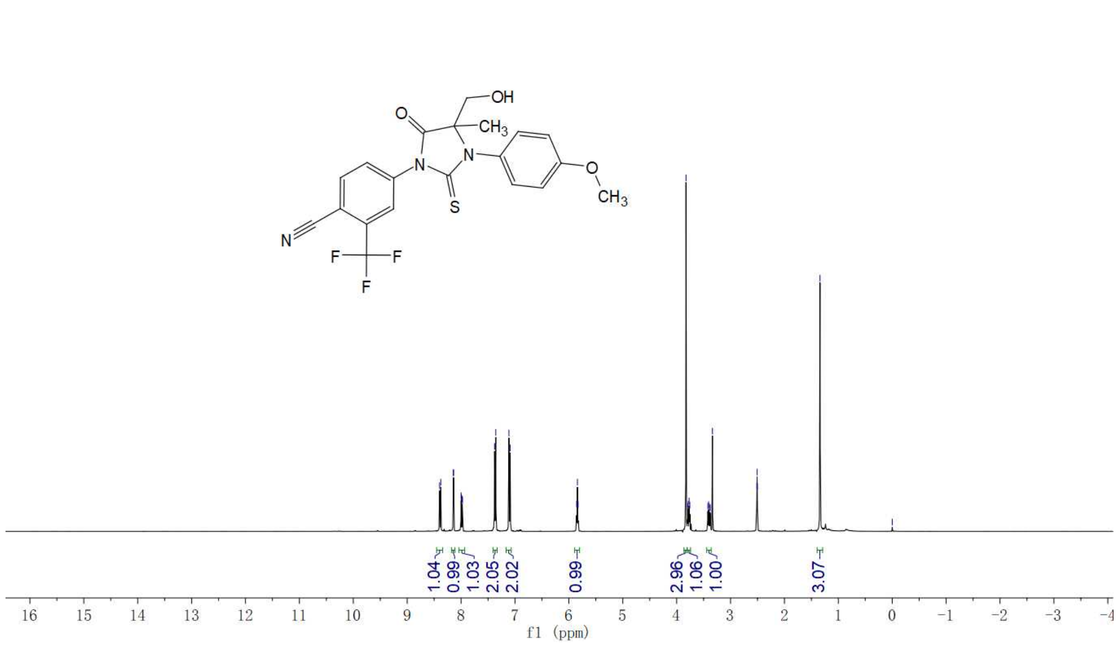

^{13}C NMR (101 MHz, DMSO) δ 181.17, 174.04, 159.50, 138.02, 136.23, 133.65, 130.73, 127.54, 127.45, 127.40, 123.50, 120.78, 114.96, 114.62, 108.45, 71.52, 63.56, 55.35, 16.71.

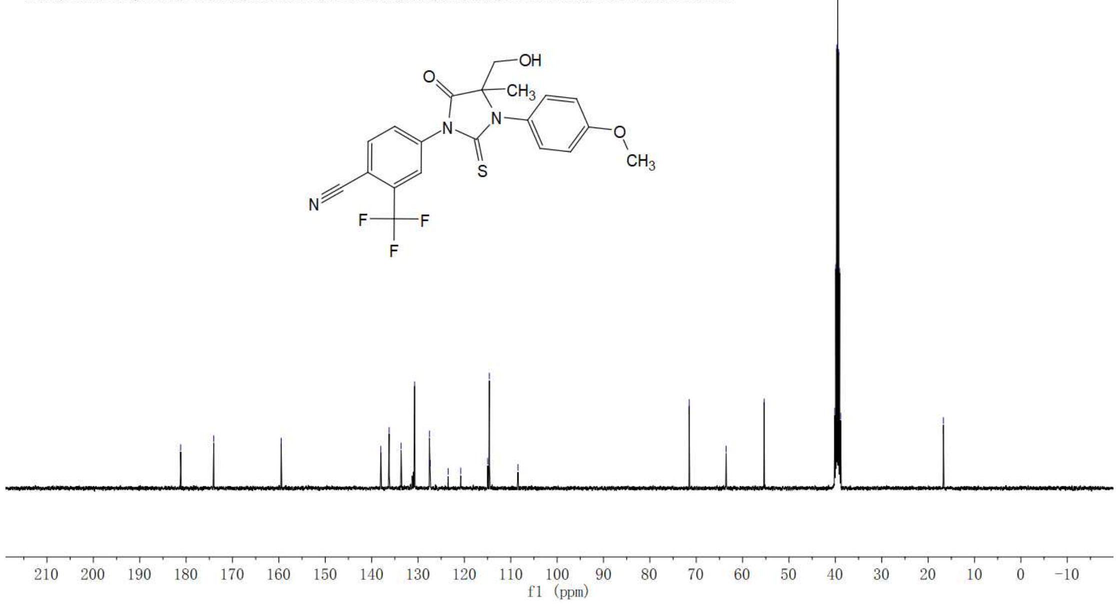

Single Mass Analysis
Tolerance = 500.0 PPM / DBE: min = -1.5, max = 50.0
Element prediction: Off

Monoisotopic Mass, Even Electron Ions
1 formula(e) evaluated with 1 results within limits (up to 50 closest results for each mass)
Elements Used:
C: 20-20 H: 17-17 N: 3-3 O: 3-3 F: 3-3 S: 1-1
JJY-A00193-043 43 (0.866)
1: TOF MS ES+

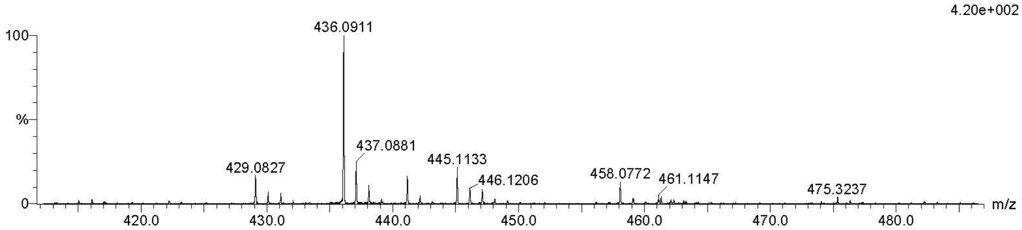

Minimum:				-1.5	
Maximum:		5.0	500.0	50.0	
Mass	Calc. Mass	mDa	PPM	DBE	Formula
436.0911	436.0943	-3.2	-7.3	12.5	C20 H17 N3 O3 F3 S

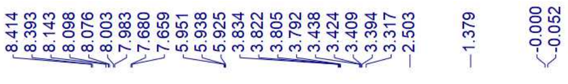

^{1}H NMR (400 MHz, DMSO) δ 8.40 (d, *J* = 8.2 Hz, 1H), 8.14 (s, 1H), 8.09 (d, *J* = 8.5 Hz, 2H), 7.99 (d, *J* = 8.2 Hz, 1H), 7.67 (d, *J* = 8.5 Hz, 2H), 5.94 (t, *J* = 5.3 Hz, 1H), 3.81 (dd, *J* = 11.9, 4.9 Hz, 1H), 3.42 (dd, *J* = 11.9, 5.8 Hz, 1H), 1.38 (s, 3H).

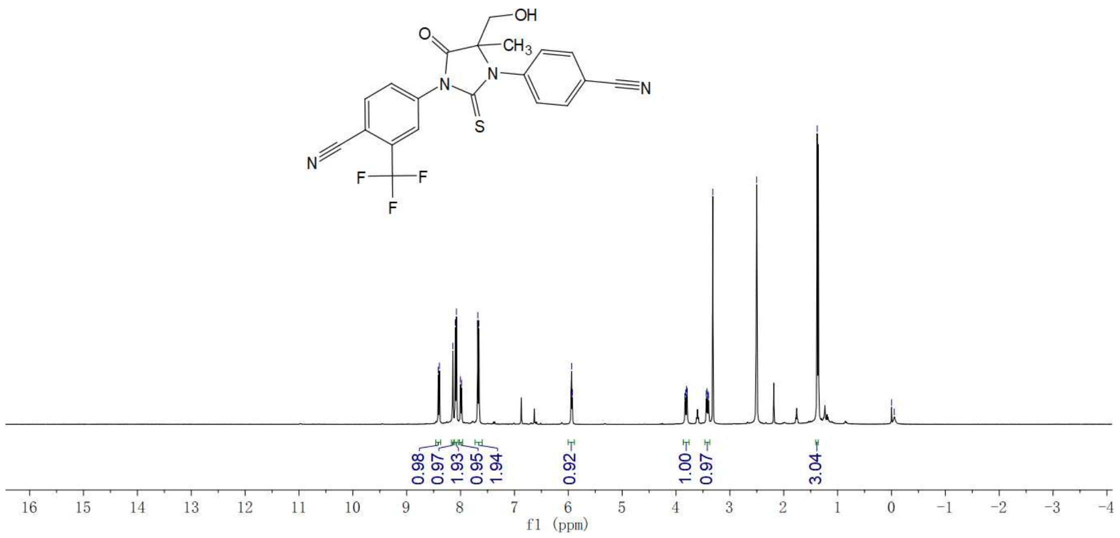

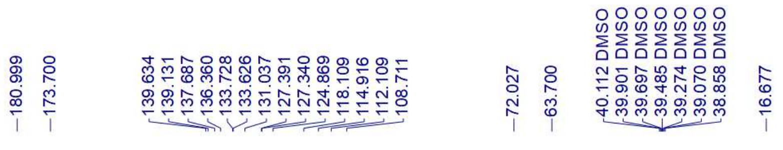

^{13}C NMR (101 MHz, DMSO) δ 181.00, 173.70, 139.63, 139.13, 137.69, 136.36, 133.73, 133.63, 131.04, 127.39, 127.34, 124.87, 118.11, 114.92, 112.11, 108.71, 72.03, 63.70, 16.68.

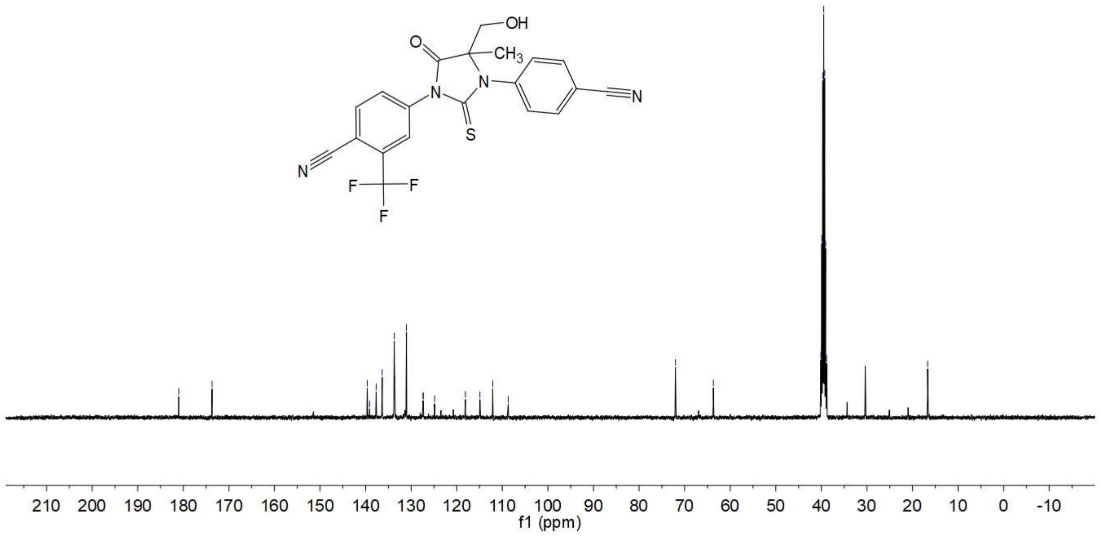

Elemental Composition Report **Page 1**

Single Mass Analysis
Tolerance = 500.0 PPM / DBE: min = -1.5, max = 50.0
Element prediction: Off

Monoisotopic Mass, Even Electron Ions
2 formula(e) evaluated with 1 results within limits (up to 50 closest results for each mass)
Elements Used:
C: 20-20 H: 13-14 N: 4-4 O: 2-2 F: 3-3 Na: 0-1 S: 1-1
JJY-A00193-076 22 (0.450)
1: TOF MS ES+

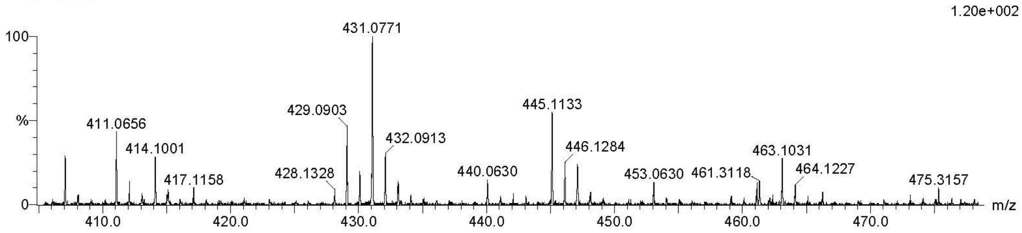

Mass	Calc. Mass	mDa	PPM	DBE	Formula
Minimum:				-1.5	
Maximum:		5.0	500.0	50.0	
431.0771	431.0790	-1.9	-4.4	14.5	C20 H14 N4 O2 F3 S

^{1}H NMR (400 MHz, DMSO) δ 8.45 (d, J = 8.9 Hz, 2H), 8.42 (d, J = 8.4 Hz, 1H), 8.17 (s, 1H), 8.02 (d, J = 8.3 Hz, 1H), 7.77 (d, J = 9.0 Hz, 2H), 5.98 (t, J = 5.3 Hz, 1H), 3.85 (dd, J = 11.9, 5.0 Hz, 1H), 3.45 (dd, J = 11.9, 5.8 Hz, 1H), 1.41 (s, 3H).

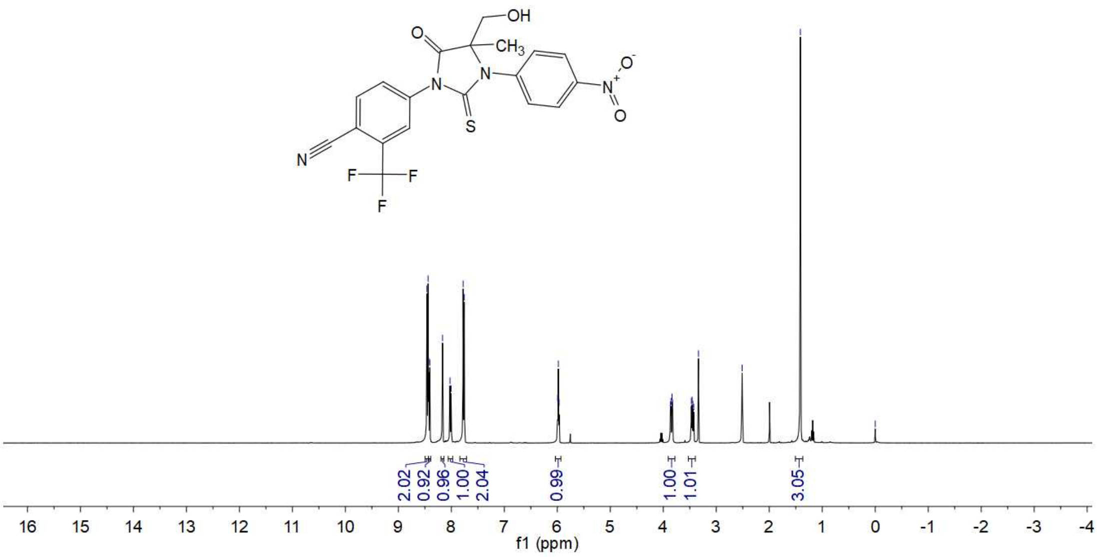

^{13}C NMR (101 MHz, DMSO) δ 181.06, 173.69, 147.57, 141.24, 137.69, 136.38, 133.65, 131.37, 131.06, 127.46, 127.41, 127.37, 127.32, 126.19, 124.85, 123.47, 120.74, 114.91, 108.74, 108.73, 72.13, 63.71, 16.70.

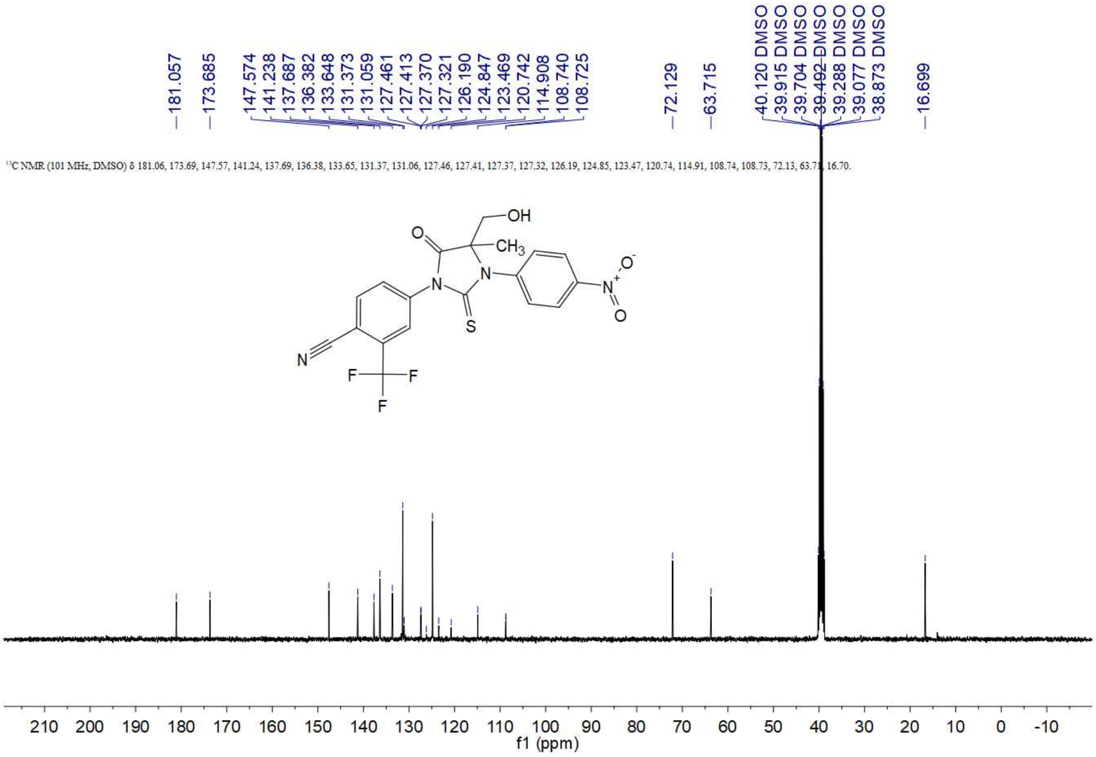

Elemental Composition Report

Single Mass Analysis
Tolerance = 500.0 PPM / DBE: min = -1.5, max = 50.0
Element prediction: Off

Monoisotopic Mass, Even Electron Ions
1 formula(e) evaluated with 1 results within limits (up to 50 closest results for each mass)
Elements Used:
C: 19-19 H: 14-14 N: 4-4 O: 4-4 F: 3-3 S: 1-1
JJY-A00193-082 20 (0.416) Cm (20:22)
1: TOF MS ES+

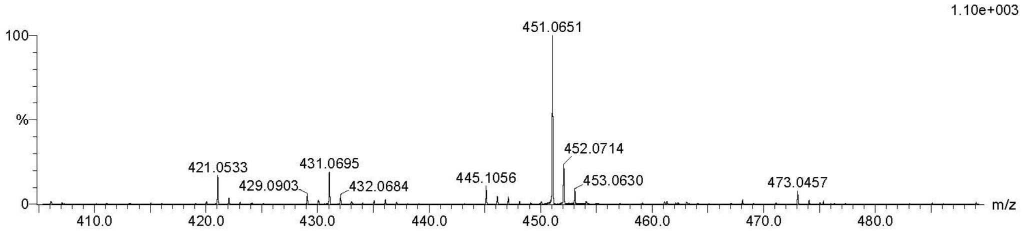

Minimum:				-1.5	
Maximum:		5.0	500.0	50.0	
Mass	Calc. Mass	mDa	PPM	DBE	Formula
451.0651	451.0688	-3.7	-8.2	13.5	C19 H14 N4 O4 F3 S

^{1}H NMR (400 MHz, DMSO) δ 8.41 (d, J = 8.2 Hz, 1H), 8.16 (s, 1H), 7.99 (t, J = 7.8 Hz, 3H), 7.70 (d, J = 8.1 Hz, 2H), 5.94 (t, J = 5.2 Hz, 1H), 3.82 (dd, J = 11.8, 4.9 Hz, 1H), 3.43 (dd, J = 11.8, 5.7 Hz, 1H), 1.39 (s, 3H).

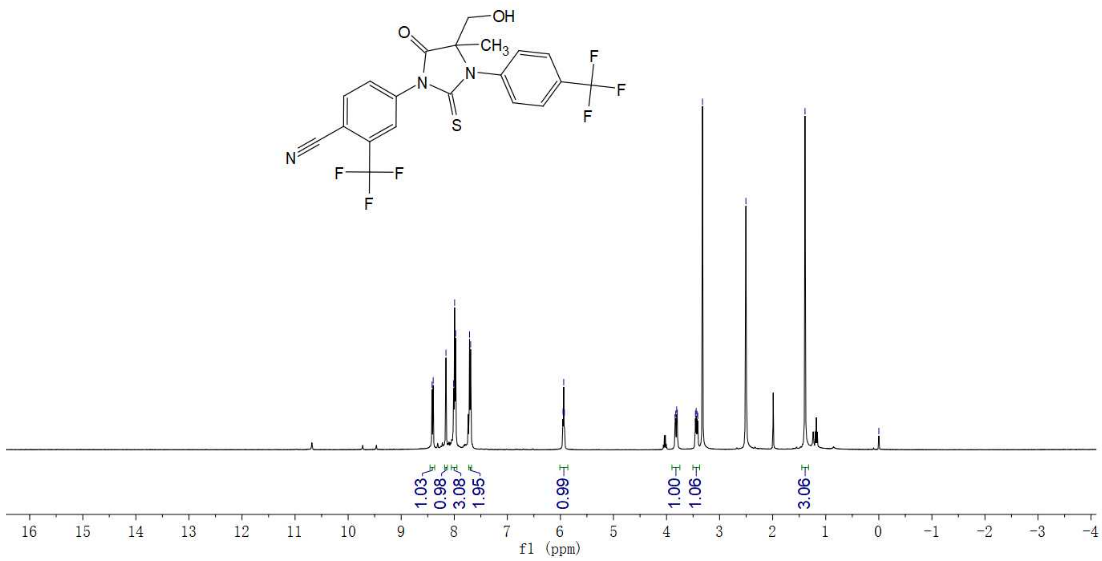

^{13}C NMR (101 MHz, DMSO) δ 181.11, 173.78, 139.09, 137.77, 136.32, 133.65, 131.37, 131.04, 130.86, 130.03, 129.72, 129.40, 129.08, 127.46, 127.42, 127.37, 127.32, 126.71, 126.68, 125.20, 123.47, 123.35, 122.50, 120.75, 119.79, 118.51, 114.91, 108.69, 108.67, 71.93, 63.67, 16.68.

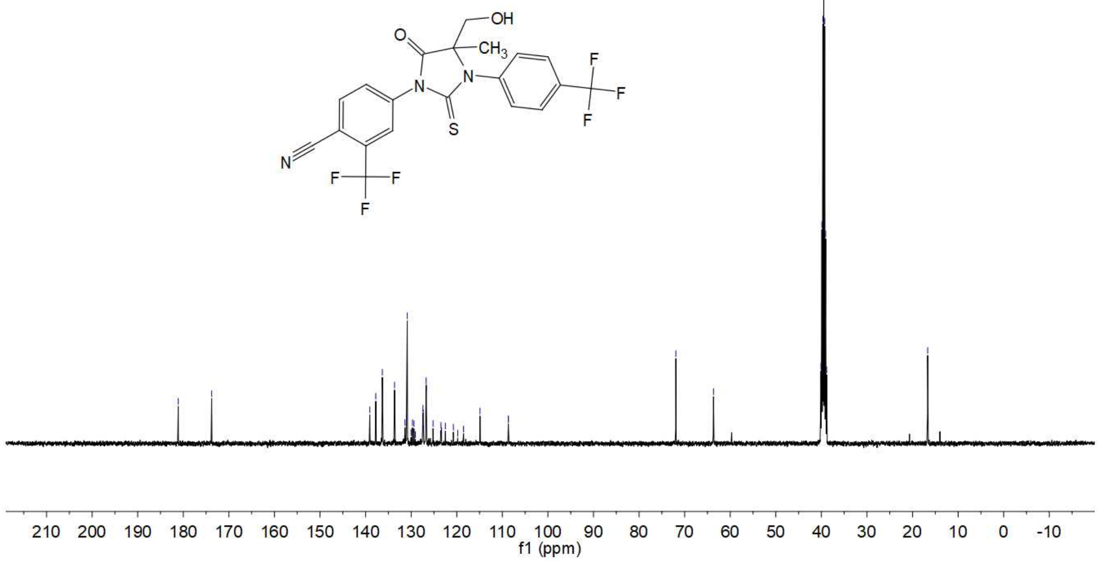

Elemental Composition Report

Single Mass Analysis

Tolerance = 500.0 PPM / DBE: min = -1.5, max = 50.0
Element prediction: Off

Monoisotopic Mass, Even Electron Ions
1 formula(e) evaluated with 1 results within limits (up to 50 closest results for each mass)
Elements Used:
C: 20-20 H: 13-14 N: 3-3 O: 2-2 F: 6-6 Na: 0-1 S: 1-1
JJY-A00193-086 30 (0.605) Cm (25:33)
1: TOF MS ES+

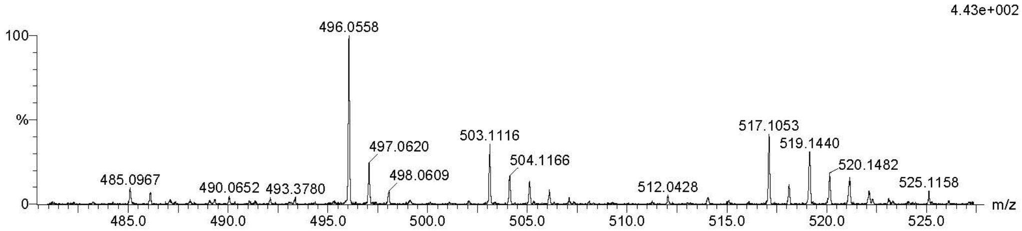

Minimum:				-1.5	
Maximum:		5.0	500.0	50.0	
Mass	Calc. Mass	mDa	PPM	DBE	Formula
496.0558	496.0530	2.8	5.6	12.5	C20 H13 N3 O2 F6 Na S

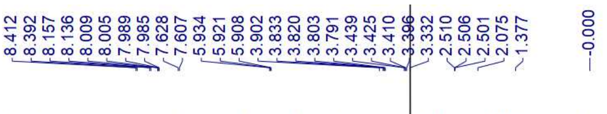

^{1}H NMR (400 MHz, DMSO) δ 8.40 (d, J = 8.3 Hz, 1H), 8.15 (d, J = 8.5 Hz, 3H), 8.00 (dd, J = 8.2, 1.6 Hz, 1H), 7.62 (d, J = 8.5 Hz, 2H), 5.92 (t, J = 5.3 Hz, 1H), 3.90 (s, 3H), 3.81 (dd, J = 11.8, 4.9 Hz, 1H), 3.42 (dd, J = 11.8, 5.7 Hz, 1H), 1.38 (s, 3H).

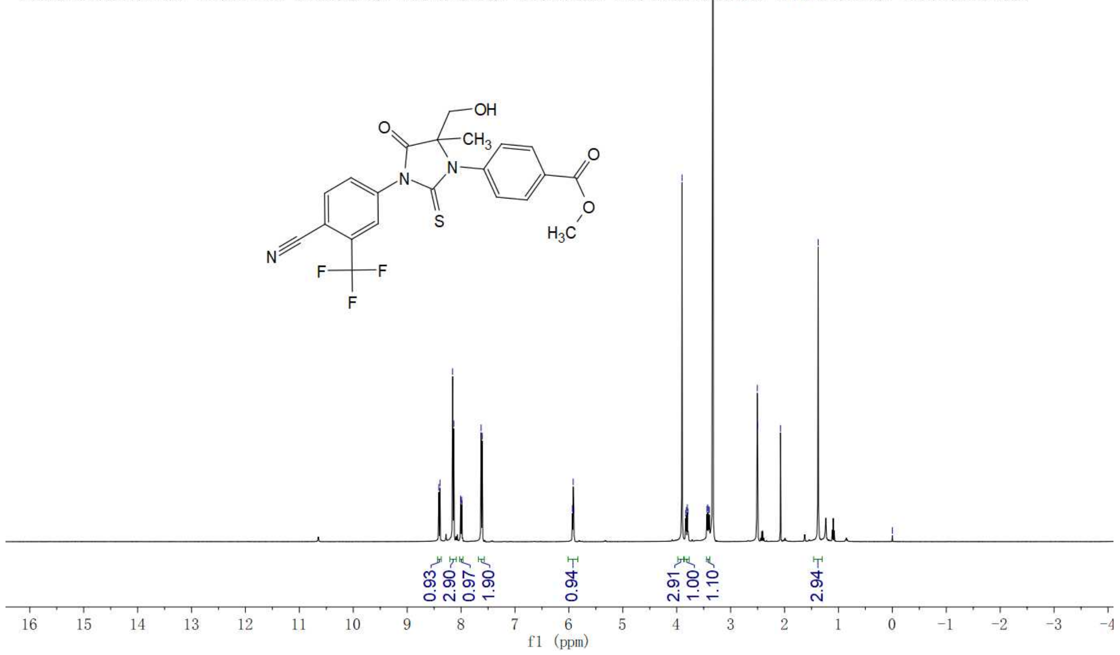

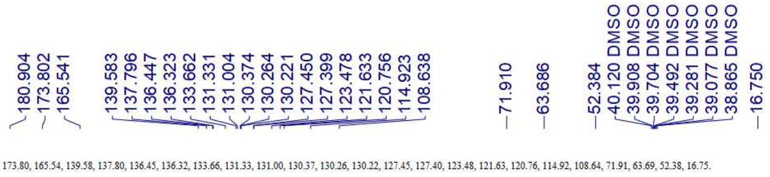

^{13}C NMR (101 MHz, DMSO) δ 180.90, 173.80, 165.54, 139.58, 137.80, 136.45, 136.32, 133.66, 131.33, 131.00, 130.37, 130.26, 130.22, 127.45, 127.40, 123.48, 121.63, 120.76, 114.92, 108.64, 71.91, 63.69, 52.38, 16.75.

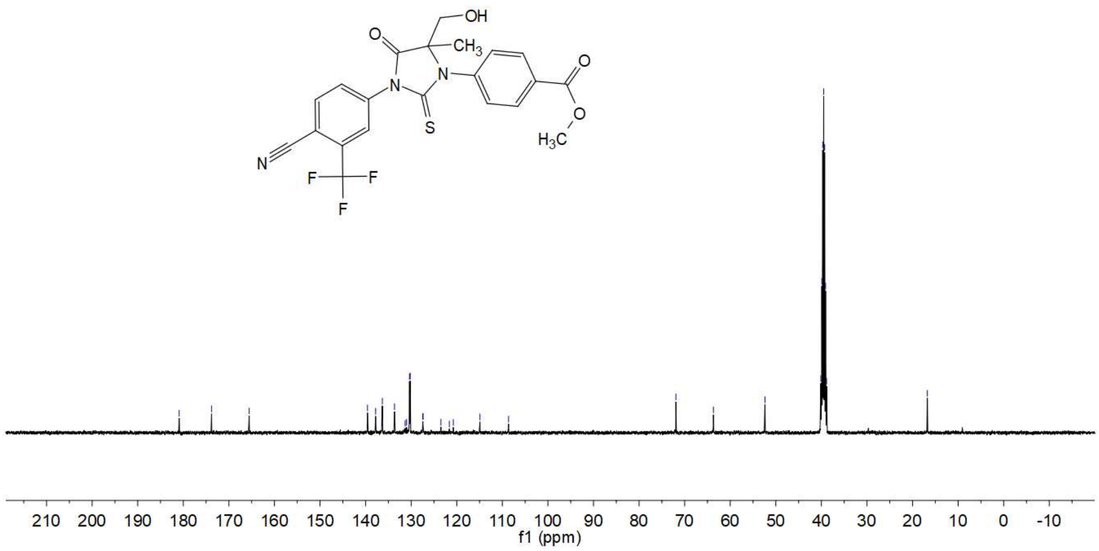

Elemental Composition Report

Single Mass Analysis

Tolerance = 500.0 PPM / DBE: min = -1.5, max = 50.0
Element prediction: Off

Monoisotopic Mass, Even Electron Ions
1 formula(e) evaluated with 1 results within limits (up to 50 closest results for each mass)
Elements Used:
C: 21-21 H: 17-17 N: 3-3 O: 4-4 F: 3-3 S: 1-1
JJY-A00193-080 24 (0.484)
1: TOF MS ES+

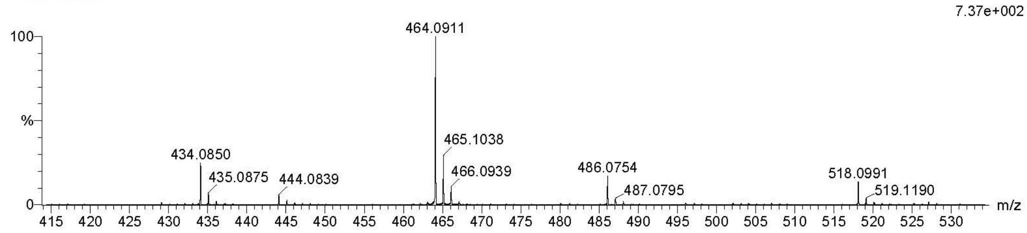

Minimum:				-1.5	
Maximum:		5.0	500.0	50.0	
Mass	Calc. Mass	mDa	PPM	DBE	Formula
464.0911	464.0892	1.9	4.1	13.5	C21 H17 N3 O4 F3 S

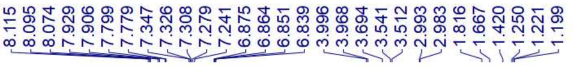

^{1}H NMR (400 MHz, $CDCl_3$) δ 8.09 (t, J = 8.2 Hz, 1H), 7.92 (d, J = 9.4 Hz, 2H), 7.79 (d, J = 8.2 Hz, 1H), 7.34 (d, J = 8.3 Hz, 1H), 7.29 (d, J = 11.8 Hz, 1H), 6.86 (dd, J = 9.8, 4.6 Hz, 1H), 3.98 (d, J = 11.3 Hz, 1H), 3.69 (s, 1H), 3.53 (d, J = 11.4 Hz, 1H), 2.99 (d, J = 4.1 Hz, 3H), 1.42 (s, 3H).

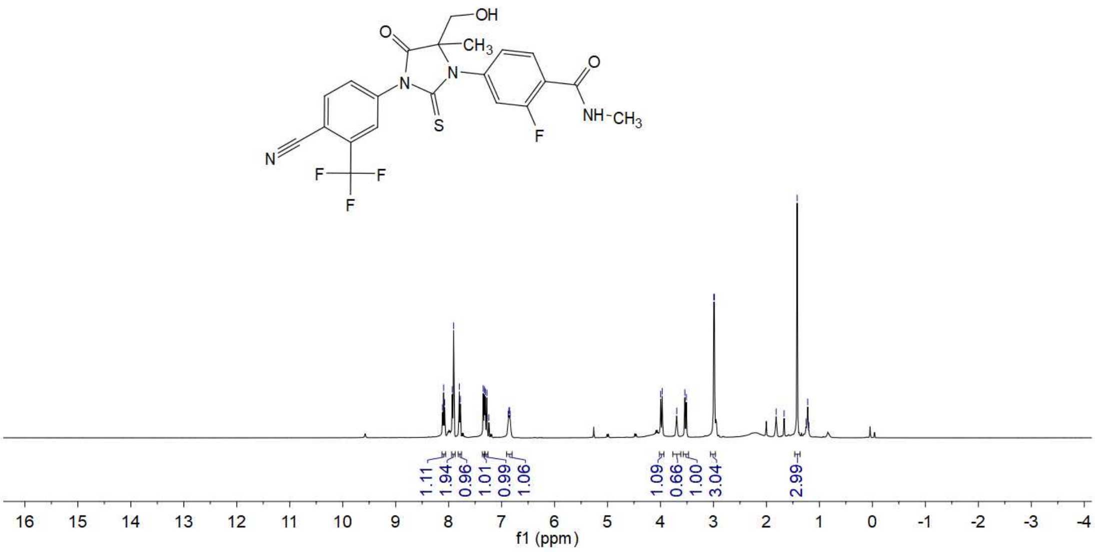

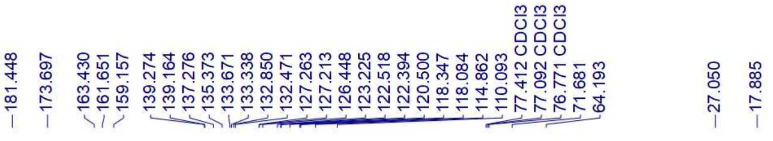

^{13}C NMR (101 MHz, $CDCl_3$) δ 181.45, 173.70, 163.43, 161.65, 159.16, 139.27, 139.16, 137.28, 135.37, 133.67, 133.34, 132.85, 132.47, 127.26, 127.21, 126.45, 123.22, 122.52, 122.39, 120.50, 118.35, 118.08, 114.86, 110.09, 71.68, 64.19, 27.05, 17.88.

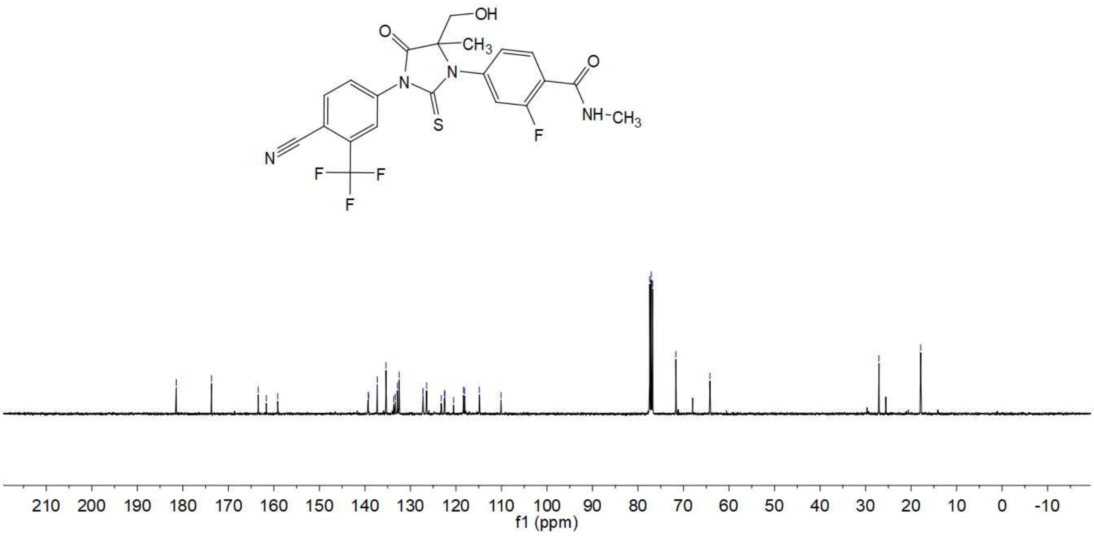

Single Mass Analysis

Tolerance = 500.0 PPM / DBE: min = -1.5, max = 50.0
Element prediction: Off

Monoisotopic Mass, Even Electron Ions
1 formula(e) evaluated with 1 results within limits (up to 50 closest results for each mass)
Elements Used:
C: 21-21 H: 17-17 N: 4-4 O: 3-3 F: 4-4 S: 1-1

JJY-A00222-065 29 (0.588)
1: TOF MS ES+

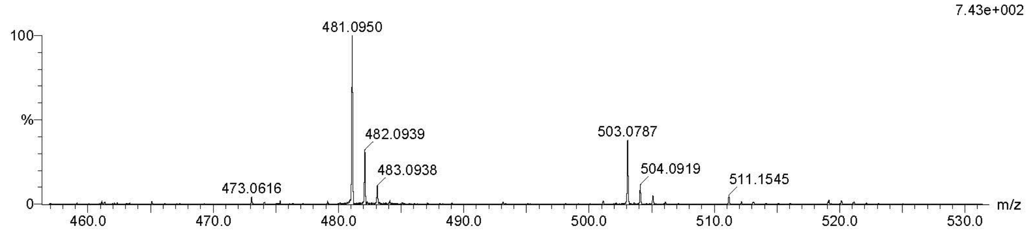

Minimum:				-1.5	
Maximum:		5.0	500.0	50.0	
Mass	Calc. Mass	mDa	PPM	DBE	Formula
481.0950	481.0958	-0.8	-1.7	13.5	C21 H17 N4 O3 F4 S

^{1}H NMR (400 MHz, $CDCl_3$) δ 7.95 (d, J = 7.9 Hz, 2H), 7.85 (d, J = 8.2 Hz, 1H), 7.35 (d, J = 8.8 Hz, 2H), 7.00 (d, J = 8.9 Hz, 2H), 4.42 (s, 1H), 3.96 (d, J = 11.2 Hz, 1H), 3.78 (s, 1H), 3.59 (d, J = 11.2 Hz, 1H), 2.86 – 2.76 (m, 2H), 2.55 (s, 2H), 2.41 (s, 3H), 2.11 (s, 2H), 1.93 (s, 2H), 1.43 (s, 3H).

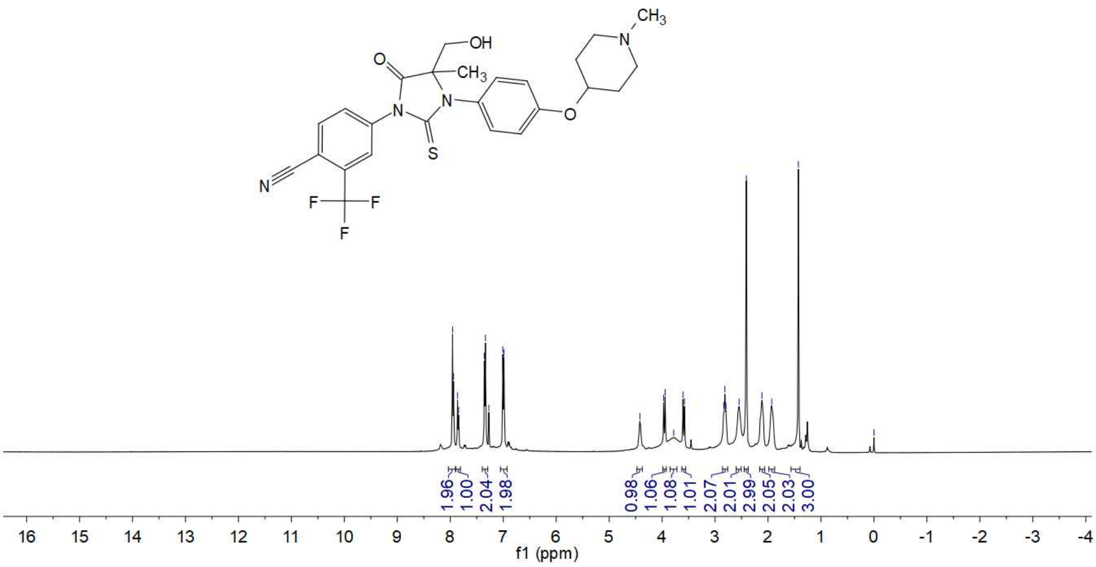

^{13}C NMR (101 MHz, $CDCl_3$) δ 181.74, 174.32, 157.98, 137.58, 135.26, 133.56, 133.23, 132.53, 130.84, 127.46, 127.30, 127.25, 123.29, 120.56, 116.54, 114.91, 109.92, 77.28, 71.45, 63.99, 51.88, 45.50, 29.73, 17.85.

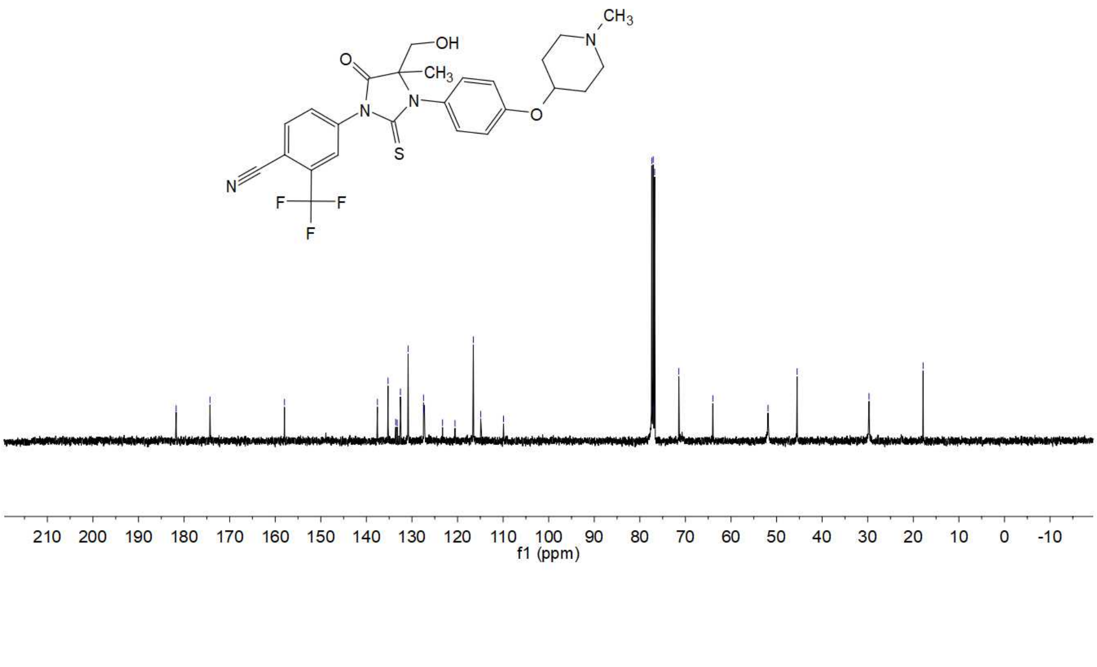

Single Mass Analysis
Tolerance = 500.0 PPM / DBE: min = -1.5, max = 50.0
Element prediction: Off

Monoisotopic Mass, Even Electron Ions
1 formula(e) evaluated with 1 results within limits (up to 50 closest results for each mass)
Elements Used:
C: 25-25 H: 26-26 N: 4-4 O: 3-3 F: 3-3 S: 1-1
JJY-A00222-066 44 (0.883)
1: TOF MS ES+

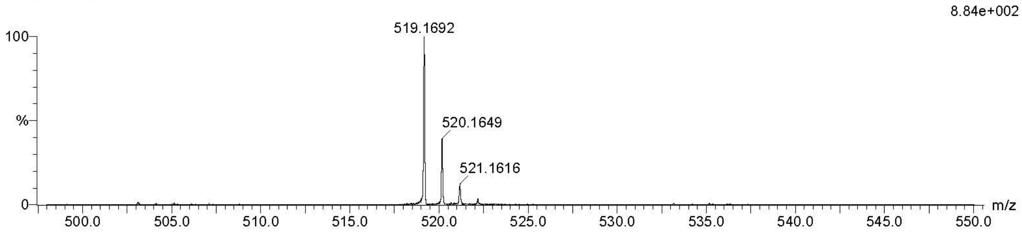

Minimum:				-1.5	
Maximum:		5.0	500.0	50.0	
Mass	Calc. Mass	mDa	PPM	DBE	Formula
519.1692	519.1678	1.4	2.7	13.5	C25 H26 N4 O3 F3 S

^{1}H, ^{13}C, NMR and HRMS spectra of *compound* **7a-j**

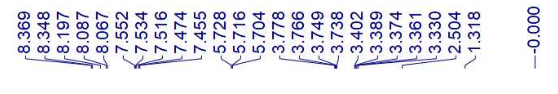

^{1}H NMR (400 MHz, DMSO) δ 8.36 (d, J = 8.3 Hz, 1H), 8.20 (s, 1H), 8.08 (d, J = 8.0 Hz, 1H), 7.56 – 7.43 (m, 5H), 5.72 (t, J = 4.7 Hz, 1H), 3.76 (dd, J = 11.5, 4.7 Hz, 1H), 3.38 (dd, J = 11.5, 5.2 Hz, 1H), 1.32 (s, 3H).

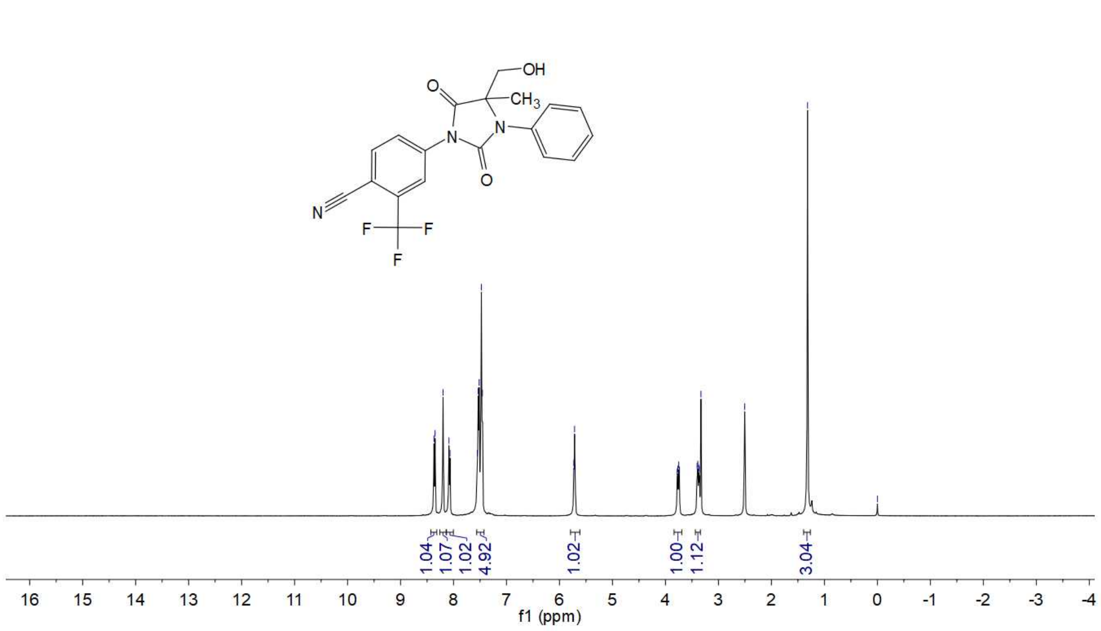

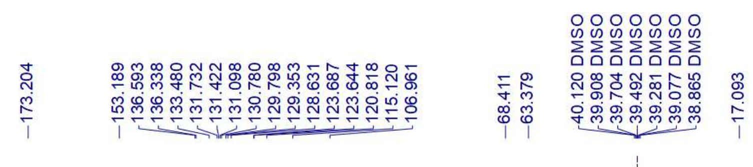

^{13}C NMR (101 MHz, DMSO) δ 173.20, 153.19, 136.59, 136.34, 133.48, 131.73, 131.42, 131.10, 130.78, 129.80, 129.35, 128.63, 123.69, 123.64, 120.82, 115.12, 106.96, 68.41, 63.38, 17.09.

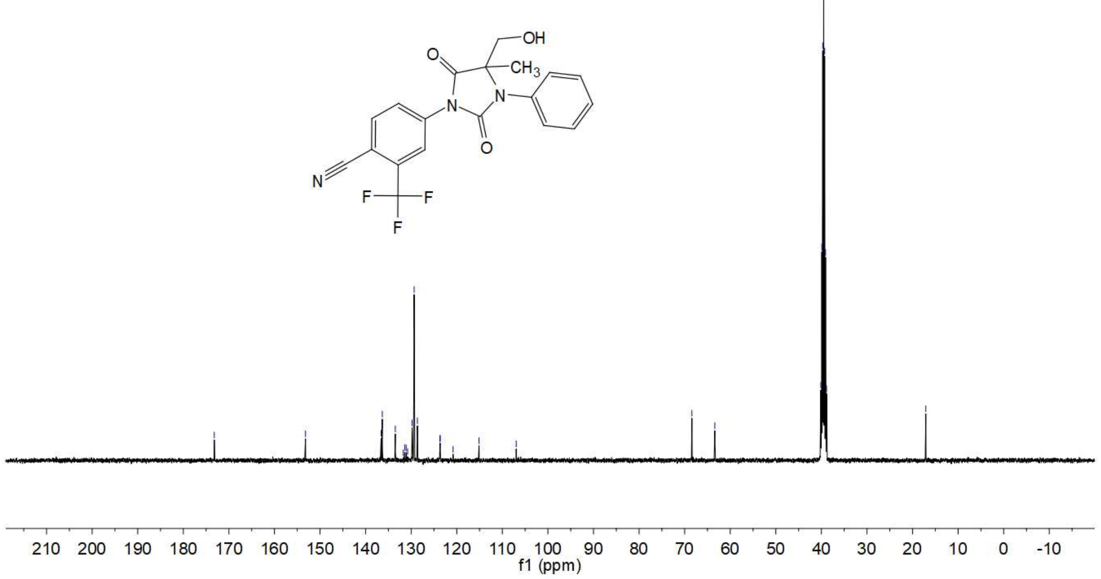

Elemental Composition Report

Single Mass Analysis

Tolerance = 500.0 PPM / DBE: min = -1.5, max = 50.0
Element prediction: Off

Monoisotopic Mass, Even Electron Ions
2 formula(e) evaluated with 1 results within limits (up to 50 closest results for each mass)
Elements Used:
C: 19-19 H: 14-15 N: 3-3 O: 3-3 F: 3-3 Na: 0-1
JJY-A00193-061 26 (0.537)
1: TOF MS ES+

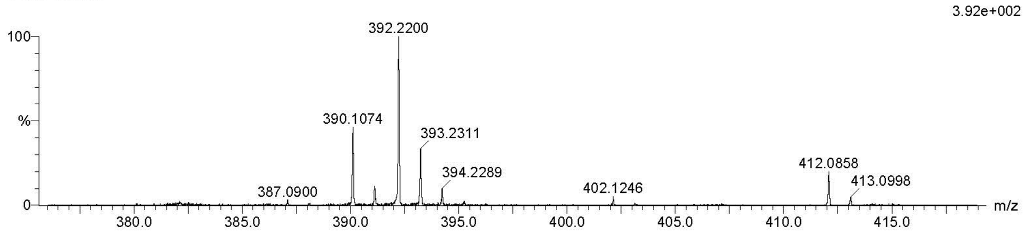

Minimum:				-1.5	
Maximum:		5.0	500.0	50.0	
Mass	Calc. Mass	mDa	PPM	DBE	Formula
390.1074	390.1066	0.8	2.1	12.5	C19 H15 N3 O3 F3

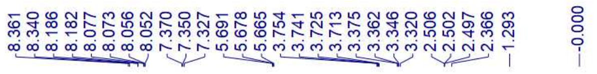

^{1}H NMR (400 MHz, DMSO) δ 8.35 (d, *J* = 8.4 Hz, 1H), 8.18 (d, *J* = 1.6 Hz, 1H), 8.06 (dd, *J* = 8.4, 1.7 Hz, 1H), 7.34 (d, *J* = 9.0 Hz, 4H), 5.68 (t, *J* = 5.1 Hz, 1H), 3.73 (dd, *J* = 11.6, 4.8 Hz, 1H), 3.35 (dd, *J* = 11.6, 4.8 Hz, 1H), 2.37 (s, 3H), 1.29 (s, 3H).

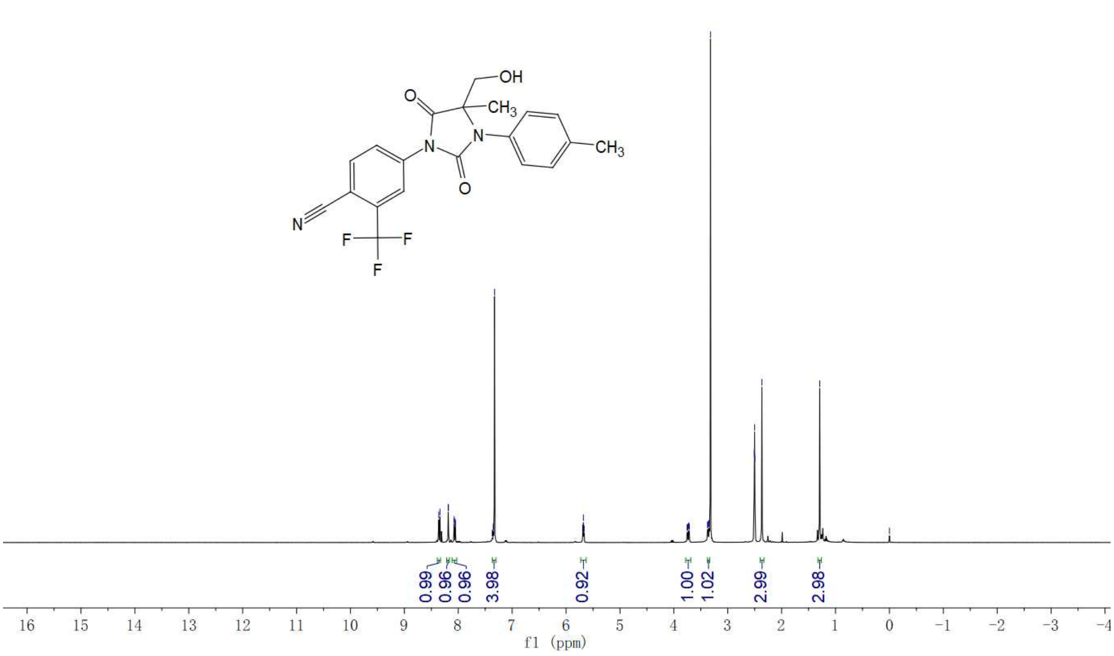

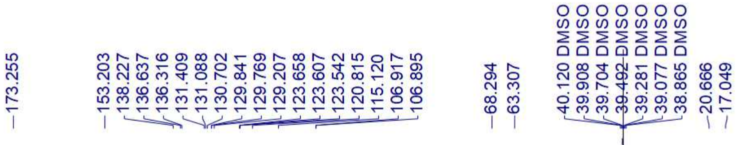

^{13}C NMR (101 MHz, DMSO) δ 173.25, 153.20, 138.23, 136.64, 136.32, 131.41, 131.09, 130.70, 129.84, 129.77, 129.21, 123.66, 123.61, 123.54, 120.82, 115.12, 106.92, 106.90, 68.29, 63.31, 20.67, 17.05.

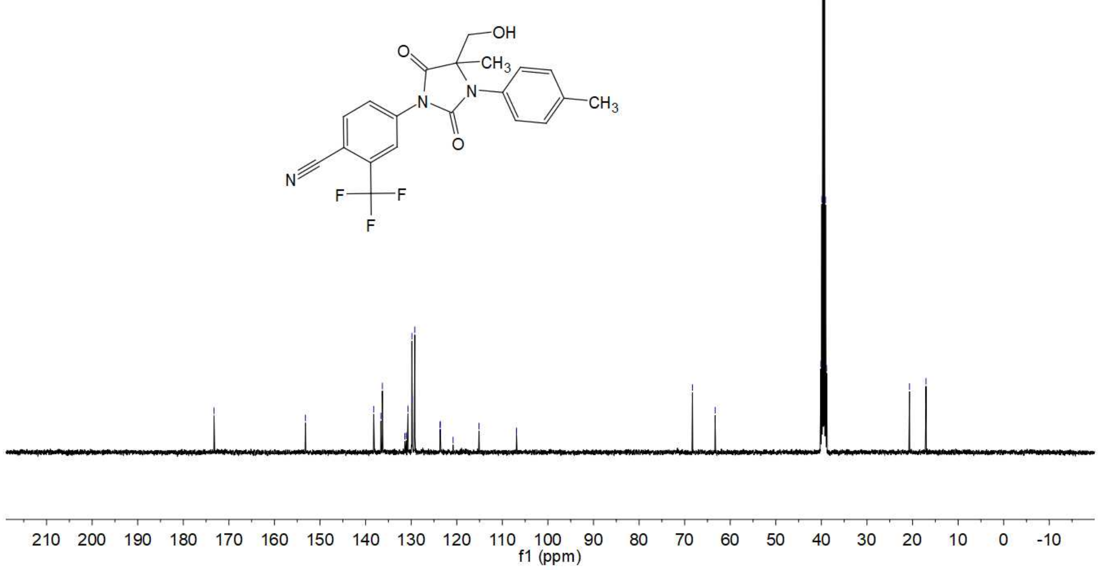

Elemental Composition Report

Single Mass Analysis

Tolerance = 500.0 PPM / DBE: min = -1.5, max = 50.0
Element prediction: Off

Monoisotopic Mass, Even Electron Ions
1 formula(e) evaluated with 1 results within limits (up to 50 closest results for each mass)
Elements Used:
C: 20-20 H: 17-17 N: 3-3 O: 3-3 F: 3-3
JJY-A00193-013 26 (0.537)
1: TOF MS ES+

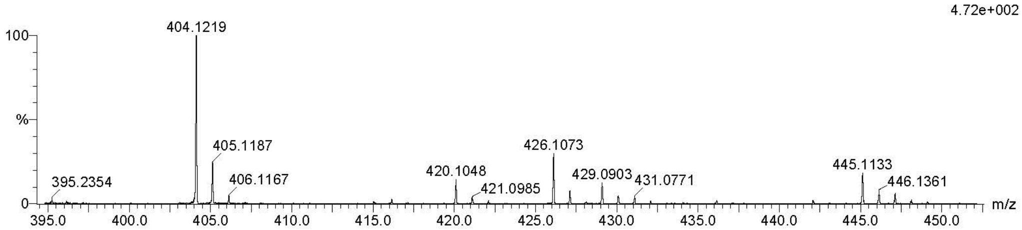

Minimum:				-1.5	
Maximum:		5.0	500.0	50.0	
Mass	Calc. Mass	mDa	PPM	DBE	Formula
404.1219	404.1222	-0.3	-0.7	12.5	C20 H17 N3 O3 F3

^{1}H NMR (400 MHz, DMSO) δ 8.36 (d, J = 8.4 Hz, 1H), 8.18 (s, 1H), 8.07 (d, J = 8.4 Hz, 1H), 7.50 (dd, J = 8.8, 5.1 Hz, 2H), 7.38 (t, J = 8.8 Hz, 2H), 5.73 (t, J = 5.2 Hz, 1H), 3.75 (dd, J = 11.7, 5.1 Hz, 1H), 3.36 (dd, J = 11.7, 5.4 Hz, 1H), 1.31 (s, 3H).

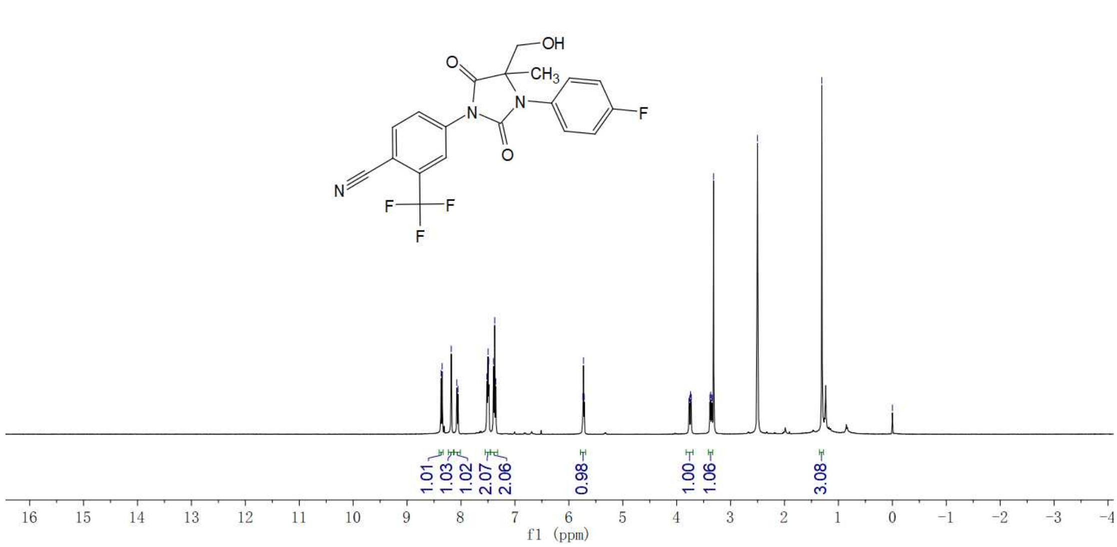

^{13}C NMR (101 MHz, DMSO) δ 173.13, 162.99, 160.55, 153.28, 136.53, 136.37, 131.70, 131.61, 131.44, 131.11, 129.79, 129.60, 123.67, 123.62, 120.81, 116.40, 116.18, 115.11, 107.00, 68.39, 63.27, 16.91.

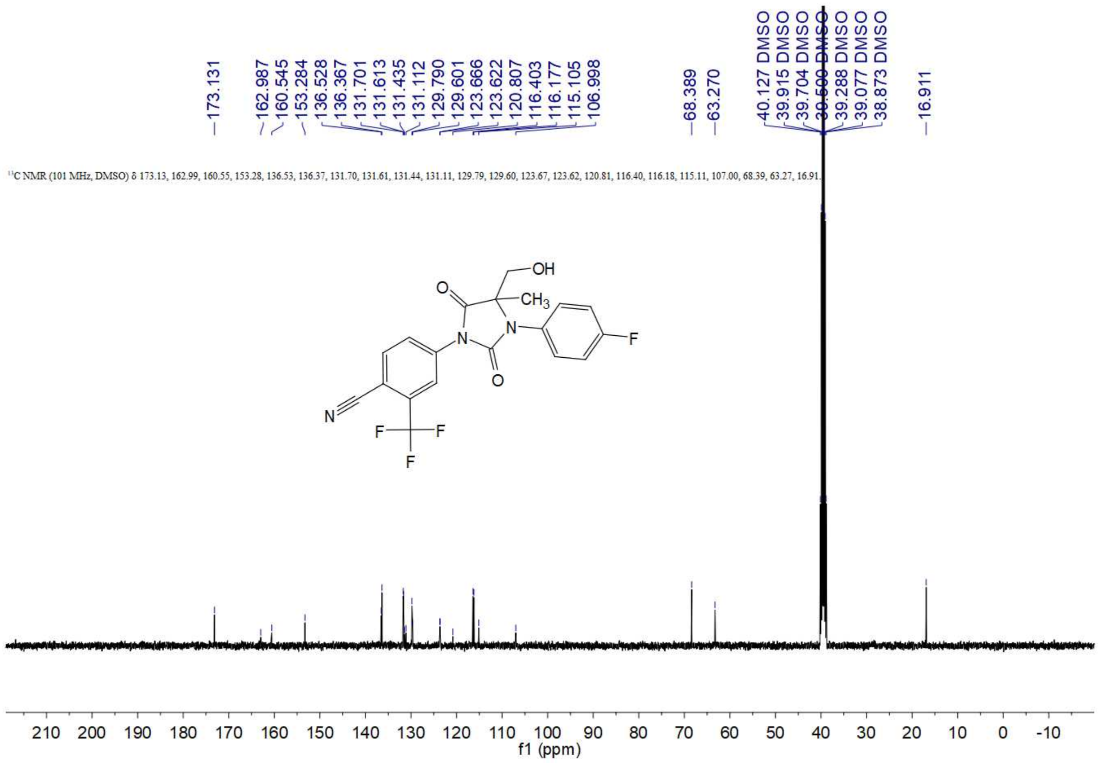

Elemental Composition Report

Single Mass Analysis

Tolerance = 500.0 PPM / DBE: min = -1.5, max = 50.0
Element prediction: Off

Monoisotopic Mass, Even Electron Ions
1 formula(e) evaluated with 1 results within limits (up to 50 closest results for each mass)
Elements Used:
C: 19-19 H: 14-14 N: 3-3 O: 3-3 F: 4-4
JJY-A00193-038 26 (0.537)
1: TOF MS ES+

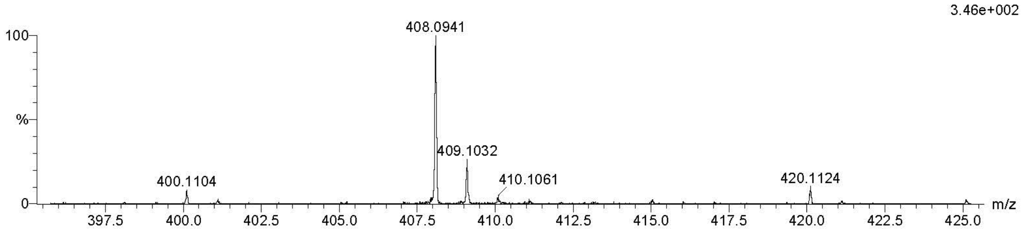

Minimum:				-1.5	
Maximum:		5.0	500.0	50.0	
Mass	Calc. Mass	mDa	PPM	DBE	Formula
408.0941	408.0971	-3.0	-7.4	12.5	C19 H14 N3 O3 F4

^{1}H NMR (400 MHz, DMSO) δ 8.36 (d, J = 8.4 Hz, 1H), 8.18 (d, J = 1.4 Hz, 1H), 8.06 (dd, J = 8.4, 1.8 Hz, 1H), 7.61 (d, J = 8.7 Hz, 2H), 7.48 (d, J = 8.6 Hz, 2H), 5.73 (t, J = 5.2 Hz, 1H), 3.76 (dd, J = 11.8, 5.1 Hz, 1H), 3.39 (dd, J = 11.8, 5.4 Hz, 1H), 1.32 (s, 3H).

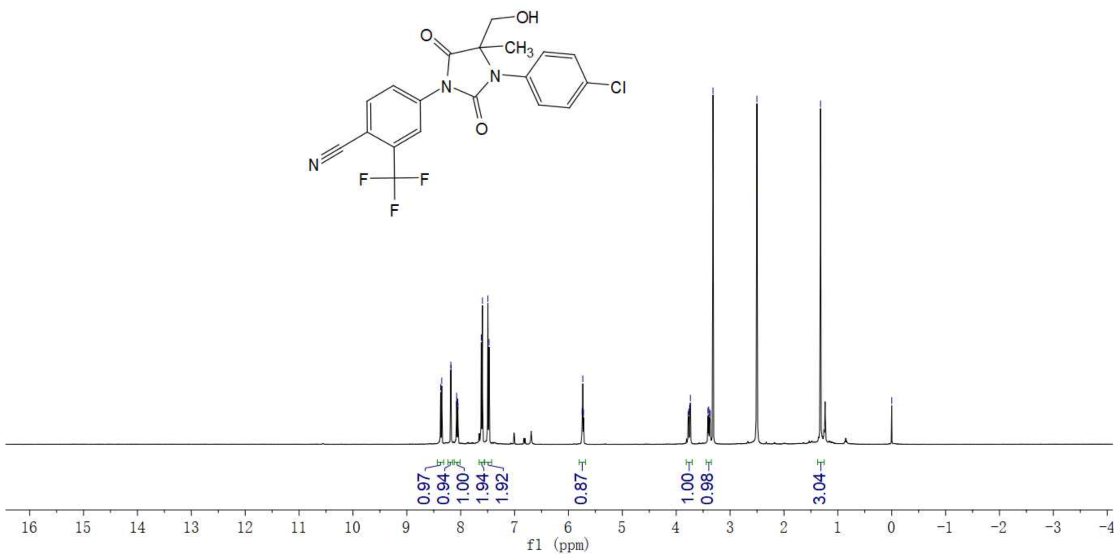

^{13}C NMR (101 MHz, DMSO) δ 173.07, 153.17, 136.46, 136.37, 136.32, 133.19, 132.47, 131.44, 131.07, 130.79, 129.86, 129.46, 123.72, 123.67, 123.52, 120.80, 118.07, 117.30, 115.54, 115.10, 110.74, 107.06, 68.53, 63.36, 16.96.

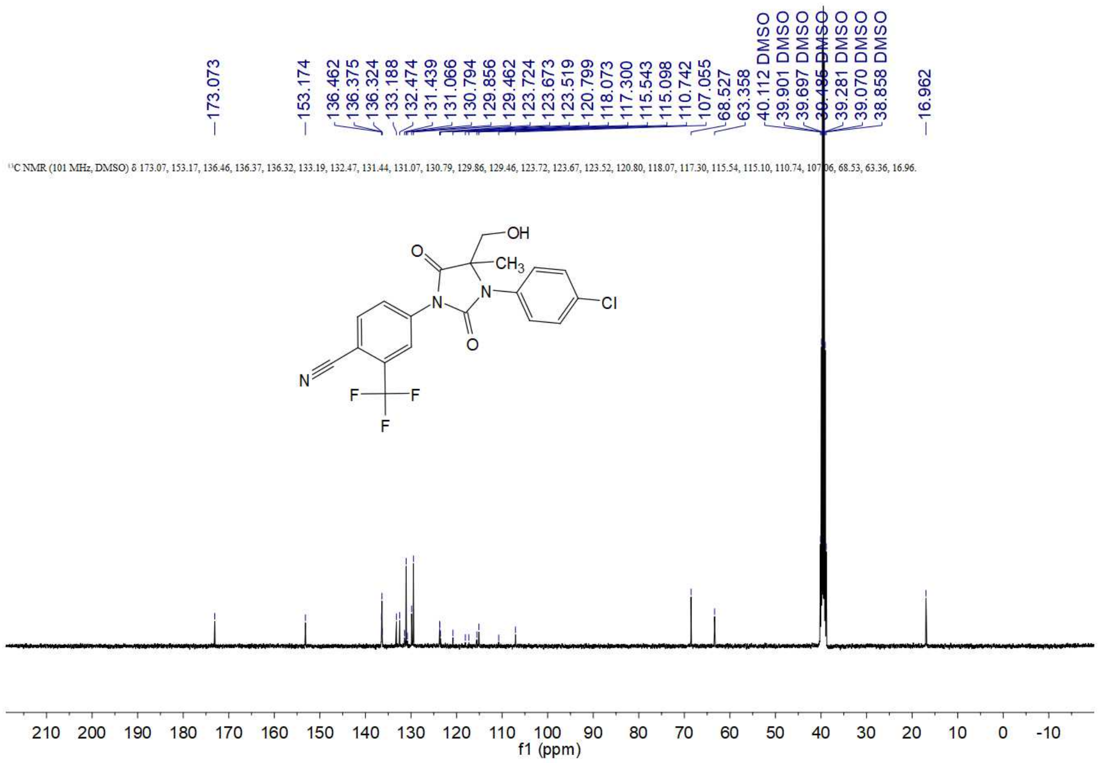

Elemental Composition Report

Single Mass Analysis
Tolerance = 500.0 PPM / DBE: min = -1.5, max = 50.0
Element prediction: Off

Monoisotopic Mass, Even Electron Ions
1 formula(e) evaluated with 1 results within limits (up to 50 closest results for each mass)
Elements Used:
C: 19-19 H: 14-14 N: 3-3 O: 3-3 F: 3-3 Cl: 1-1
JJY-A00193-055 27 (0.554)
1: TOF MS ES+

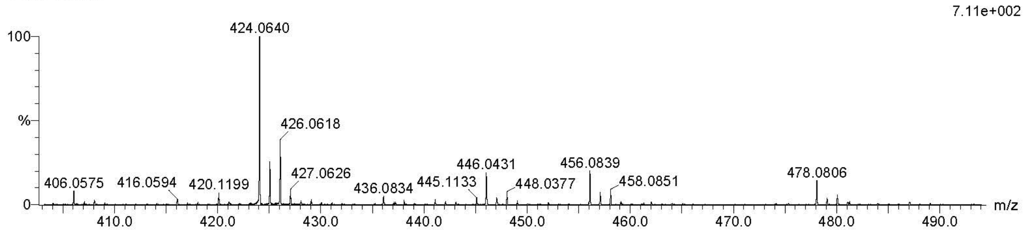

Minimum:				-1.5	
Maximum:		5.0	500.0	50.0	
Mass	Calc. Mass	mDa	PPM	DBE	Formula
424.0640	424.0676	-3.6	-8.5	12.5	C19 H14 N3 O3 F3 Cl

^{1}H NMR (400 MHz, DMSO) δ 8.35 (d, J = 5.6 Hz, 1H), 8.18 (s, 1H), 8.07 (d, J = 5.8 Hz, 1H), 7.37 (d, J = 5.0 Hz, 2H), 7.06 (d, J = 5.8 Hz, 2H), 5.69 (s, 1H), 3.81 (s, 3H), 3.73 (s, 1H), 3.33 (s, 1H), 1.29 (s, 3H).

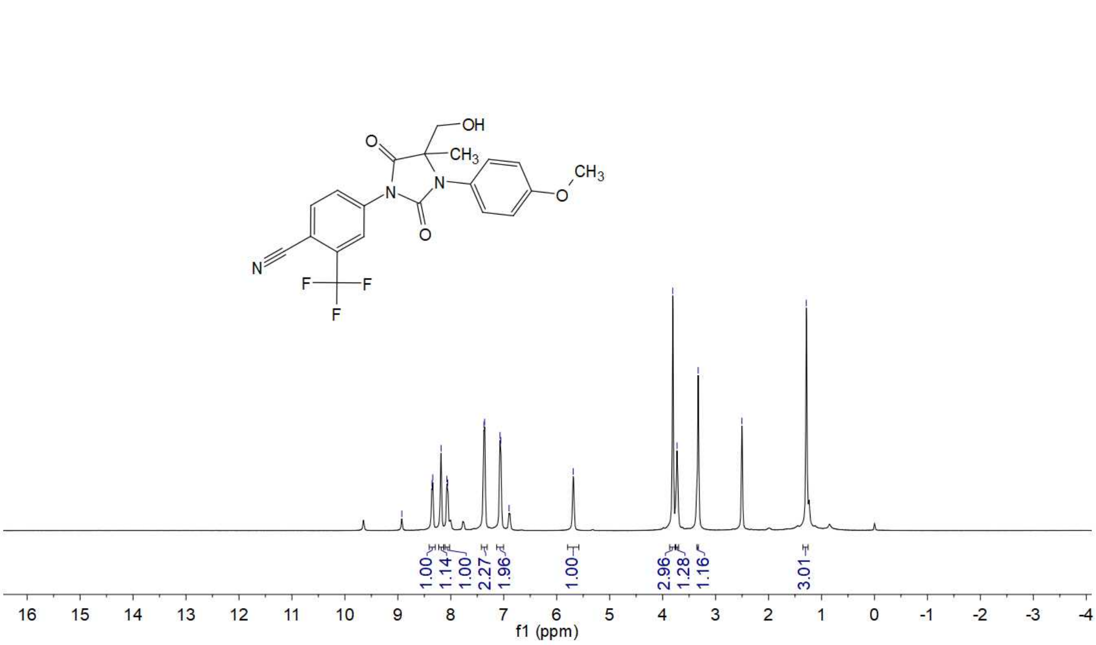

^{13}C NMR (101 MHz, DMSO) δ 173.28, 159.26, 153.33, 136.66, 136.32, 130.78, 129.73, 125.59, 123.65, 123.62, 123.57, 123.53, 120.81, 120.74, 120.65, 115.13, 114.54, 113.98, 106.86, 68.21, 63.22, 55.34, 16.95.

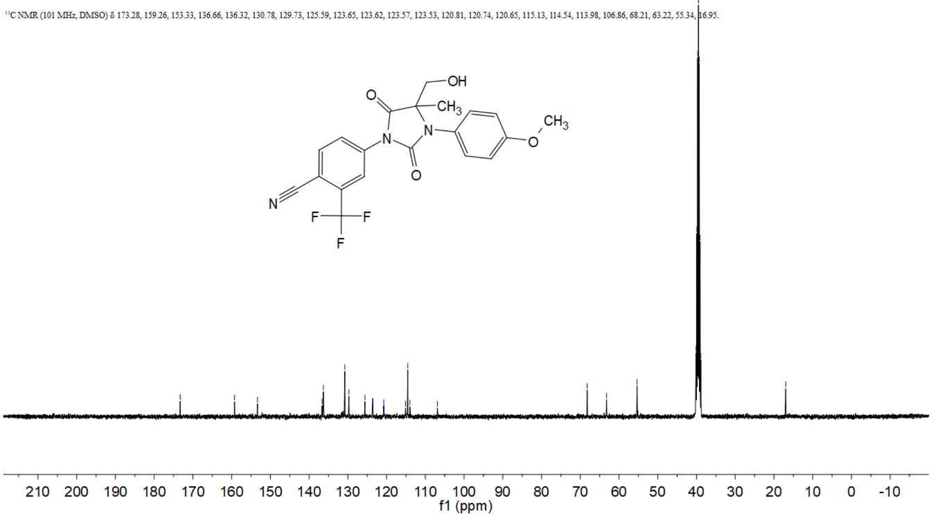

Elemental Composition Report

Single Mass Analysis

Tolerance = 500.0 PPM / DBE: min = -1.5, max = 50.0
Element prediction: Off

Monoisotopic Mass, Even Electron Ions
1 formula(e) evaluated with 1 results within limits (up to 50 closest results for each mass)
Elements Used:
C: 20-20 H: 17-17 N: 3-3 O: 4-4 F: 3-3
JJY-A00193-042 18 (0.382)
1: TOF MS ES+

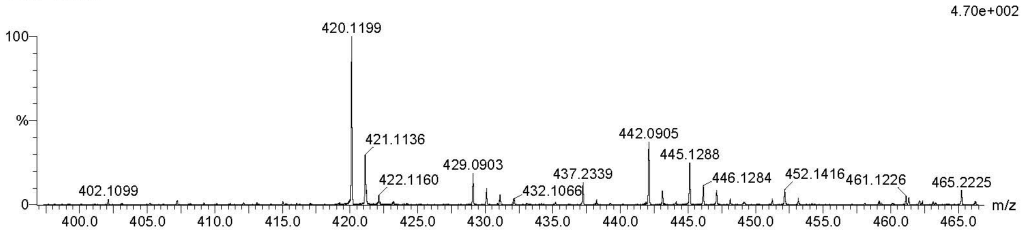

Minimum:				-1.5	
Maximum:		5.0	500.0	50.0	
Mass	Calc. Mass	mDa	PPM	DBE	Formula
420.1199	420.1171	2.8	6.7	12.5	C20 H17 N3 O4 F3

^{1}H NMR (400 MHz, DMSO) δ 8.50 – 8.30 (m, 3H), 8.20 (d, *J* = 1.0 Hz, 1H), 8.07 (dd, *J* = 8.4, 1.4 Hz, 1H), 7.78 (d, *J* = 8.9 Hz, 2H), 5.80 (t, *J* = 5.3 Hz, 1H), 3.82 (dd, *J* = 11.9, 5.3 Hz, 1H), 3.56 (dd, *J* = 11.9, 5.5 Hz, 1H), 1.42 (s, 3H).

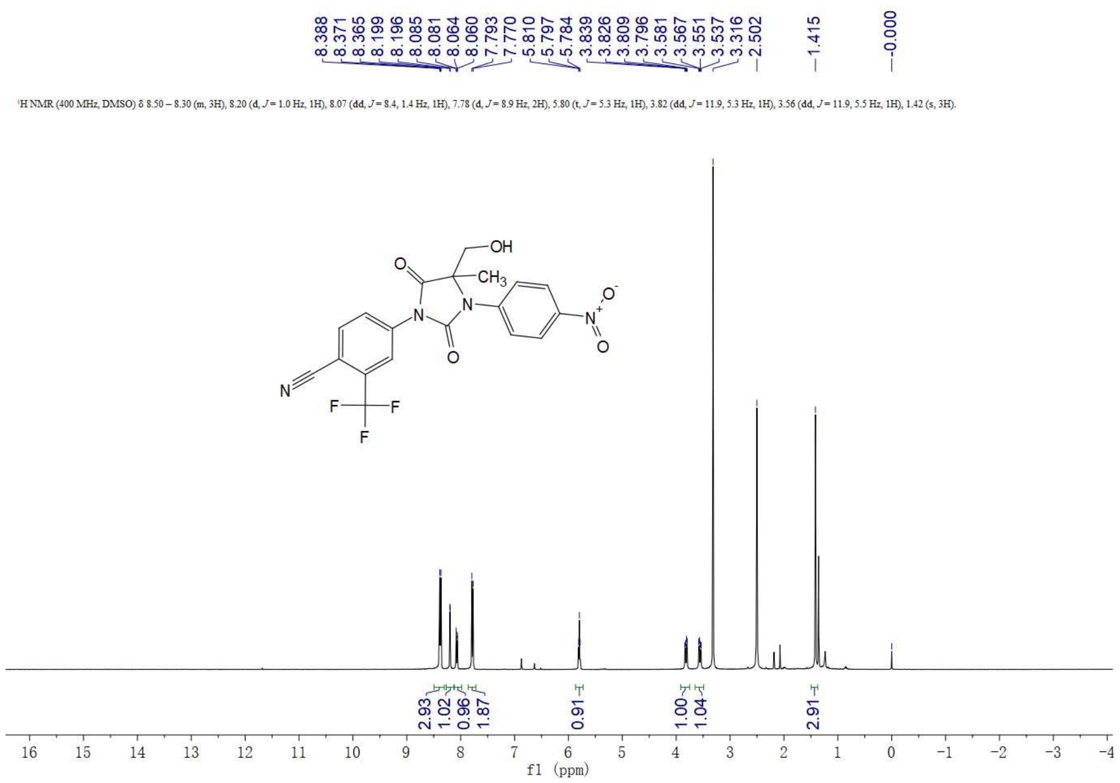

^{13}C NMR (101 MHz, DMSO) δ 172.82, 153.09, 146.28, 140.32, 136.42, 136.22, 131.81, 131.50, 131.18, 130.85, 130.11, 128.80, 124.64, 123.96, 123.92, 123.50, 120.78, 115.05, 107.36, 69.14, 63.70, 30.36, 17.13.

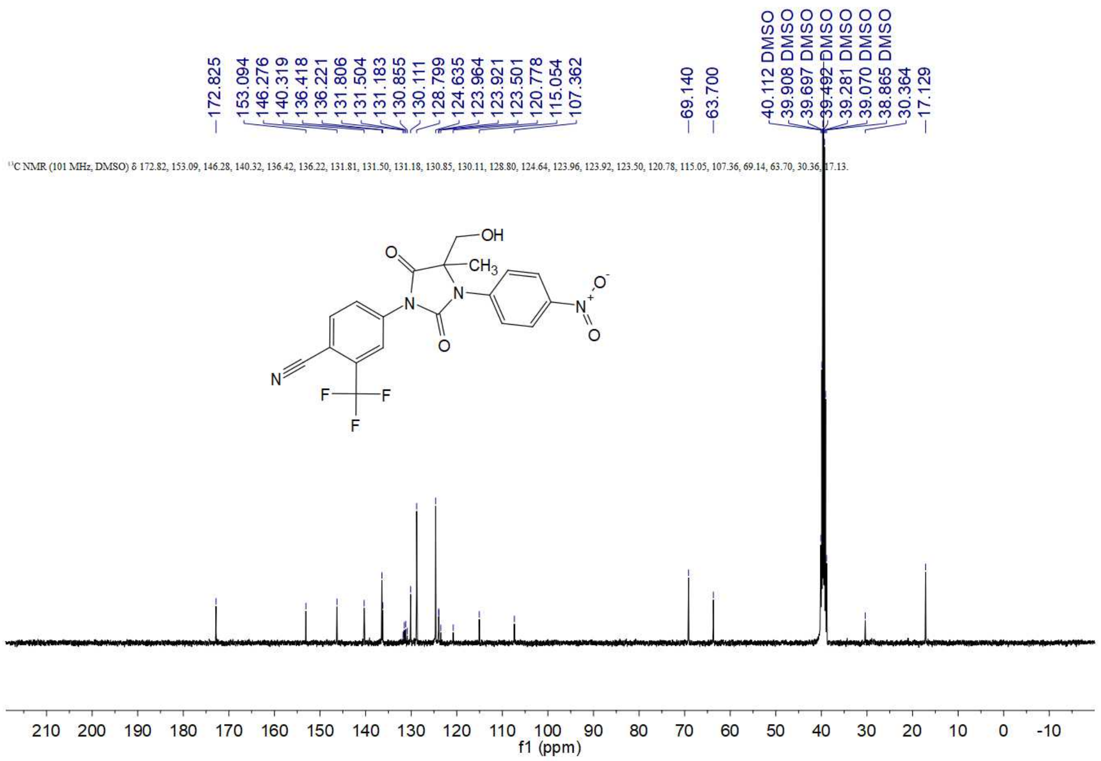

Single Mass Analysis

Tolerance = 500.0 PPM / DBE: min = -1.5, max = 50.0
Element prediction: Off

Monoisotopic Mass, Even Electron Ions
1 formula(e) evaluated with 1 results within limits (up to 50 closest results for each mass)
Elements Used:
C: 19-19 H: 13-13 N: 4-4 O: 5-5 F: 3-3 Na: 0-1

JJY-A00193-085-2 29 (0.588)
1: TOF MS ES+

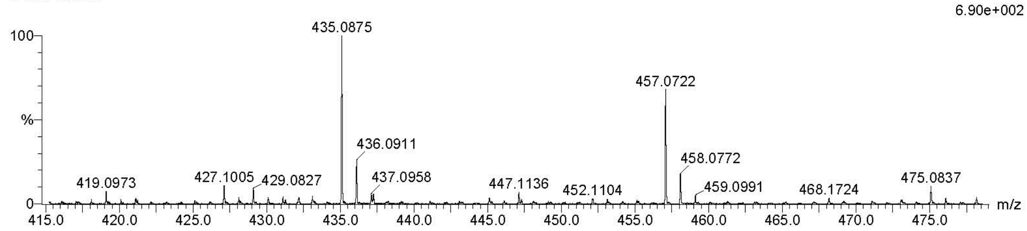

	Calc. Mass	mDa	PPM	DBE	Formula
Minimum:				-1.5	
Maximum:		5.0	500.0	50.0	
Mass	Calc. Mass	mDa	PPM	DBE	Formula
457.0722	457.0736	-1.4	-3.1	13.5	C19 H13 N4 O5 F3 Na

^{1}H NMR (400 MHz, DMSO) δ 8.37 (d, J = 8.4 Hz, 1H), 8.19 (d, J = 1.6 Hz, 1H), 8.07 (dd, J = 8.4, 1.6 Hz, 1H), 7.92 (d, J = 8.4 Hz, 2H), 7.71 (d, J = 8.3 Hz, 2H), 5.77 (t, J = 5.3 Hz, 1H), 3.79 (dd, J = 11.8, 5.2 Hz, 1H), 3.47 (dd, J = 11.8, 5.5 Hz, 1H), 1.37 (s, 3H).

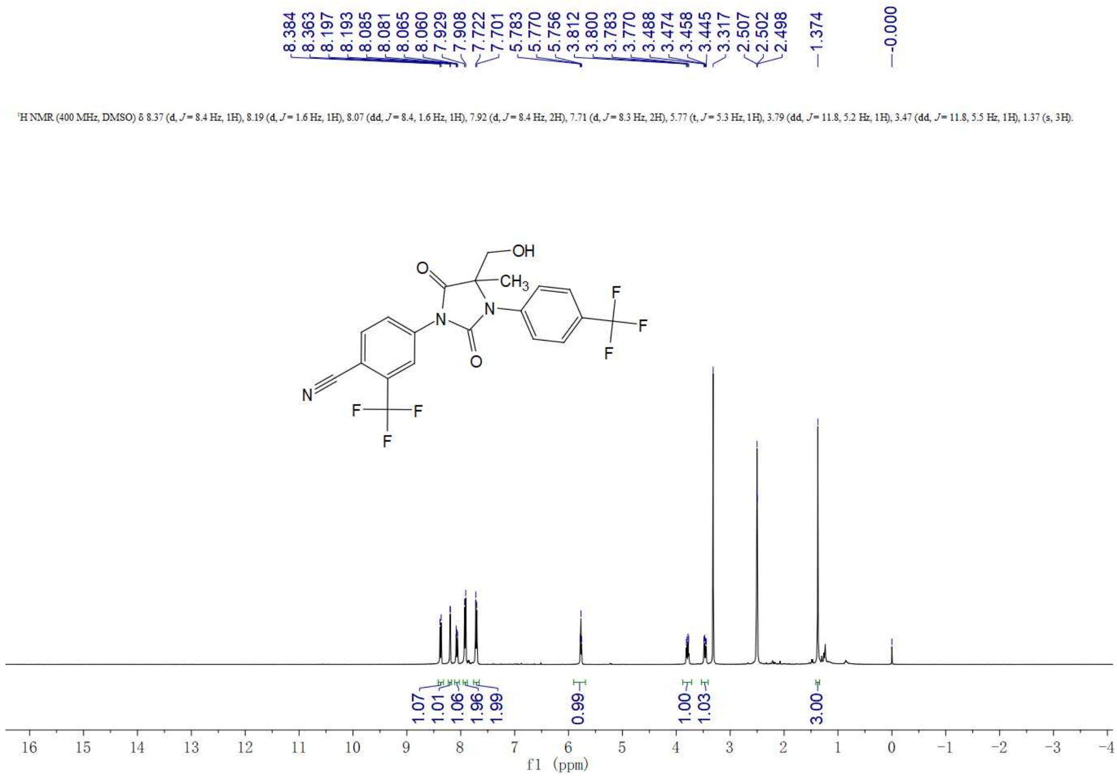

^{13}C NMR (101 MHz, DMSO) δ 172.96, 153.18, 137.72, 136.38, 131.48, 131.15, 129.97, 129.51, 128.70, 128.38, 126.49, 126.46, 125.28, 123.83, 123.79, 123.51, 122.58, 120.79, 115.07, 107.21, 68.82, 63.53, 17.09.

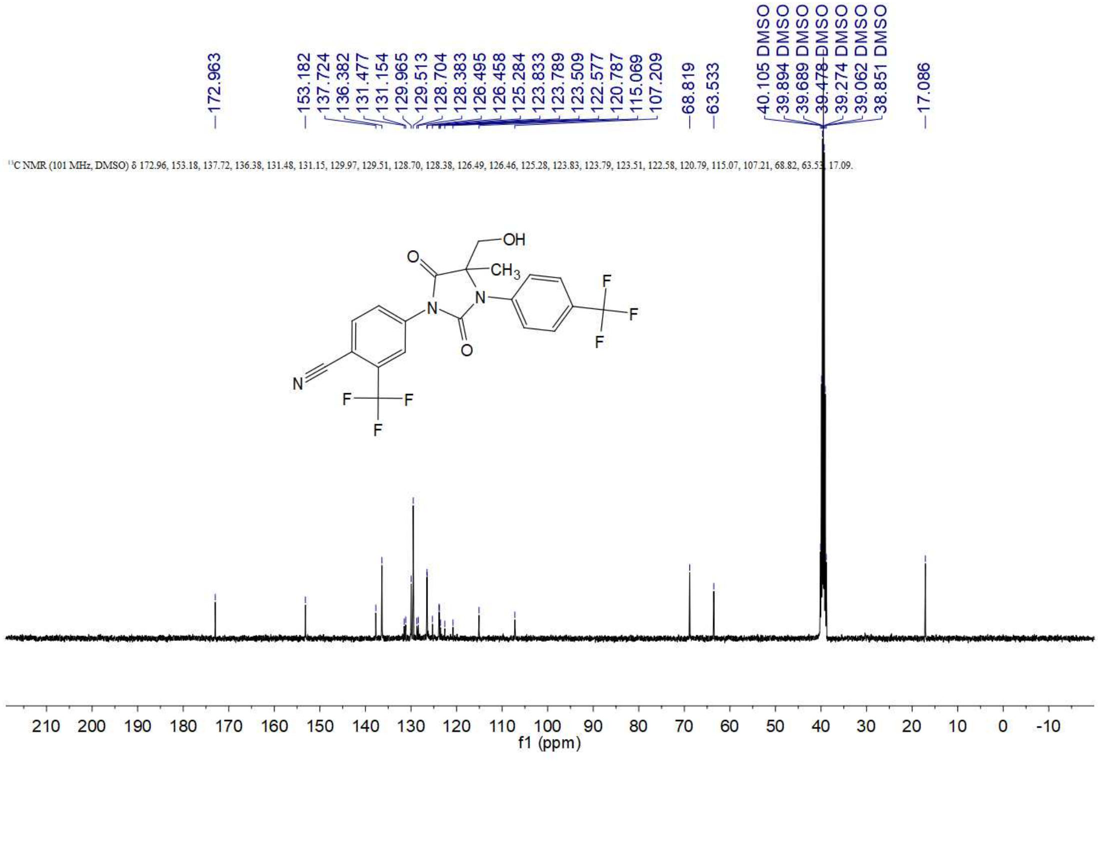

Elemental Composition Report

Single Mass Analysis
Tolerance = 500.0 PPM / DBE: min = -1.5, max = 50.0
Element prediction: Off

Monoisotopic Mass, Even Electron Ions
1 formula(e) evaluated with 1 results within limits (up to 50 closest results for each mass)
Elements Used:
C: 20-20 H: 14-14 N: 3-3 O: 3-3 F: 6-6
JJY-A00193-089 28 (0.571)
1: TOF MS ES+

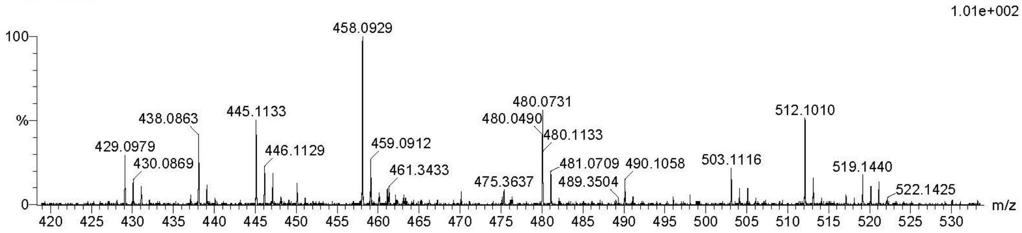

Minimum:				-1.5	
Maximum:		5.0	500.0	50.0	
Mass	Calc. Mass	mDa	PPM	DBE	Formula
458.0929	458.0939	-1.0	-2.2	12.5	C20 H14 N3 O3 F6

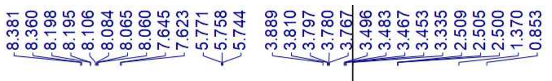

^{1}H NMR (400 MHz, DMSO) δ 8.37 (d, J = 8.4 Hz, 1H), 8.20 (d, J = 1.5 Hz, 1H), 8.08 (dd, J = 13.0, 5.1 Hz, 3H), 7.63 (d, J = 8.6 Hz, 2H), 5.76 (t, J = 5.3 Hz, 1H), 3.89 (s, 3H), 3.79 (dd, J = 11.8, 5.2 Hz, 1H), 3.47 (dd, J = 11.8, 5.4 Hz, 1H), 1.37 (s, 3H).

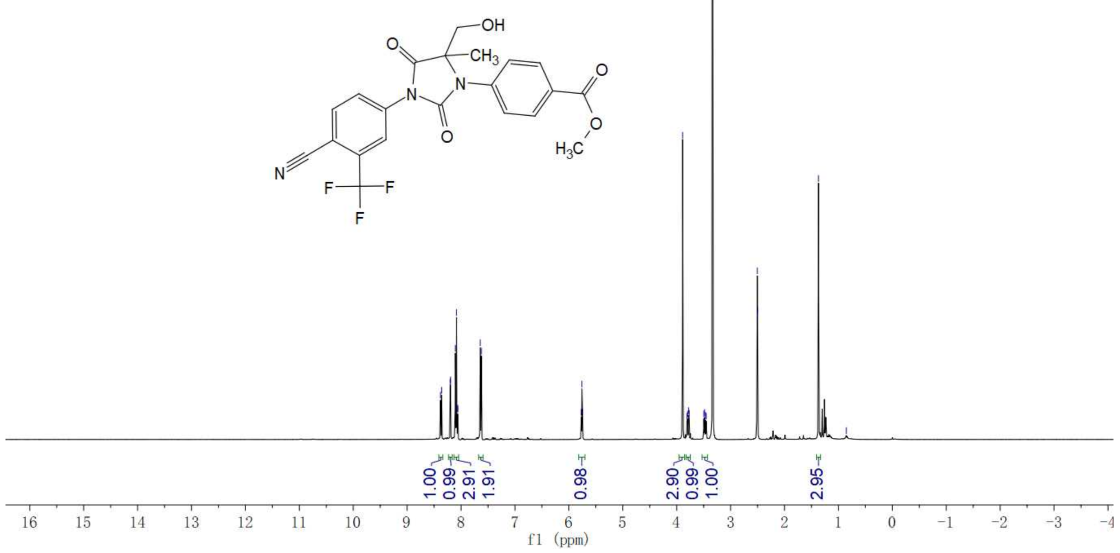

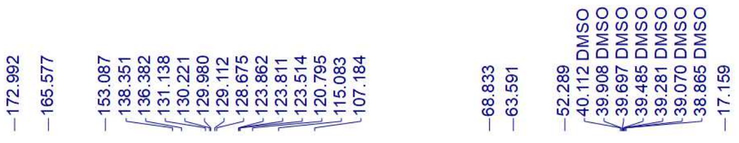

^{13}C NMR (101 MHz, DMSO) δ 172.99, 165.58, 153.09, 138.35, 136.38, 131.14, 130.22, 129.98, 129.11, 128.67, 123.86, 123.81, 123.51, 120.79, 115.08, 107.18, 68.83, 63.59, 52.29, 17.16.

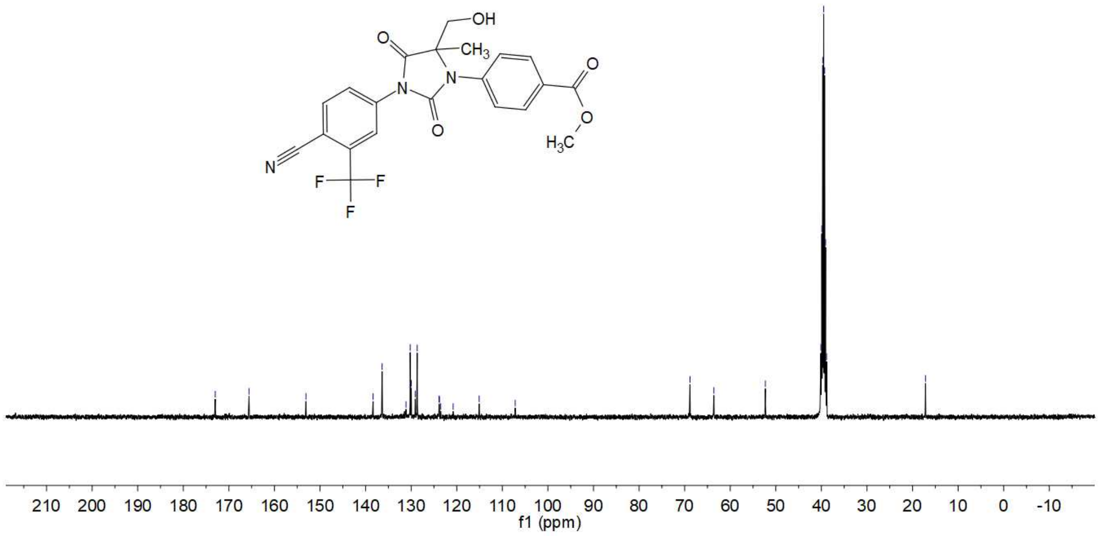

Elemental Composition Report

Single Mass Analysis

Tolerance = 500.0 PPM / DBE: min = -1.5, max = 50.0
Element prediction: Off

Monoisotopic Mass, Even Electron Ions
1 formula(e) evaluated with 1 results within limits (up to 50 closest results for each mass)
Elements Used:
C: 21-21 H: 17-17 N: 3-3 O: 5-5 F: 3-3
JJY-A00193-092 18 (0.382)
1: TOF MS ES+

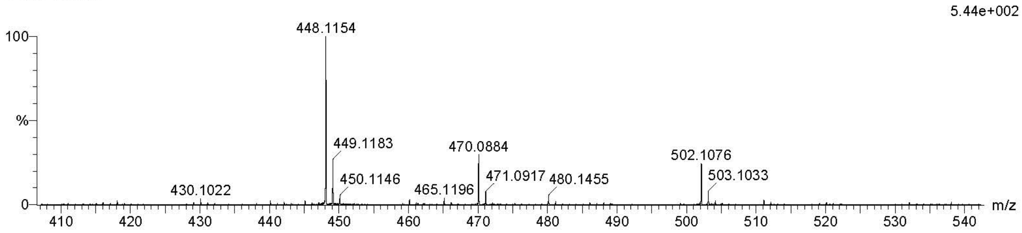

Minimum:				-1.5	
Maximum:		5.0	500.0	50.0	
Mass	Calc. Mass	mDa	PPM	DBE	Formula
448.1154	448.1120	3.4	7.6	13.5	C21 H17 N3 O5 F3

^{1}H, ^{13}C, NMR and HRMS spectra of *compound* **9**

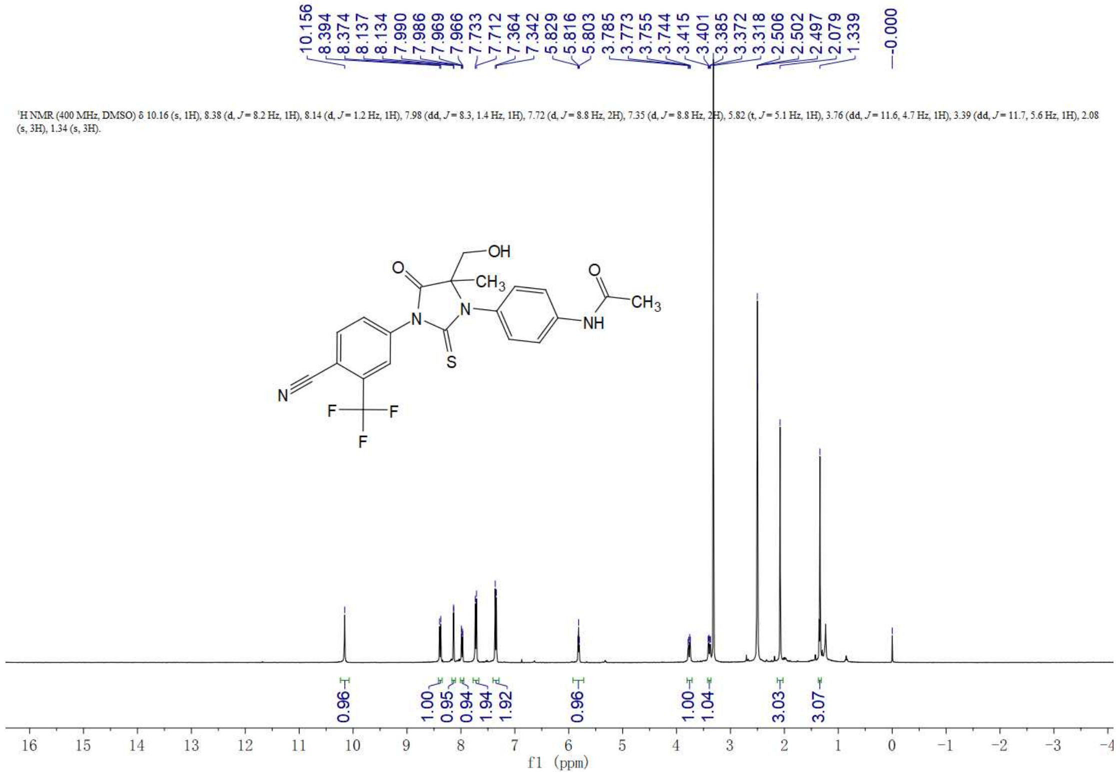

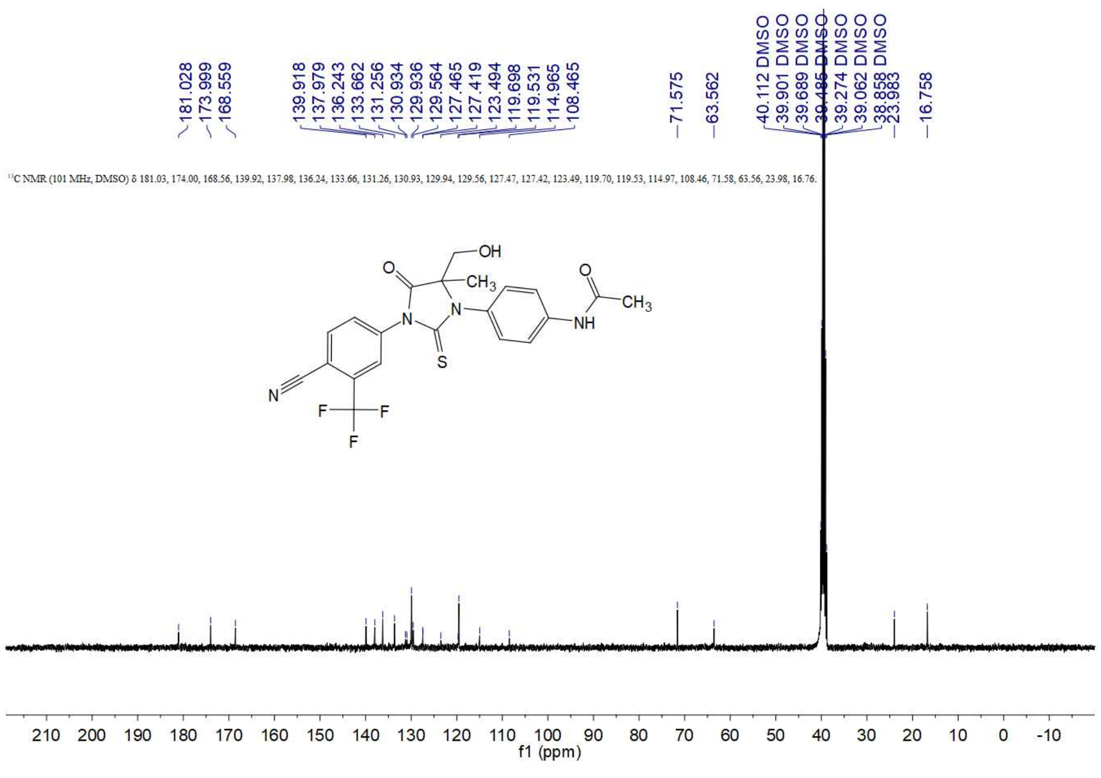

Elemental Composition Report

Single Mass Analysis
Tolerance = 500.0 PPM / DBE: min = -1.5, max = 50.0
Element prediction: Off

Monoisotopic Mass, Even Electron Ions
1 formula(e) evaluated with 1 results within limits (up to 50 closest results for each mass)
Elements Used:
C: 21-21 H: 17-17 N: 4-4 O: 3-3 F: 3-3 Na: 0-1 S: 1-1
JJY-A00193-088 35 (0.710)
1: TOF MS ES+

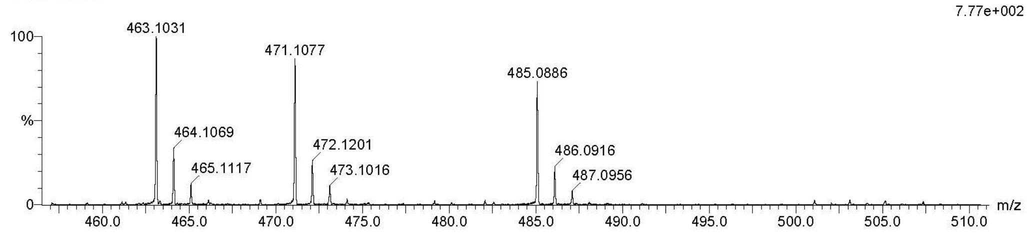

Minimum:				-1.5	
Maximum:		5.0	500.0	50.0	
Mass	Calc. Mass	mDa	PPM	DBE	Formula
485.0886	485.0871	1.5	3.1	13.5	C21 H17 N4 O3 F3 Na S

^{1}H, ^{13}C, NMR and HRMS spectra of *compound* **10**

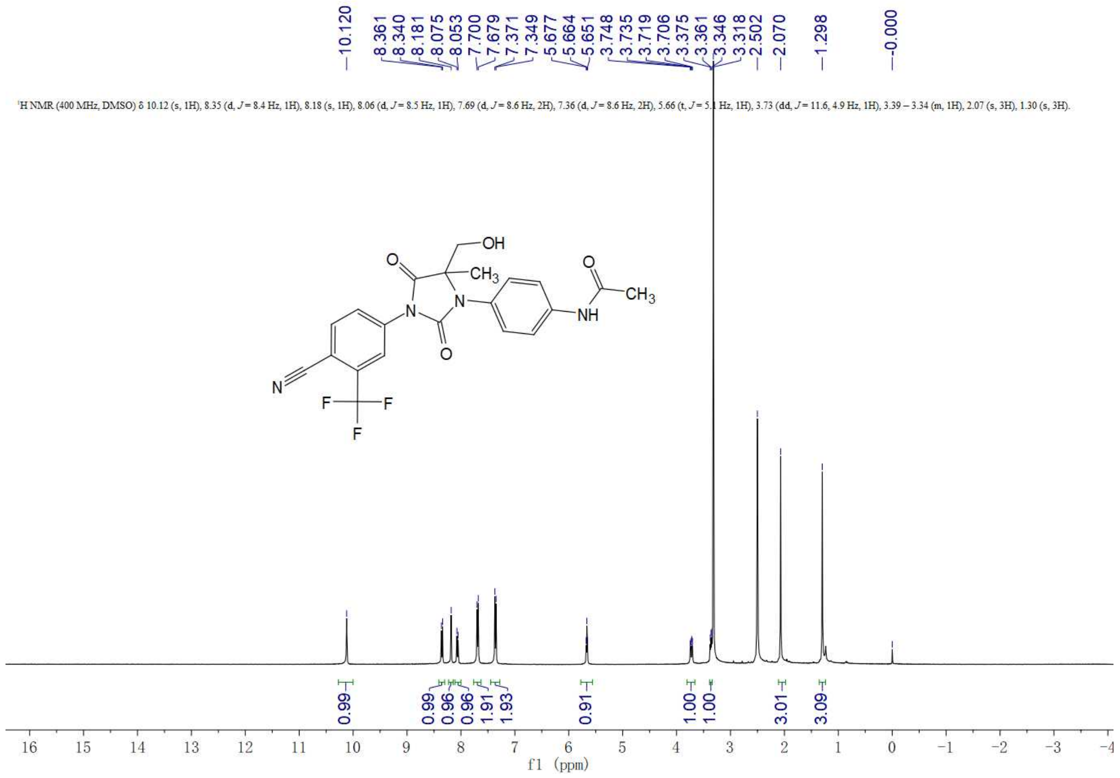

^{1}H NMR (400 MHz, DMSO) δ 10.12 (s, 1H), 8.35 (d, *J* = 8.4 Hz, 1H), 8.18 (s, 1H), 8.06 (d, *J* = 8.5 Hz, 1H), 7.69 (d, *J* = 8.6 Hz, 2H), 7.36 (d, *J* = 8.6 Hz, 2H), 5.66 (t, *J* = 5.1 Hz, 1H), 3.73 (dd, *J* = 11.6, 4.9 Hz, 1H), 3.39 – 3.34 (m, 1H), 2.07 (s, 3H), 1.30 (s, 3H).

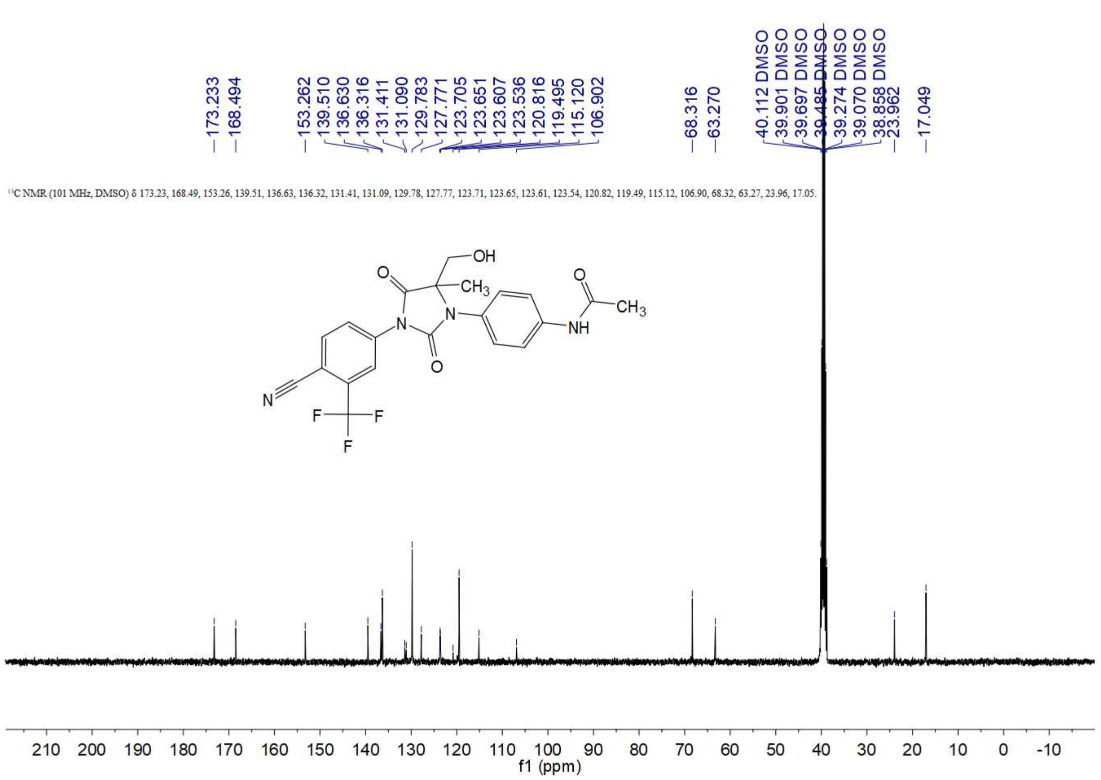

^{13}C NMR (101 MHz, DMSO) δ 173.23, 168.49, 153.26, 139.51, 136.63, 136.32, 131.41, 131.09, 129.78, 127.77, 123.71, 123.65, 123.61, 123.54, 120.82, 119.49, 115.12, 106.90, 68.32, 63.27, 23.96, 17.05.

Elemental Composition Report

Single Mass Analysis

Tolerance = 500.0 PPM / DBE: min = -1.5, max = 50.0
Element prediction: Off

Monoisotopic Mass, Even Electron Ions
1 formula(e) evaluated with 1 results within limits (up to 50 closest results for each mass)
Elements Used:
C: 21-21 H: 17-17 N: 4-4 O: 4-4 F: 3-3 Na: 0-1
JJY-A00193-101 42 (0.849)
1: TOF MS ES+

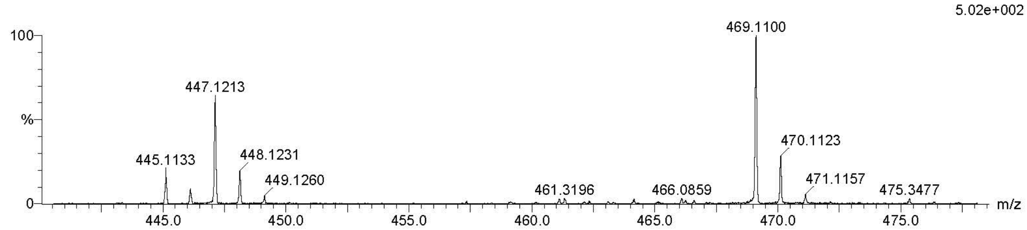

Minimum:				-1.5	
Maximum:		5.0	500.0	50.0	
Mass	Calc. Mass	mDa	PPM	DBE	Formula
469.1100	469.1100	0.0	0.0	13.5	C21 H17 N4 O4 F3 Na

^{1}H, ^{13}C, NMR and HRMS spectra of *compound* **11a-b**

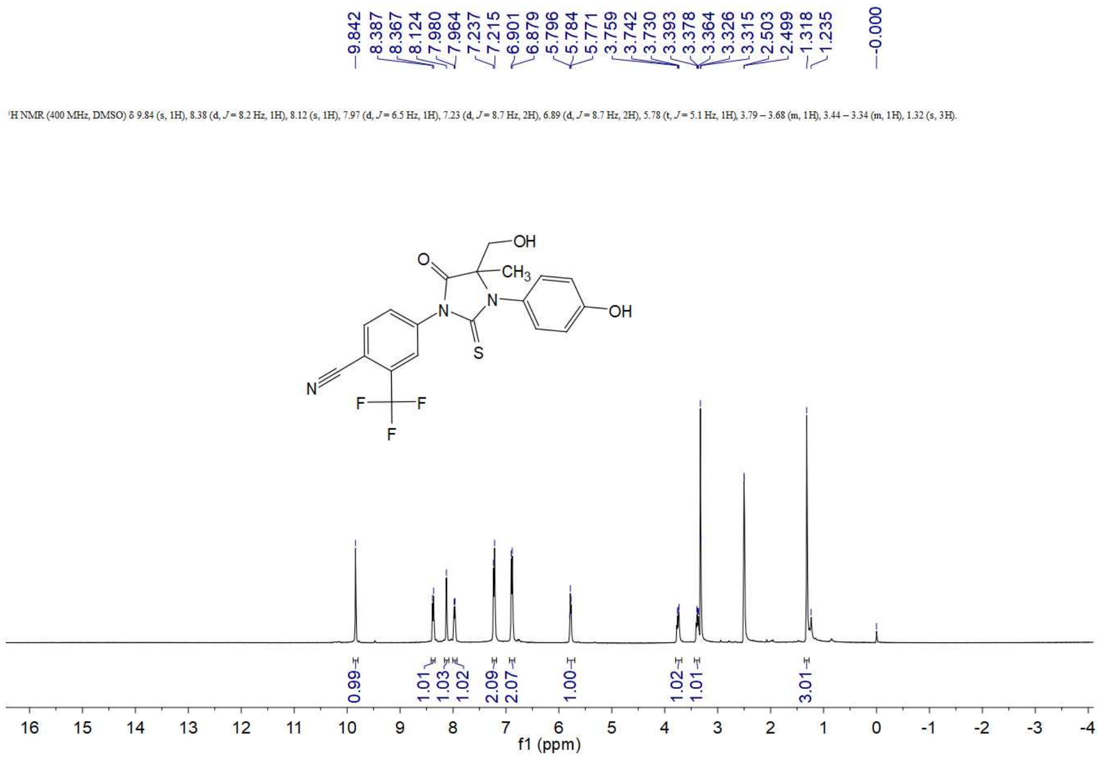

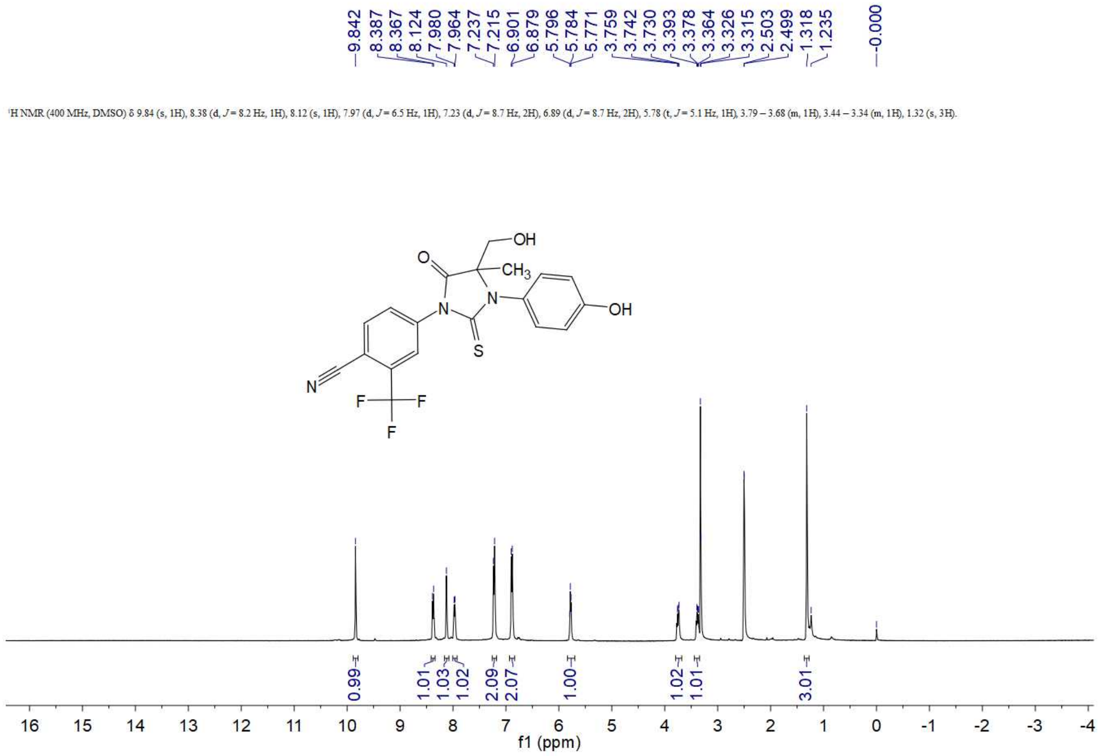
^{1}H NMR (400 MHz, DMSO) δ 9.84 (s, 1H), 8.38 (d, J = 8.2 Hz, 1H), 8.12 (s, 1H), 7.97 (d, J = 6.5 Hz, 1H), 7.23 (d, J = 8.7 Hz, 2H), 6.89 (d, J = 8.7 Hz, 2H), 5.78 (t, J = 5.1 Hz, 1H), 3.79 – 3.68 (m, 1H), 3.44 – 3.34 (m, 1H), 1.32 (s, 3H).

Single Mass Analysis

Tolerance = 500.0 PPM / DBE: min = -1.5, max = 50.0
Element prediction: Off

Monoisotopic Mass, Even Electron Ions
1 formula(e) evaluated with 1 results within limits (up to 50 closest results for each mass)
Elements Used:
C: 19-19 H: 15-15 N: 3-3 O: 3-3 F: 3-3 S: 1-1
JJY-A00193-095 31 (0.622)
1: TOF MS ES+

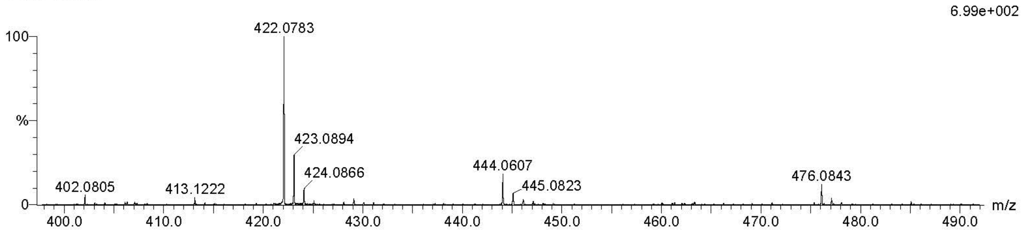

Minimum:				-1.5	
Maximum:		5.0	500.0	50.0	
Mass	Calc. Mass	mDa	PPM	DBE	Formula
422.0783	422.0786	-0.3	-0.7	12.5	C19 H15 N3 O3 F3 S

^{1}H NMR (400 MHz, DMSO) δ 9.77 (s, 1H), 8.34 (d, J = 8.4 Hz, 1H), 8.17 (s, 1H), 8.06 (d, J = 8.5 Hz, 1H), 7.23 (d, J = 8.8 Hz, 2H), 6.86 (d, J = 8.8 Hz, 2H), 5.63 (t, J = 5.1 Hz, 1H), 3.71 (dd, J = 11.5, 4.9 Hz, 1H), 3.34 (d, J = 5.4 Hz, 1H), 1.27 (s, 3H).

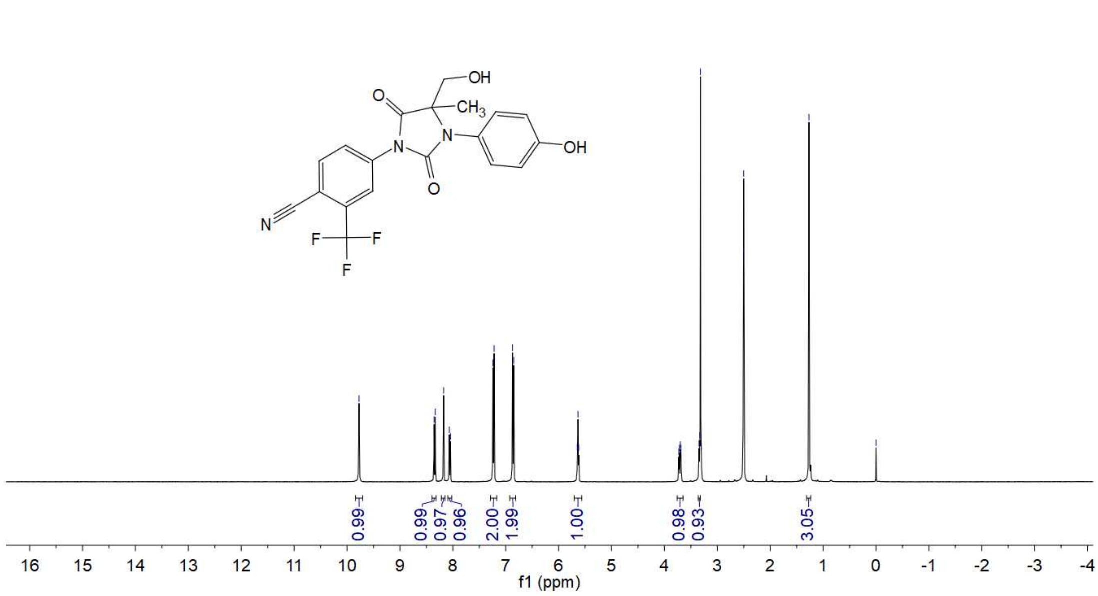

^{13}C NMR (101 MHz, DMSO) δ 173.36, 157.63, 153.33, 136.65, 136.29, 131.40, 131.08, 130.80, 129.75, 123.91, 123.65, 123.61, 123.52, 120.80, 115.80, 115.14, 106.81, 68.13, 63.12, 16.95.

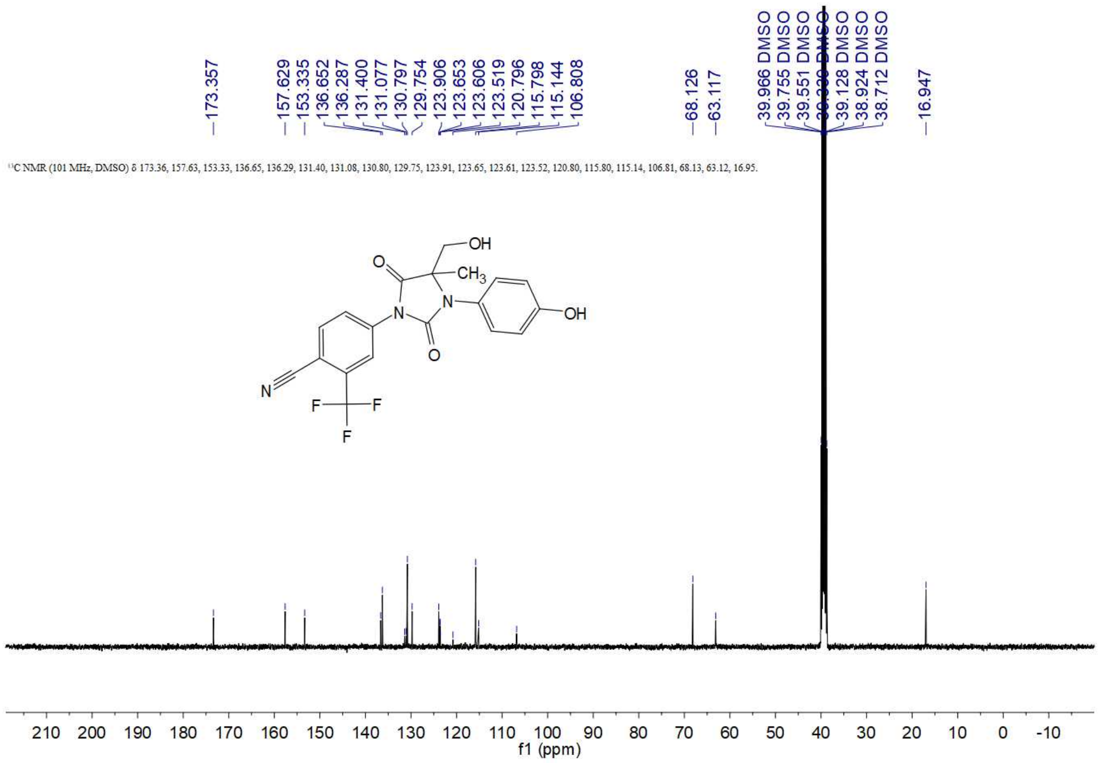

Elemental Composition Report

Single Mass Analysis
Tolerance = 500.0 PPM / DBE: min = -1.5, max = 50.0
Element prediction: Off

Monoisotopic Mass, Even Electron Ions
1 formula(e) evaluated with 1 results within limits (up to 50 closest results for each mass)
Elements Used:
C: 19-19 H: 15-15 N: 3-3 O: 4-4 F: 3-3
JJY-A00193-100 36 (0.727)
1: TOF MS ES+

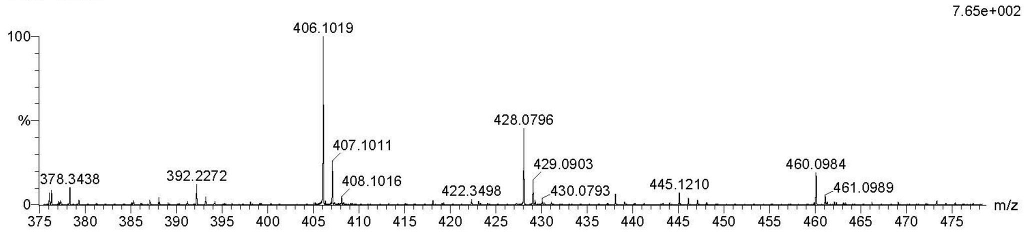

Mass	Calc. Mass	mDa	PPM	DBE	Formula
Minimum:				-1.5	
Maximum:		5.0	500.0	50.0	
406.1019	406.1015	0.4	1.0	12.5	C19 H15 N3 O4 F3

^{1}H ^{13}C, NMR and HRMS spectra spectra of *compound* **19a-d**

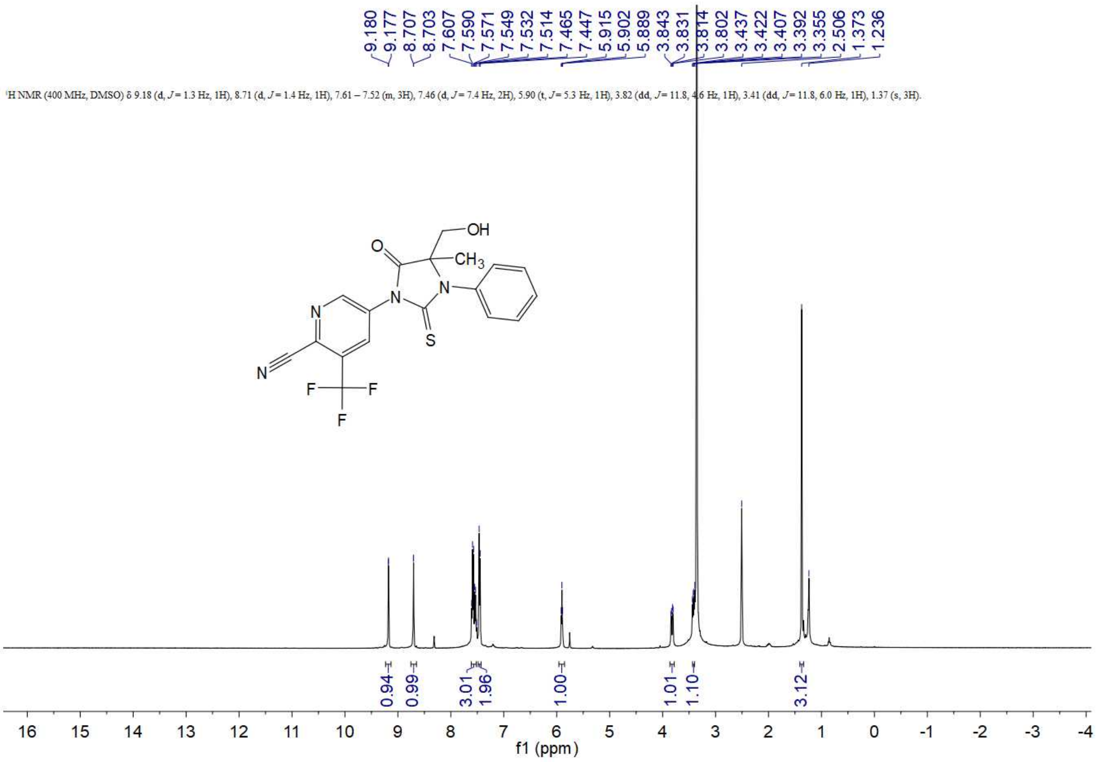

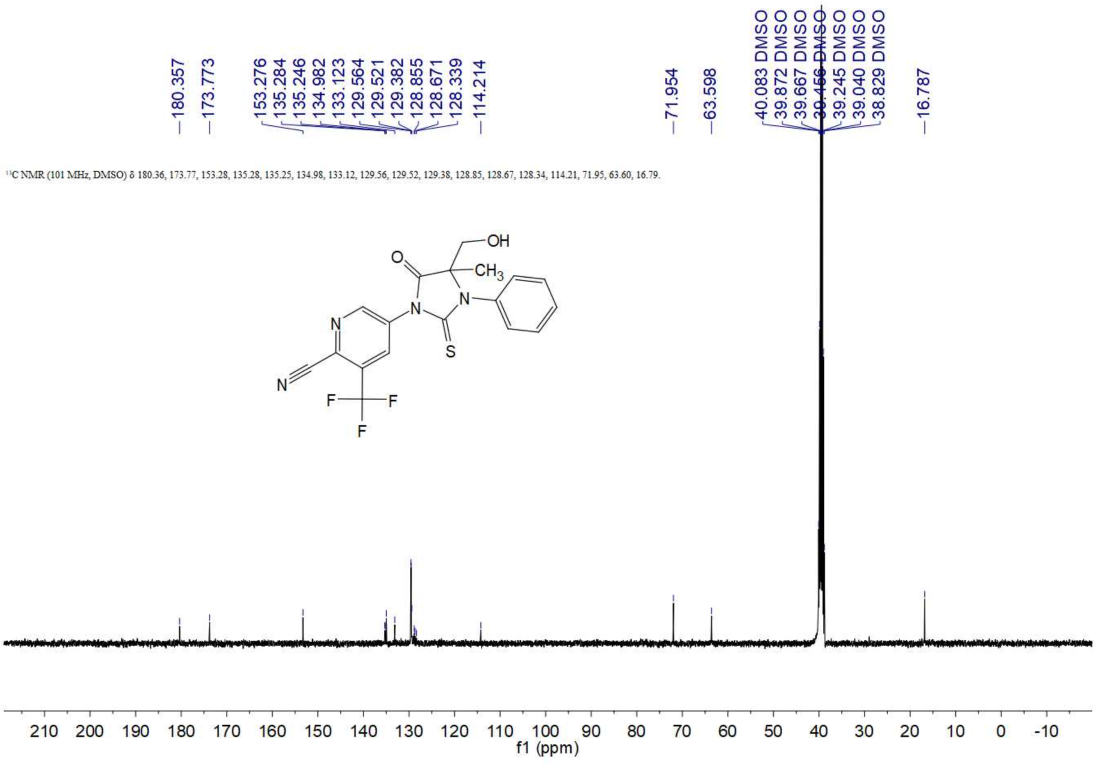

Elemental Composition Report

Single Mass Analysis
Tolerance = 500.0 PPM / DBE: min = -1.5, max = 50.0
Element prediction: Off

Monoisotopic Mass, Even Electron Ions
1 formula(e) evaluated with 1 results within limits (up to 50 closest results for each mass)
Elements Used:
C: 18-18 H: 14-14 N: 4-4 O: 2-2 F: 3-3 S: 1-1
JJY-A00222-062 35 (0.710)
1: TOF MS ES+

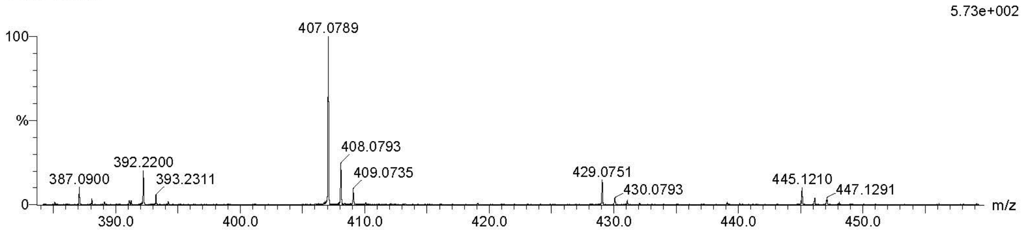

Minimum:				-1.5	
Maximum:		5.0	500.0	50.0	
Mass	Calc. Mass	mDa	PPM	DBE	Formula
407.0789	407.0790	-0.1	-0.2	12.5	C18 H14 N4 O2 F3 S

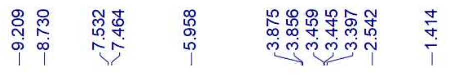

^{1}H NMR (400 MHz, DMSO) δ 9.21 (s, 1H), 8.73 (s, 1H), 7.53 (s, 2H), 7.46 (s, 2H), 5.96 (s, 1H), 3.87 (d, *J* = 7.7 Hz, 1H), 3.45 (d, *J* = 5.5 Hz, 1H), 1.41 (s, 3H).

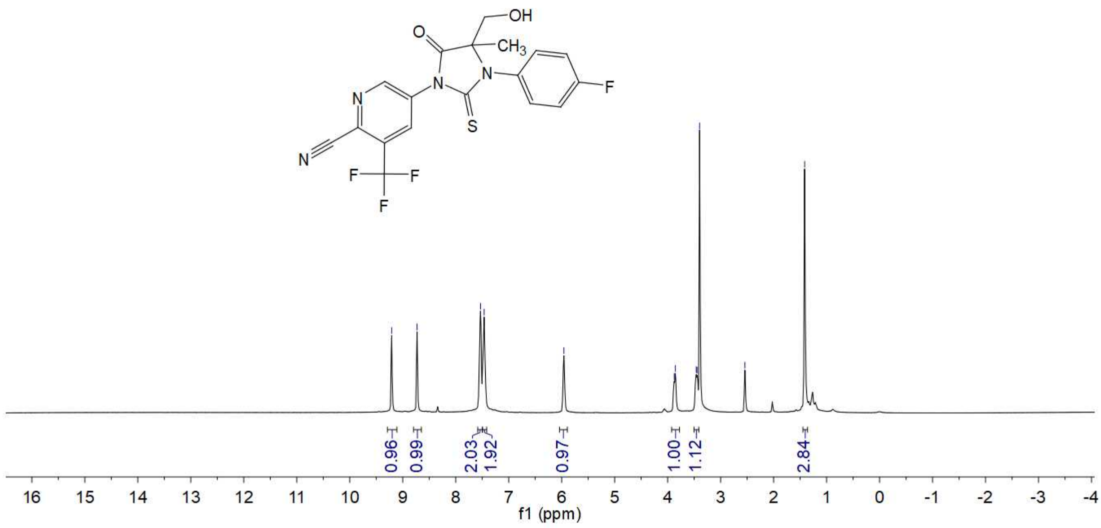

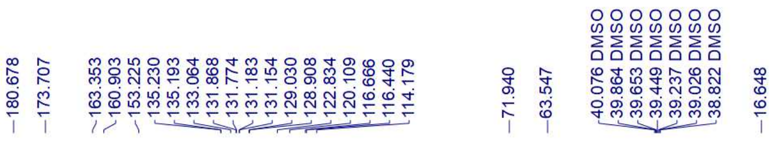

^{13}C NMR (101 MHz, DMSO) δ 180.68, 173.71, 163.35, 160.90, 153.23, 135.23, 135.19, 133.06, 131.87, 131.77, 131.18, 131.15, 129.03, 128.91, 122.83, 120.11, 116.67, 116.44, 114.18, 71.94, 63.55, 16.65.

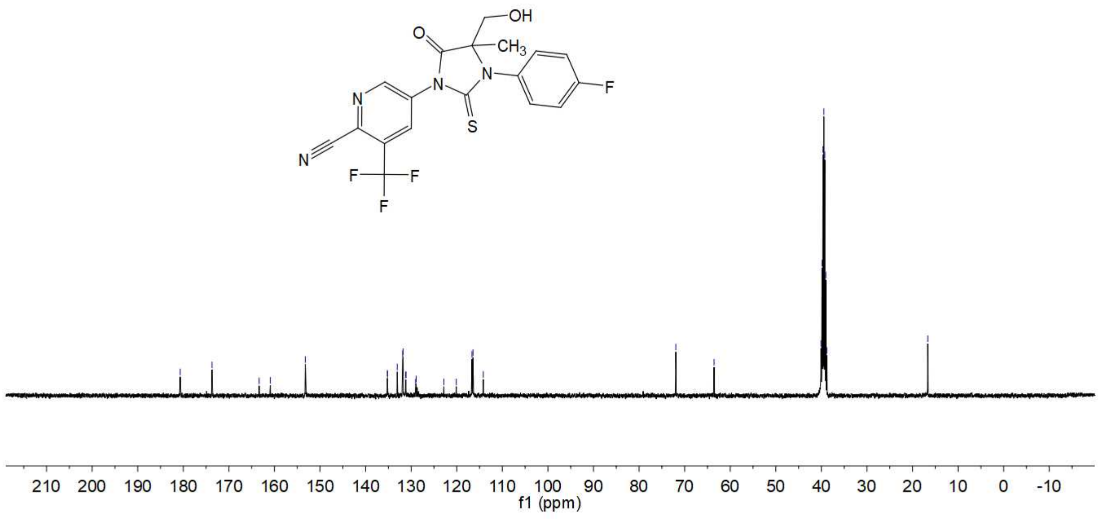

Single Mass Analysis
Tolerance = 500.0 PPM / DBE: min = -1.5, max = 50.0
Element prediction: Off

Monoisotopic Mass, Even Electron Ions
1 formula(e) evaluated with 1 results within limits (up to 50 closest results for each mass)
Elements Used:
C: 18-18 H: 13-13 N: 4-4 O: 2-2 F: 4-4 S: 1-1
JJY-A00222-061 31 (0.622)
1: TOF MS ES+

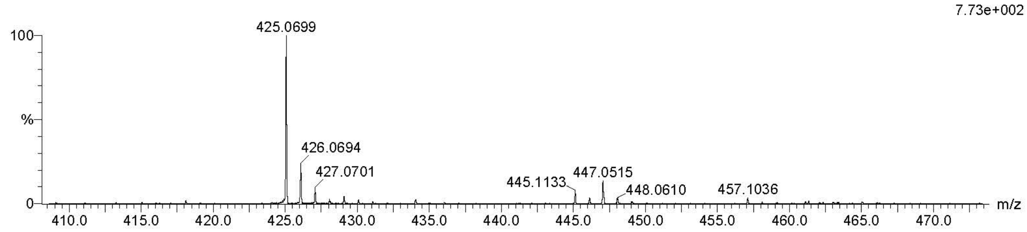

Mass	Calc. Mass	mDa	PPM	DBE	Formula
Minimum:				-1.5	
Maximum:		5.0	500.0	50.0	
425.0699	425.0695	0.4	0.9	12.5	C18 H13 N4 O2 F4 S

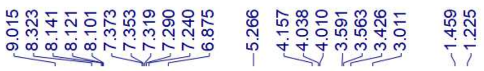

^{1}H NMR (400 MHz, $CDCl_3$) δ 9.01 (s, 1H), 8.32 (s, 1H), 8.12 (t, J = 7.9 Hz, 1H), 7.36 (d, J = 8.0 Hz, 1H), 7.30 (d, J = 11.5 Hz, 1H), 6.87 (s, 1H), 4.16 (s, 1H), 4.02 (d, J = 11.2 Hz, 1H), 3.58 (d, J = 11.2 Hz, 1H), 3.01 (s, 3H), 1.46 (s, 3H).

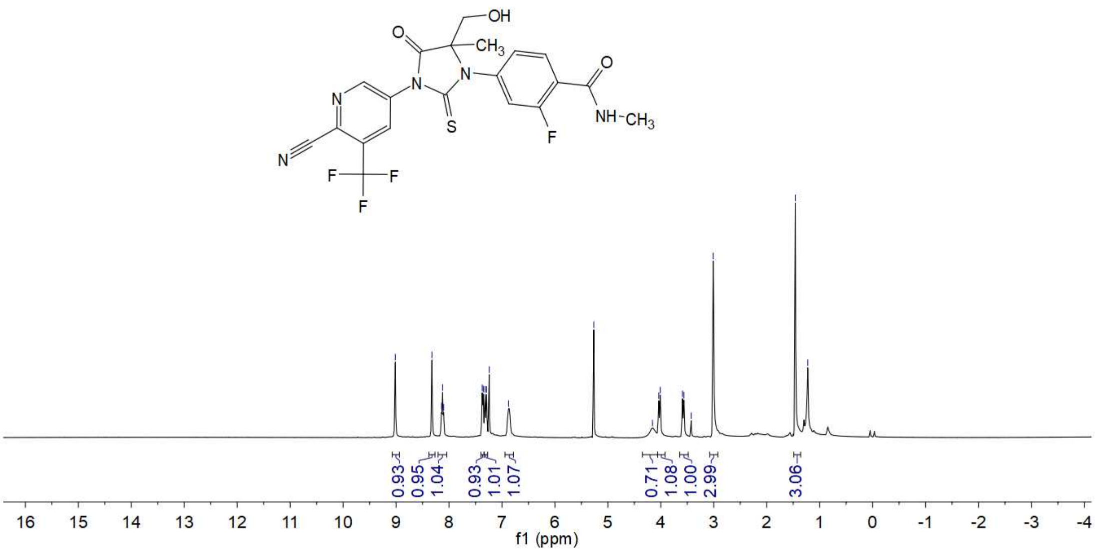

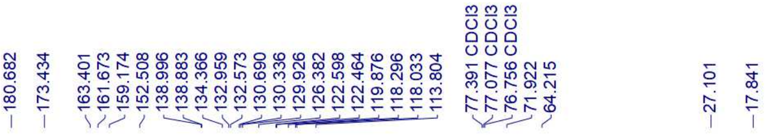

^{13}C NMR (101 MHz, $CDCl_3$) δ 180.68, 173.43, 163.40, 161.67, 159.17, 152.51, 139.00, 138.88, 134.37, 132.96, 132.57, 130.69, 130.34, 129.93, 126.38, 122.60, 122.46, 119.88, 118.30, 118.03, 113.80, 71.92, 64.21, 27.10, 17.84.

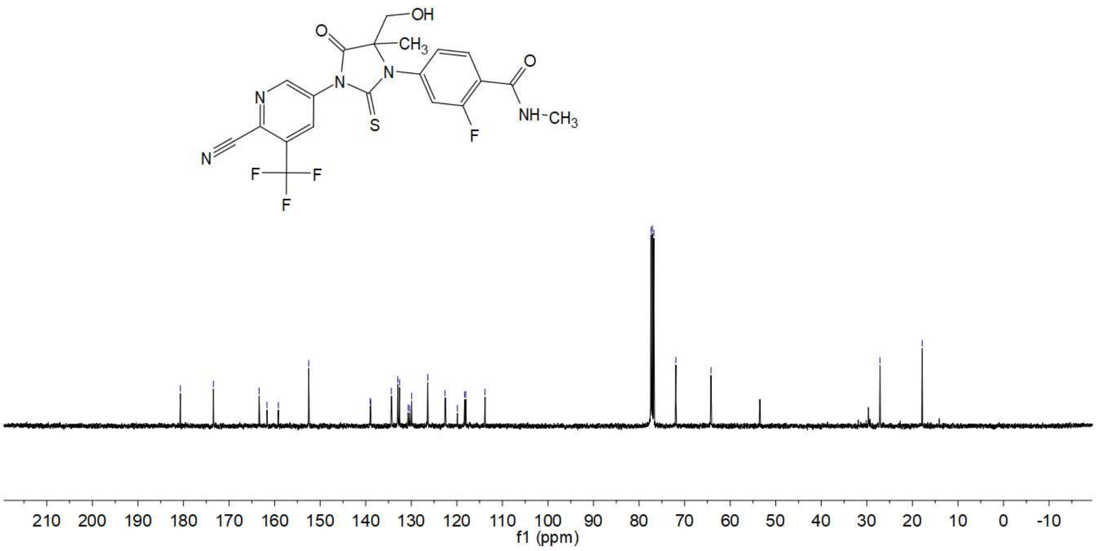

Elemental Composition Report

Single Mass Analysis

Tolerance = 500.0 PPM / DBE: min = -1.5, max = 50.0
Element prediction: Off

Monoisotopic Mass, Even Electron Ions
1 formula(e) evaluated with 1 results within limits (up to 50 closest results for each mass)
Elements Used:
C: 20-20 H: 16-16 N: 5-5 O: 3-3 F: 4-4 S: 1-1
JJY-A00222-063 35 (0.710)
1: TOF MS ES+

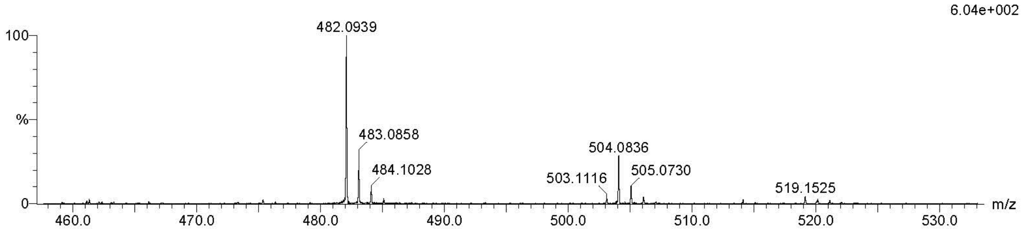

Minimum:				-1.5	
Maximum:		5.0	500.0	50.0	
Mass	Calc. Mass	mDa	PPM	DBE	Formula
482.0939	482.0910	2.9	6.0	13.5	C20 H16 N5 O3 F4 S

^{1}H NMR (400 MHz, $CDCl_3$) δ 9.01 (s, 1H), 8.29 (s, 1H), 7.27 (d, *J* = 8.5 Hz, 2H), 6.94 (d, *J* = 8.7 Hz, 2H), 4.33 (s, 1H), 4.00 (s, 1H), 3.90 (d, *J* = 11.0 Hz, 1H), 3.54 (d, *J* = 11.0 Hz, 1H), 2.70 (s, 2H), 2.37 (s, 2H), 2.29 (s, 3H), 2.00 (s, 2H), 1.83 (s, 2H), 1.38 (s, 3H).

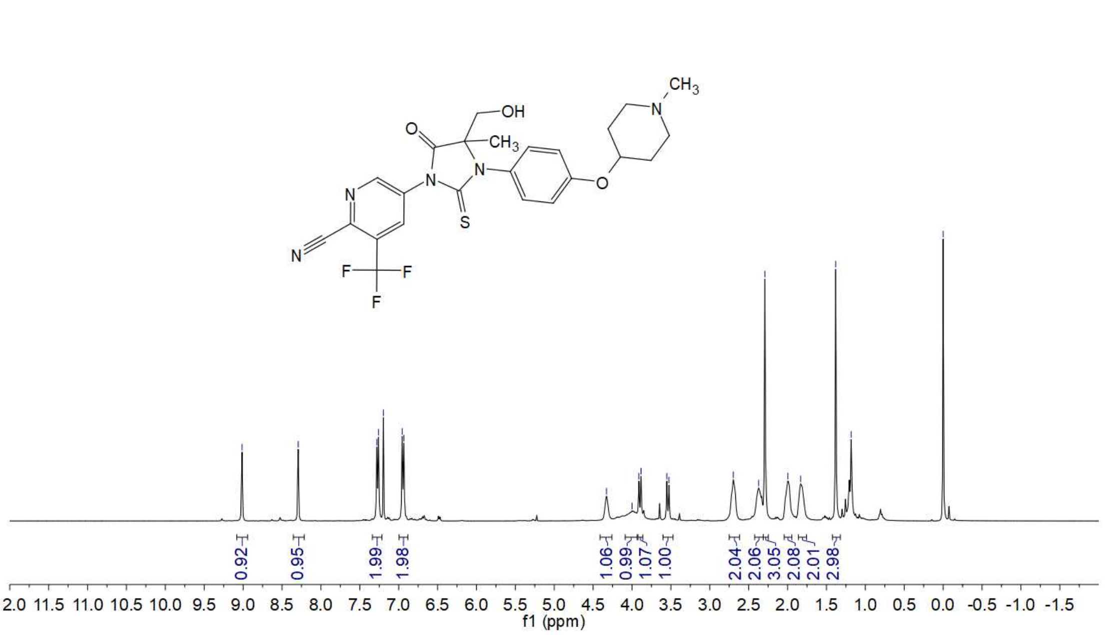

^{13}C NMR (101 MHz, $CDCl_3$) δ 179.95, 173.09, 157.20, 151.55, 133.28, 133.24, 131.82, 129.67, 129.57, 129.23, 128.75, 126.03, 121.67, 118.94, 115.58, 112.83, 76.24, 70.67, 62.93, 51.14, 44.75, 29.09, 16.80.

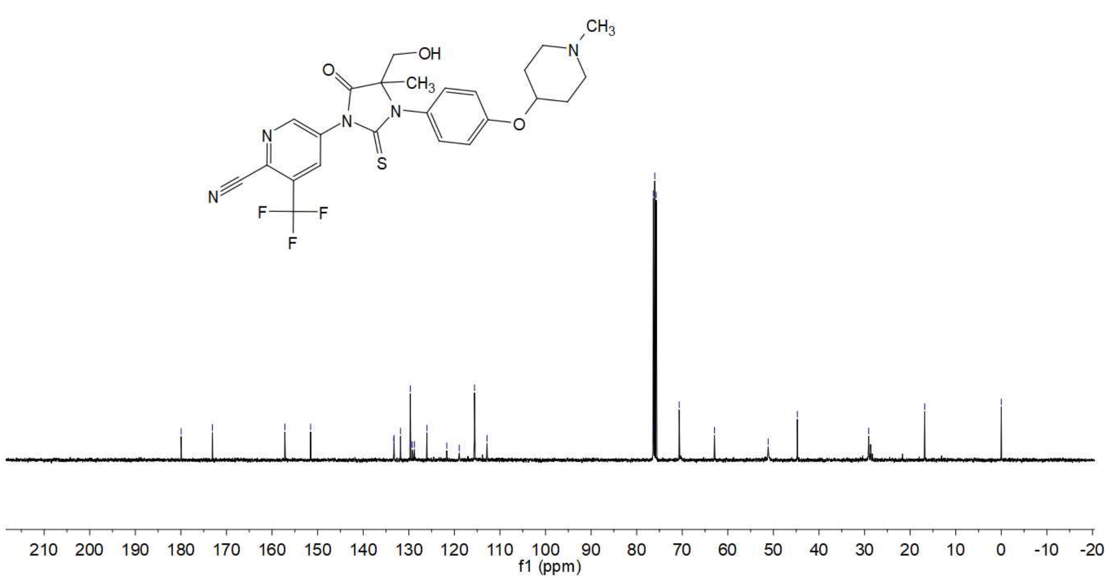

Elemental Composition Report

Single Mass Analysis

Tolerance = 500.0 PPM / DBE: min = -1.5, max = 50.0
Element prediction: Off

Monoisotopic Mass, Even Electron Ions
1 formula(e) evaluated with 1 results within limits (up to 50 closest results for each mass)
Elements Used:
C: 24-24 H: 25-25 N: 5-5 O: 3-3 F: 3-3 S: 1-1
JJY-A00222-064 41 (0.832)
1: TOF MS ES+

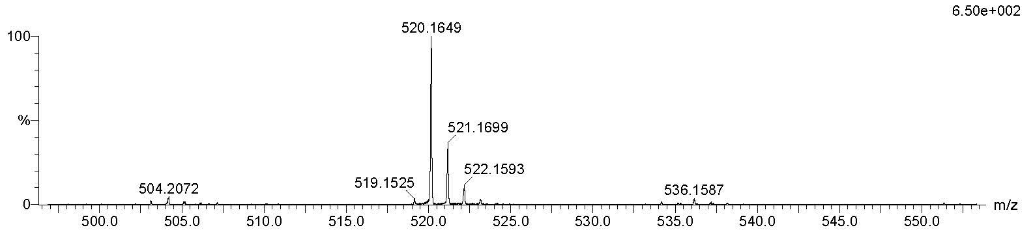

Mass	Calc. Mass	mDa	PPM	DBE	Formula
Minimum:				-1.5	
Maximum:		5.0	500.0	50.0	
520.1649	520.1630	1.9	3.7	13.5	C24 H25 N5 O3 F3 S
